# Whole genome comparison of a large collection of mycobacteriophages reveals a continuum of phage genetic diversity

**DOI:** 10.7554/eLife.06416

**Published:** 2015-04-28

**Authors:** Welkin H Pope, Charles A Bowman, Daniel A Russell, Deborah Jacobs-Sera, David J Asai, Steven G Cresawn, William R Jacobs, Roger W Hendrix, Jeffrey G Lawrence, Graham F Hatfull, Science Education Alliance Phage Hunters Advancing Genomics and Evolutionary Science, Patrick Abbazia, Phage Hunters Integrating Research and Education, Amma Ababio, Mycobacterial Genetics Course, Naazneen Adam

**Affiliations:** Biology and Chemistry, Nyack College, Nyack, United States; Xavier University of Louisiana, New Orleans, United States; Biology, Loyola Marymount University, Los Angeles, United States; Biology, University of California San Diego, La Jolla, United States; Biological Sciences, University of Pittsburgh, Pittsburgh, United States; Environmental and Biological Science, University of Maine, Machias, Machias, United States; Biology, Gettysburg College, Gettysburg, United States; Biological Sciences and Geology, Queensboro Community College, Bayside, United States; Purdue University, West Lafayette, United States; Microbiology, Miami University, Oxford, United States; Department of Biology, Baylor University, Waco, United States; Natural Sciences, Del Mar College, Corpus Christi, United States; Montclair State University, Montclair, United States; University of Maine, Honors College, Orono, United States; Biology, Spelman College, Atlanta, United States; School of Science and Technology, Georgia Gwinnett College, Lawrenceville, United States; Biology, Howard College, Washington, DC, United States; Biology, Saint Joseph's University, Philadelphia, United States; Biological Sciences, University of Pittsburgh, Pittsburgh, United States; Montclair State University, Montclair, United States; Biological Sciences, Carnegie Mellon University, Pittsburgh, United States; Biology, Gonzaga University, Spokane, United States; Purdue University, West Lafayette, United States; Virginia Commonwealth University, Richmond, United States; Morehouse College, Atlanta, United States; Biology, College of St. Scholastica, Duluth, United States; Microbiology, Miami University, Oxford, United States; Biology, Loyola Marymount University, Los Angeles, United States; Biology, University of Alabama Birmingham, Birmingham, United States; Biology and Chemistry, Nyack College, Nyack, United States; Biology, University of Wisconsin-River Falls, River Falls, United States; Biology, Carthage College, Kenosha, United States; Biology, University of California San Diego, La Jolla, United States; Biology, College of St. Scholastica, Duluth, United States; Biology, Howard College, Washington, DC, United States; Western Kentucky University, Bowling Green, United States; Biological Sciences, University of Mary Washington, Fredericksburg, United States; Biology, Calvin College, Grand Rapids, United States; Biology, University of Alabama Birmingham, Birmingham, United States; Department of Biology, Baylor University, Waco, United States; Biology, North Carolina Central University, Durham, United States; The Evergreen State College, Olympia, United States; Purdue University, West Lafayette, United States; Biology, Hope College, Holland, United States; Biological Sciences, Carnegie Mellon University, Pittsburgh, United States; Natural Sciences, University of Houston-Downtown, Houston, United States; Biology, University of Texas at El Paso, El Paso, United States; Natural Sciences, Del Mar College, Corpus Christi, United States; Biological Sciences, Carnegie Mellon University, Pittsburgh, United States; Department of Biology, Baylor University, Waco, United States; Biology, University of Puerto Rico - Cayey, Cayey, United States; Biology, Calvin College, Grand Rapids, United States; Montclair State University, Montclair, United States; Department of Biology, Baylor University, Waco, United States; Biology, College of William and Mary, Williamsburg, United States; Biology, Carthage College, Kenosha, United States; Biology, University of Texas at El Paso, El Paso, United States; Biology, University of Louisiana at Monroe, Monroe, United States; Biology, Carthage College, Kenosha, United States; Biology, University of Puerto Rico - Cayey, Cayey, United States; Biology, University of Puerto Rico - Cayey, Cayey, United States; Biology, Smith College, Northampton, United States; Microbiology and Biotechnology, North Carolina State University, Raleigh, United States; Biology, College of Charleston, Charleston, United States; Biology, Gonzaga University, Spokane, United States; Biology, Washington University in St. Louis, St. Louis, United States; Ohio State University, Columbus, United States; Biology, University of Louisiana at Monroe, Monroe, United States; Ohio State University, Columbus, United States; Virginia Commonwealth University, Richmond, United States; Biology, Washington University in St. Louis, St. Louis, United States; Biology, Gonzaga University, Spokane, United States; Biology, Washington University in St. Louis, St. Louis, United States; University of Maine, Honors College, Orono, United States; Biology, Loyola Marymount University, Los Angeles, United States; Biology, University of Louisiana at Monroe, Monroe, United States; Biology, Smith College, Northampton, United States; Biology, Gonzaga University, Spokane, United States; Biology, University of Wisconsin-River Falls, River Falls, United States; Biology, University of Louisiana at Monroe, Monroe, United States; Biology, University of Puerto Rico - Cayey, Cayey, United States; Biology, University of Texas at El Paso, El Paso, United States; The Evergreen State College, Olympia, United States; Ohio State University, Columbus, United States; Department of Microbiology, Immunology, and Molecular Genetics, University of California, Los Angeles, Los Angeles, United States; Biology, University of Louisiana at Monroe, Monroe, United States; Biology, College of St. Scholastica, Duluth, United States; Biology, Trinity College, Hartford, United States; Biology, University of California San Diego, La Jolla, United States; School of Science and Technology, Georgia Gwinnett College, Lawrenceville, United States; University of California Santa Cruz, Santa Cruz, United States; Microbiology and Molecular Biology, Brigham Young University, Provo, United States; Biology, Loyola Marymount University, Los Angeles, United States; Biology, Jacksonville State University, Jacksonville, United States; Biology, Calvin College, Grand Rapids, United States; Biology, Howard College, Washington, DC, United States; Xavier University of Louisiana, New Orleans, United States; University of Maine, Honors College, Orono, United States; Biology, Carthage College, Kenosha, United States; Biology, Illinois Wesleyan University, Bloomington, United States; Ohio State University, Columbus, United States; Biology, Illinois Wesleyan University, Bloomington, United States; Biological Sciences, Lehigh University, Bethlehem, United States; University of Maine, Honors College, Orono, United States; Environmental and Biological Science, University of Maine, Machias, Machias, United States; Biology, Ouachita Baptist University, Arkadelphia, United States; Honors Program, Florida Gulf Coast University, Fort Myers, United States; Biology, Loyola Marymount University, Los Angeles, United States; Biology, College of Charleston, Charleston, United States; Biology, College of Idaho, Caldwell, United States; Biology, College of St. Scholastica, Duluth, United States; Washington State University, Pullman, United States; Biology, Carthage College, Kenosha, United States; Providence College, Providence, United States; University of Florida, Gainsville, United States; Biology, Spelman College, Atlanta, United States; Biological Sciences, University of North Texas, Denton, United States; Biology, University of Puerto Rico - Cayey, Cayey, United States; Biology, Illinois Wesleyan University, Bloomington, United States; Montclair State University, Montclair, United States; Biological Sciences, University of North Texas, Denton, United States; Western Kentucky University, Bowling Green, United States; Washington State University, Pullman, United States; Biology, University of Louisiana at Monroe, Monroe, United States; Biology, College of St. Scholastica, Duluth, United States; Honors Program, Florida Gulf Coast University, Fort Myers, United States; Biology, Smith College, Northampton, United States; School of Science and Technology, Georgia Gwinnett College, Lawrenceville, United States; Biology, Howard College, Washington, DC, United States; Department of Microbiology, Immunology, and Molecular Genetics, University of California, Los Angeles, Los Angeles, United States; Biology, College of Charleston, Charleston, United States; Ohio State University, Columbus, United States; Microbiology, Miami University, Oxford, United States; Center for Life Science Education, Ohio State University, Columbus, United States; Biology, Hope College, Holland, United States; Biology, Smith College, Northampton, United States; Biology, University of Texas at El Paso, El Paso, United States; Biology, College of St. Scholastica, Duluth, United States; Xavier University of Louisiana, New Orleans, United States; Biology, Hampden-Sydney College, Farmville, United States; Biology, Washington University in St. Louis, St. Louis, United States; University of Florida, Gainsville, United States; Biological Sciences, Carnegie Mellon University, Pittsburgh, United States; Division of Natural and Health Sciences, Seton Hill University, Greensburg, United States; Biology, College of Charleston, Charleston, United States; Ohio State University, Columbus, United States; Howard Hughes Medical Institute, Chevy Chase, United States; Biology, University of Wisconsin-River Falls, River Falls, United States; Biology and Chemistry, Nyack College, Nyack, United States; Biology, North Carolina Central University, Durham, United States; Division of Natural and Health Sciences, Seton Hill University, Greensburg, United States; School of Science and Technology, Georgia Gwinnett College, Lawrenceville, United States; School of Science and Technology, Georgia Gwinnett College, Lawrenceville, United States; Biology, Calvin College, Grand Rapids, United States; Biology, Washington University in St. Louis, St. Louis, United States; Biology, Loyola Marymount University, Los Angeles, United States; University of Colorado at Boulder, Boulder, United States; Virginia Commonwealth University, Richmond, United States; Environmental and Biological Science, University of Maine, Machias, Machias, United States; University of Florida, Gainsville, United States; Biology, College of St. Scholastica, Duluth, United States; Biology, University of Alabama Birmingham, Birmingham, United States; Biology, Gonzaga University, Spokane, United States; Biology, University of Louisiana at Monroe, Monroe, United States; Biology, Gonzaga University, Spokane, United States; Biology, Spelman College, Atlanta, United States; Biological Sciences, Carnegie Mellon University, Pittsburgh, United States; Natural Sciences, Del Mar College, Corpus Christi, United States; University of Maine, Honors College, Orono, United States; University of Florida, Gainsville, United States; Department of Microbiology, Immunology, and Molecular Genetics, University of California, Los Angeles, Los Angeles, United States; Biology, University of Louisiana at Monroe, Monroe, United States; Biology, College of Charleston, Charleston, United States; Biology, Jacksonville State University, Jacksonville, United States; Washington State University, Pullman, United States; Biology, Illinois Wesleyan University, Bloomington, United States; Biology, Gonzaga University, Spokane, United States; Biology, Carthage College, Kenosha, United States; Biological Sciences, University of Mary Washington, Fredericksburg, United States; University of Maine, Honors College, Orono, United States; Biology, Calvin College, Grand Rapids, United States; University of California Santa Cruz, Santa Cruz, United States; Biology, Gonzaga University, Spokane, United States; Xavier University of Louisiana, New Orleans, United States; Biological Sciences, Lehigh University, Bethlehem, United States; Microbiology and Biotechnology, North Carolina State University, Raleigh, United States; Purdue University, West Lafayette, United States; Biological Sciences, University of North Texas, Denton, United States; Natural Sciences, University of Houston-Downtown, Houston, United States; Purdue University, West Lafayette, United States; Biology, Gonzaga University, Spokane, United States; The Evergreen State College, Olympia, United States; Biological Sciences, Lehigh University, Bethlehem, United States; Biology, Gettysburg College, Gettysburg, United States; University of Colorado at Boulder, Boulder, United States; Biological Sciences, Carnegie Mellon University, Pittsburgh, United States; Biology, Merrimack College, North Andover, United States; Biology, Gonzaga University, Spokane, United States; Biological Sciences, University of North Texas, Denton, United States; University of Florida, Gainsville, United States; Biology, Saint Joseph's University, Philadelphia, United States; Biology, Gonzaga University, Spokane, United States; Providence College, Providence, United States; Biology, University of Puerto Rico - Cayey, Cayey, United States; Biology, University of Puerto Rico - Cayey, Cayey, United States; Biology, University of Puerto Rico - Cayey, Cayey, United States; Biology, University of Louisiana at Monroe, Monroe, United States; Microbiology and Biotechnology, North Carolina State University, Raleigh, United States; Washington State University, Pullman, United States; Biology, Hope College, Holland, United States; Biology, Culver-Stockton College, Canton, United States; Biology, University of Wisconsin-River Falls, River Falls, United States; University of California Santa Cruz, Santa Cruz, United States; Biology, University of Puerto Rico - Cayey, Cayey, United States; Biology, University of Louisiana at Monroe, Monroe, United States; Biology, Gonzaga University, Spokane, United States; Biological Sciences and Geology, Queensboro Community College, Bayside, United States; Biology, College of Charleston, Charleston, United States; Biology, University of North Texas and University of Louisiana at Monroe, Monroe, United States; The Evergreen State College, Olympia, United States; Biology, Gonzaga University, Spokane, United States; Montclair State University, Montclair, United States; Biology, Washington University in St. Louis, St. Louis, United States; Biology, Trinity College, Hartford, United States; Western Kentucky University, Bowling Green, United States; Biology, University of Alabama Birmingham, Birmingham, United States; Morehouse College, Atlanta, United States; Biology, Gonzaga University, Spokane, United States; Western Kentucky University, Bowling Green, United States; Biology, Loyola Marymount University, Los Angeles, United States; Biological Sciences, University of Mary Washington, Fredericksburg, United States; Morehouse College, Atlanta, United States; Biology, Gettysburg College, Gettysburg, United States; University of Florida, Gainsville, United States; Biology, Saint Joseph's University, Philadelphia, United States; Biology, Hope College, Holland, United States; Microbiology and Biotechnology, North Carolina State University, Raleigh, United States; Biology, Illinois Wesleyan University, Bloomington, United States; Biology, Calvin College, Grand Rapids, United States; Biology, Gonzaga University, Spokane, United States; Biology, Illinois Wesleyan University, Bloomington, United States; Biology, Ouachita Baptist University, Arkadelphia, United States; Biology, University of Wisconsin-River Falls, River Falls, United States; Washington State University, Pullman, United States; Biology, University of Louisiana at Monroe, Monroe, United States; Biology, Gettysburg College, Gettysburg, United States; Biology, University of Texas at El Paso, El Paso, United States; Biology, Washington University in St. Louis, St. Louis, United States; Biology, Washington University in St. Louis, St. Louis, United States; Biology, Illinois Wesleyan University, Bloomington, United States; University of Florida, Gainsville, United States; Arts and Sciences Division, University of Maine, Fort Kent, Fort Kent, United States; Biology, University of Texas at El Paso, El Paso, United States; Biological Sciences and Geology, Queensboro Community College, Bayside, United States; Montclair State University, Montclair, United States; Biology, University of Louisiana at Monroe, Monroe, United States; Biology, Washington University in St. Louis, St. Louis, United States; Biology, Hope College, Holland, United States; Biological Sciences, Lehigh University, Bethlehem, United States; Department of Biology, Baylor University, Waco, United States; Howard Hughes Medical Institute, Chevy Chase, United States; Morehouse College, Atlanta, United States; Biology, Ouachita Baptist University, Arkadelphia, United States; Environmental and Biological Science, University of Maine, Machias, Machias, United States; Biology, Carthage College, Kenosha, United States; Biology, Saint Joseph's University, Philadelphia, United States; Biology, Jacksonville State University, Jacksonville, United States; Biology, University of Louisiana at Monroe, Monroe, United States; Biology, University of Louisiana at Monroe, Monroe, United States; Department of Biology, Baylor University, Waco, United States; Biology, University of Wisconsin-River Falls, River Falls, United States; University of Florida, Gainsville, United States; Biological Sciences, Carnegie Mellon University, Pittsburgh, United States; Microbiology and Biotechnology, North Carolina State University, Raleigh, United States; Microbiology and Molecular Biology, Brigham Young University, Provo, United States; Biology, Gonzaga University, Spokane, United States; Biology, Loyola Marymount University, Los Angeles, United States; Chemistry and Biochemistry, Ohio State University, Columbus, United States; Washington State University, Pullman, United States; Purdue University, West Lafayette, United States; Biology, Jacksonville State University, Jacksonville, United States; Biology, Nebraska Wesleyan University, Lincoln, Nebraska, United States; Department of Biology, Baylor University, Waco, United States; Microbiology and Molecular Biology, Brigham Young University, Provo, United States; Biology, Calvin College, Grand Rapids, United States; Biology, University of Louisiana at Monroe, Monroe, United States; Biology, College of Charleston, Charleston, United States; Biology, Howard College, Washington, DC, United States; Biology, Illinois Wesleyan University, Bloomington, United States; Biology, North Carolina Central University, Durham, United States; Biological Sciences, University of Pittsburgh, Pittsburgh, United States; Ohio State University, Columbus, United States; Morehouse College, Atlanta, United States; Microbiology and Biotechnology, North Carolina State University, Raleigh, United States; School of Science and Technology, Georgia Gwinnett College, Lawrenceville, United States; Providence College, Providence, United States; Biology, Washington University in St. Louis, St. Louis, United States; Biology, Washington University in St. Louis, St. Louis, United States; Biology, Howard College, Washington, DC, United States; Biology, Carthage College, Kenosha, United States; Biology, Gettysburg College, Gettysburg, United States; Virginia Commonwealth University, Richmond, United States; Ohio State University, Columbus, United States; Montclair State University, Montclair, United States; Biological Sciences, University of North Texas, Denton, United States; University of California Santa Cruz, Santa Cruz, United States; University of California Santa Cruz, Santa Cruz, United States; Microbiology, Miami University, Oxford, United States; Biology, Gonzaga University, Spokane, United States; Biology, College of Charleston, Charleston, United States; Biology, University of Wisconsin-River Falls, River Falls, United States; Biology, Saint Joseph's University, Philadelphia, United States; Biology, Nebraska Wesleyan University, Lincoln, Nebraska, United States; Biology, University of California San Diego, La Jolla, United States; Microbiology and Biotechnology, North Carolina State University, Raleigh, United States; Biology, Trinity College, Hartford, United States; Providence College, Providence, United States; The Evergreen State College, Olympia, United States; Montclair State University, Montclair, United States; Biology, Calvin College, Grand Rapids, United States; Biology, Calvin College, Grand Rapids, United States; Biological Sciences, University of Pittsburgh, Pittsburgh, United States; The Evergreen State College, Olympia, United States; Biotechnology, James Madison University, Harrisonburg, United States; The Evergreen State College, Olympia, United States; Microbiology and Molecular Biology, Brigham Young University, Provo, United States; Biological Sciences, University of North Texas, Denton, United States; Biology, North Carolina Central University, Durham, United States; Biology, Carthage College, Kenosha, United States; Biology, College of St. Scholastica, Duluth, United States; Ohio State University, Columbus, United States; Biology, Carthage College, Kenosha, United States; Biology, Howard College, Washington, DC, United States; Division of Natural and Health Sciences, Seton Hill University, Greensburg, United States; Biology, University of California San Diego, La Jolla, United States; Biology, Howard College, Washington, DC, United States; Biological Sciences, University of Pittsburgh, Pittsburgh, United States; Biology, Carthage College, Kenosha, United States; Biological Sciences, Carnegie Mellon University, Pittsburgh, United States; Biology, University of Puerto Rico - Cayey, Cayey, United States; Biology, College of St. Scholastica, Duluth, United States; Biology, University of Alabama Birmingham, Birmingham, United States; Montclair State University, Montclair, United States; Biology, Montana Tech of the University of Montana, Butte, United States; Biology, College of Charleston, Charleston, United States; Biological Sciences and Geology, Queensboro Community College, Bayside, United States; Biology, Illinois Wesleyan University, Bloomington, United States; Biology, Saint Joseph's University, Philadelphia, United States; Biology, Gonzaga University, Spokane, United States; Biology, Washington University in St. Louis, St. Louis, United States; Western Kentucky University, Bowling Green, United States; Biology, University of Texas at El Paso, El Paso, United States; Biotechnology, Southern Maine Community College, South Portland, United States; Biology, Gettysburg College, Gettysburg, United States; Biological Sciences, Carnegie Mellon University, Pittsburgh, United States; School of Science and Technology, Georgia Gwinnett College, Lawrenceville, United States; Biology, Howard College, Washington, DC, United States; Virginia Commonwealth University, Richmond, United States; Biology, Illinois Wesleyan University, Bloomington, United States; Montclair State University, Montclair, United States; Biology, University of Puerto Rico - Cayey, Cayey, United States; Western Kentucky University, Bowling Green, United States; Biology and Chemistry, Nyack College, Nyack, United States; Biology, Gettysburg College, Gettysburg, United States; Biology, Jacksonville State University, Jacksonville, United States; Biology, Gonzaga University, Spokane, United States; Purdue University, West Lafayette, United States; Purdue University, West Lafayette, United States; Biology, CUNY, Queens College, Queens, United States; Biology, Gonzaga University, Spokane, United States; Biology, Smith College, Northampton, United States; University of California Santa Cruz, Santa Cruz, United States; Microbiology and Biotechnology, North Carolina State University, Raleigh, United States; Biology, Illinois Wesleyan University, Bloomington, United States; Microbiology, Miami University, Oxford, United States; The Evergreen State College, Olympia, United States; Microbiology and Biotechnology, North Carolina State University, Raleigh, United States; Department of Biological Sciences, University of Maryland, Baltimore County, Baltimore, United States; Science, Cabrini College, Radnor, United States; Providence College, Providence, United States; Xavier University of Louisiana, New Orleans, United States; Biology, University of Puerto Rico - Cayey, Cayey, United States; Washington State University, Pullman, United States; Providence College, Providence, United States; Biology, University of Texas at El Paso, El Paso, United States; Biology, University of Texas at El Paso, El Paso, United States; Biology, North Carolina Central University, Durham, United States; Biology, Calvin College, Grand Rapids, United States; Biology, Gonzaga University, Spokane, United States; Biology, Washington University in St. Louis, St. Louis, United States; Biology, Hampden-Sydney College, Farmville, United States; Providence College, Providence, United States; Purdue University, West Lafayette, United States; Biology, Loyola Marymount University, Los Angeles, United States; Morehouse College, Atlanta, United States; University of Colorado at Boulder, Boulder, United States; Biology, University of Texas at El Paso, El Paso, United States; Biology, College of Idaho, Caldwell, United States; Biology, Trinity College, Hartford, United States; Biology, University of California San Diego, La Jolla, United States; Virginia Commonwealth University, Richmond, United States; Purdue University, West Lafayette, United States; Biology, Illinois Wesleyan University, Bloomington, United States; Biology, Loyola Marymount University, Los Angeles, United States; Biology, Washington University in St. Louis, St. Louis, United States; Biology, University of Alabama Birmingham, Birmingham, United States; Biology, University of Texas at El Paso, El Paso, United States; Microbiology and Biotechnology, North Carolina State University, Raleigh, United States; The Evergreen State College, Olympia, United States; Biology, College of Idaho, Caldwell, United States; Microbiology, Miami University, Oxford, United States; Biological Sciences and Geology, Queensboro Community College, Bayside, United States; Biology, University of Texas at El Paso, El Paso, United States; Biology, University of California San Diego, La Jolla, United States; Department of Microbiology, Immunology, and Molecular Genetics, University of California, Los Angeles, Los Angeles, United States; Biology, Washington University in St. Louis, St. Louis, United States; Biological Sciences and Geology, Queensboro Community College, Bayside, United States; Biological Sciences and Geology, Queensboro Community College, Bayside, United States; Biology, University of California San Diego, La Jolla, United States; Division of Natural and Health Sciences, Seton Hill University, Greensburg, United States; Biology, North Carolina Central University, Durham, United States; Biology, University of California San Diego, La Jolla, United States; Biology and Chemistry, Nyack College, Nyack, United States; Biology, University of California San Diego, La Jolla, United States; Biology, Montana Tech of the University of Montana, Butte, United States; Virginia Commonwealth University, Richmond, United States; Department of Biology, Baylor University, Waco, United States; Biological Sciences, Carnegie Mellon University, Pittsburgh, United States; Biology, University of California San Diego, La Jolla, United States; Department of Microbiology, Immunology, and Molecular Genetics, University of California, Los Angeles, Los Angeles, United States; Biological Sciences, University of North Texas, Denton, United States; Biology, Trinity College, Hartford, United States; Biological Sciences, University of Pittsburgh, Pittsburgh, United States; Southern Connecticut State University, New Haven, United States; Biology, Washington University in St. Louis, St. Louis, United States; Biology, University of California San Diego, La Jolla, United States; Science, Cabrini College, Radnor, United States; Biology, University of Louisiana at Monroe, Monroe, United States; Biology, Gonzaga University, Spokane, United States; Biology, University of Texas at El Paso, El Paso, United States; Purdue University, West Lafayette, United States; Doane College, Crete, United States; University of California Santa Cruz, Santa Cruz, United States; Biology, Howard College, Washington, DC, United States; Department of Biology, Baylor University, Waco, United States; Biological Sciences, University of North Texas, Denton, United States; Biology, Smith College, Northampton, United States; Ohio State University, Columbus, United States; Biology, Gonzaga University, Spokane, United States; Biological Sciences, University of Pittsburgh, Pittsburgh, United States; Biological Sciences, Carnegie Mellon University, Pittsburgh, United States; Biology, University of California San Diego, La Jolla, United States; Biology, University of Alabama Birmingham, Birmingham, United States; Biology, Gettysburg College, Gettysburg, United States; Biology, University of Wisconsin-River Falls, River Falls, United States; Biology, Spelman College, Atlanta, United States; Biological Sciences, University of Pittsburgh, Pittsburgh, United States; The Evergreen State College, Olympia, United States; Biology, Saint Joseph's University, Philadelphia, United States; Biology, Saint Joseph's University, Philadelphia, United States; Biology, Hope College, Holland, United States; Biological Sciences, Lehigh University, Bethlehem, United States; Biology, Gonzaga University, Spokane, United States; Biological Sciences, Lehigh University, Bethlehem, United States; Department of Biology, Baylor University, Waco, United States; Ohio State University, Columbus, United States; Morehouse College, Atlanta, United States; Biology, Gonzaga University, Spokane, United States; Washington State University, Pullman, United States; Biology, Gonzaga University, Spokane, United States; The Evergreen State College, Olympia, United States; Biology, Gonzaga University, Spokane, United States; Biotechnology, Southern Maine Community College, South Portland, United States; Ohio State University, Columbus, United States; Biology, Loyola Marymount University, Los Angeles, United States; Biology, Loyola Marymount University, Los Angeles, United States; University of Colorado at Boulder, Boulder, United States; Biology, Jacksonville State University, Jacksonville, United States; University of Maine, Honors College, Orono, United States; Western Kentucky University, Bowling Green, United States; Marine Science, Southern Maine Community College, South Portland, United States; University of Colorado at Boulder, Boulder, United States; Biology, Saint Joseph's University, Philadelphia, United States; Biology, University of Puerto Rico - Cayey, Cayey, United States; Providence College, Providence, United States; Providence College, Providence, United States; Biology, University of Puerto Rico - Cayey, Cayey, United States; Morehouse College, Atlanta, United States; Biology, University of Texas at El Paso, El Paso, United States; Southern Connecticut State University, New Haven, United States; Biology, University of Puerto Rico - Cayey, Cayey, United States; Biology, University of Puerto Rico - Cayey, Cayey, United States; Biology, Loyola Marymount University, Los Angeles, United States; Western Kentucky University, Bowling Green, United States; Purdue University, West Lafayette, United States; Purdue University, West Lafayette, United States; Biology, Trinity College, Hartford, United States; Biology, University of Louisiana at Monroe, Monroe, United States; Biological Sciences, University of Mary Washington, Fredericksburg, United States; Biology, Smith College, Northampton, United States; Biology, University of Puerto Rico - Cayey, Cayey, United States; Biology, Loyola Marymount University, Los Angeles, United States; Science, Cabrini College, Radnor, United States; The Evergreen State College, Olympia, United States; Biology, University of Puerto Rico - Cayey, Cayey, United States; Biology, Washington University in St. Louis, St. Louis, United States; Biology, College of St. Scholastica, Duluth, United States; Providence College, Providence, United States; Biology, Illinois Wesleyan University, Bloomington, United States; Biological Sciences, University of North Texas, Denton, United States; Biology, Gonzaga University, Spokane, United States; Biology, Gonzaga University, Spokane, United States; Biology, Loyola Marymount University, Los Angeles, United States; Morehouse College, Atlanta, United States; Biology, College of St. Scholastica, Duluth, United States; Biology, Trinity College, Hartford, United States; Biology, Saint Joseph's University, Philadelphia, United States; Biology, University of Texas at El Paso, El Paso, United States; Biology, Trinity College, Hartford, United States; Biology and Chemistry, Nyack College, Nyack, United States; Science, Cabrini College, Radnor, United States; Biological Sciences, Carnegie Mellon University, Pittsburgh, United States; Biology, Illinois Wesleyan University, Bloomington, United States; Honors Program, Florida Gulf Coast University, Fort Myers, United States; Purdue University, West Lafayette, United States; Biology, Hope College, Holland, United States; Biology, University of Louisiana at Monroe, Monroe, United States; University of Colorado at Boulder, Boulder, United States; Biology, Loyola Marymount University, Los Angeles, United States; Biology, Trinity College, Hartford, United States; Biology, University of Louisiana at Monroe, Monroe, United States; Department of Biology, Baylor University, Waco, United States; Xavier University of Louisiana, New Orleans, United States; Biology, Smith College, Northampton, United States; Microbiology, Ohio State University, Columbus, United States; Biology, College of Idaho, Caldwell, United States; Science, Cabrini College, Radnor, United States; Biological Sciences, Carnegie Mellon University, Pittsburgh, United States; University of California Santa Cruz, Santa Cruz, United States; Biology, Howard College, Washington, DC, United States; Microbiology, Miami University, Oxford, United States; Biological Sciences, Carnegie Mellon University, Pittsburgh, United States; Biology and Chemistry, Nyack College, Nyack, United States; Biological Sciences, University of North Texas, Denton, United States; Biology, Gonzaga University, Spokane, United States; Biology, Gonzaga University, Spokane, United States; Biology, University of Alabama Birmingham, Birmingham, United States; Biology, University of Louisiana at Monroe, Monroe, United States; Biology, University of Louisiana at Monroe, Monroe, United States; Biology, University of Louisiana at Monroe, Monroe, United States; Biology, Washington University in St. Louis, St. Louis, United States; University of California Santa Cruz, Santa Cruz, United States; Biology, Gonzaga University, Spokane, United States; Washington State University, Pullman, United States; Biology, Calvin College, Grand Rapids, United States; Biological Sciences and Geology, Queensboro Community College, Bayside, United States; Biology, Hope College, Holland, United States; Biology, Culver-Stockton College, Canton, United States; Biology, Loyola Marymount University, Los Angeles, United States; Biology, University of Texas at El Paso, El Paso, United States; University of California Santa Cruz, Santa Cruz, United States; University of California Santa Cruz, Santa Cruz, United States; Biology, Saint Joseph's University, Philadelphia, United States; Biology, Jacksonville State University, Jacksonville, United States; Biology, Carthage College, Kenosha, United States; Biology, Washington University in St. Louis, St. Louis, United States; Biology, Hope College, Holland, United States; Environmental and Biological Science, University of Maine, Machias, Machias, United States; Microbiology, Miami University, Oxford, United States; Biology, Calvin College, Grand Rapids, United States; Morehouse College, Atlanta, United States; Biology, Gettysburg College, Gettysburg, United States; Biology, Gettysburg College, Gettysburg, United States; Biology, Loyola Marymount University, Los Angeles, United States; Biology and Chemistry, Nyack College, Nyack, United States; Purdue University, West Lafayette, United States; Biological Sciences, University of North Texas, Denton, United States; Biology, College of St. Scholastica, Duluth, United States; Honors Program, Florida Gulf Coast University, Fort Myers, United States; Biology, Washington University in St. Louis, St. Louis, United States; Biology, Saint Joseph's University, Philadelphia, United States; Biology, CUNY, Queens College, Queens, United States; Washington State University, Pullman, United States; Biology, Howard College, Washington, DC, United States; Integrative Biology, Oregon State University, Corvallis, United States; Ohio State University, Columbus, United States; Biology, University of California San Diego, La Jolla, United States; Biological Sciences, Lehigh University, Bethlehem, United States; Biology, Smith College, Northampton, United States; Biology, Washington University in St. Louis, St. Louis, United States; Biological Sciences, University of North Texas, Denton, United States; Biology and Chemistry, Nyack College, Nyack, United States; Biology, Smith College, Northampton, United States; Biology, Washington University in St. Louis, St. Louis, United States; Biology, Calvin College, Grand Rapids, United States; Biology, Nebraska Wesleyan University, Lincoln, Nebraska, United States; Department of Microbiology, Immunology, and Molecular Genetics, University of California, Los Angeles, Los Angeles, United States; Washington State University, Pullman, United States; Biology, Illinois Wesleyan University, Bloomington, United States; Biology, University of Puerto Rico - Cayey, Cayey, United States; Biology, Gettysburg College, Gettysburg, United States; Biology, Howard College, Washington, DC, United States; Biology, Culver-Stockton College, Canton, United States; Biology, Hampden-Sydney College, Farmville, United States; Biology, Trinity College, Hartford, United States; Biological Sciences, Carnegie Mellon University, Pittsburgh, United States; Montclair State University, Montclair, United States; University of California Santa Cruz, Santa Cruz, United States; Biology, Merrimack College, North Andover, United States; Microbiology and Biotechnology, North Carolina State University, Raleigh, United States; University of Colorado at Boulder, Boulder, United States; Western Kentucky University, Bowling Green, United States; Biology, College of Idaho, Caldwell, United States; Southern Connecticut State University, New Haven, United States; Ohio State University, Columbus, United States; University of Florida, Gainsville, United States; Biology, University of Puerto Rico - Cayey, Cayey, United States; Microbiology, Miami University, Oxford, United States; University of California Santa Cruz, Santa Cruz, United States; Biology, Trinity College, Hartford, United States; Biology, University of Texas at El Paso, El Paso, United States; Biology, Smith College, Northampton, United States; Biology, Hope College, Holland, United States; School of Science and Technology, Georgia Gwinnett College, Lawrenceville, United States; Department of Microbiology, Immunology, and Molecular Genetics, University of California, Los Angeles, Los Angeles, United States; Western Kentucky University, Bowling Green, United States; Xavier University of Louisiana, New Orleans, United States; Doane College, Crete, United States; Science, Cabrini College, Radnor, United States; Biology and Medicine, Brown University, Providence, United States; Western Kentucky University, Bowling Green, United States; Department of Biology, Baylor University, Waco, United States; Biology, University of California San Diego, La Jolla, United States; Providence College, Providence, United States; Biology, University of Alabama Birmingham, Birmingham, United States; Biology, Carthage College, Kenosha, United States; Biology, College of Charleston, Charleston, United States; Biology, Culver-Stockton College, Canton, United States; Biology, University of Wisconsin-River Falls, River Falls, United States; Science, Cabrini College, Radnor, United States; Biology, University of Alabama Birmingham, Birmingham, United States; Environmental and Biological Science, University of Maine, Machias, Machias, United States; Microbiology, Miami University, Oxford, United States; Biology, Gettysburg College, Gettysburg, United States; Biology, University of Louisiana at Monroe, Monroe, United States; The Evergreen State College, Olympia, United States; Biology, University of Louisiana at Monroe, Monroe, United States; Washington State University, Pullman, United States; Department of Biology, Baylor University, Waco, United States; Biology, Calvin College, Grand Rapids, United States; Biological Sciences and Geology, Queensboro Community College, Bayside, United States; University of Colorado at Boulder, Boulder, United States; Microbiology and Molecular Biology, Brigham Young University, Provo, United States; Biology, Washington University in St. Louis, St. Louis, United States; Biology, Calvin College, Grand Rapids, United States; Biology, Loyola Marymount University, Los Angeles, United States; Biology, Calvin College, Grand Rapids, United States; Biology, Southern Connecticut State University, New Haven, United States; Biology, Illinois Wesleyan University, Bloomington, United States; Biology, Hope College, Holland, United States; Biology, Saint Joseph's University, Philadelphia, United States; Biology, College of Charleston, Charleston, United States; University of Colorado at Boulder, Boulder, United States; Biology, Washington University in St. Louis, St. Louis, United States; Honors Program, Florida Gulf Coast University, Fort Myers, United States; Biology, Saint Joseph's University, Philadelphia, United States; Biology, University of Louisiana at Monroe, Monroe, United States; Montclair State University, Montclair, United States; Biology, Ouachita Baptist University, Arkadelphia, United States; Southern Connecticut State University, New Haven, United States; Biology, College of Charleston, Charleston, United States; Montclair State University, Montclair, United States; Biology, Loyola Marymount University, Los Angeles, United States; Biology, Illinois Wesleyan University, Bloomington, United States; Biology, Ouachita Baptist University, Arkadelphia, United States; Biology, University of Louisiana at Monroe, Monroe, United States; Microbiology and Molecular Biology, Brigham Young University, Provo, United States; Biological Sciences, Carnegie Mellon University, Pittsburgh, United States; Biological Sciences and Geology, Queensboro Community College, Bayside, United States; Biology, Hope College, Holland, United States; Biology, University of California San Diego, La Jolla, United States; University of Colorado at Boulder, Boulder, United States; Biology, Washington University in St. Louis, St. Louis, United States; Division of Natural and Health Sciences, Seton Hill University, Greensburg, United States; University of California Santa Cruz, Santa Cruz, United States; Biological Sciences and Geology, Queensboro Community College, Bayside, United States; Biology, University of Puerto Rico - Cayey, Cayey, United States; Biology, Loyola Marymount University, Los Angeles, United States; Biology, CUNY, Queens College, Queens, United States; Biology, University of Texas at El Paso, El Paso, United States; Xavier University of Louisiana, New Orleans, United States; Biotechnology, Southern Maine Community College, South Portland, United States; Biology, University of California San Diego, La Jolla, United States; Biology, University of Puerto Rico - Cayey, Cayey, United States; Biology, Gonzaga University, Spokane, United States; Biology, University of California San Diego, La Jolla, United States; Ohio State University, Columbus, United States; Biology, Washington University in St. Louis, St. Louis, United States; Biology, Calvin College, Grand Rapids, United States; Microbiology, Miami University, Oxford, United States; Biology, Saint Joseph's University, Philadelphia, United States; Biology, Howard College, Washington, DC, United States; Biology, Howard College, Washington, DC, United States; Biology, Calvin College, Grand Rapids, United States; Biology, University of Alabama Birmingham, Birmingham, United States; Biology, University of Wisconsin-River Falls, River Falls, United States; Biological Sciences, Carnegie Mellon University, Pittsburgh, United States; Biology, Loyola Marymount University, Los Angeles, United States; Biology, Gonzaga University, Spokane, United States; Science, Cabrini College, Radnor, United States; Biology, University of California San Diego, La Jolla, United States; Biological Sciences, University of North Texas, Denton, United States; University of California Santa Cruz, Santa Cruz, United States; Biology, Howard College, Washington, DC, United States; Biology, University of Texas at El Paso, El Paso, United States; ISBT, LaSalle University, Philadelphia, United States; Biological Sciences, University of Pittsburgh, Pittsburgh, United States; Purdue University, West Lafayette, United States; Western Kentucky University, Bowling Green, United States; University of California Santa Cruz, Santa Cruz, United States; Biological Sciences, Lehigh University, Bethlehem, United States; Biological Sciences, Lehigh University, Bethlehem, United States; Biology, Calvin College, Grand Rapids, United States; Biology, Carthage College, Kenosha, United States; Biology, University of Puerto Rico - Cayey, Cayey, United States; University of Colorado at Boulder, Boulder, United States; Division of Natural and Health Sciences, Seton Hill University, Greensburg, United States; Biological Sciences, Carnegie Mellon University, Pittsburgh, United States; Xavier University of Louisiana, New Orleans, United States; University of Florida, Gainsville, United States; Pedagogy, University of Puerto Rico - Cayey, Cayey, United States; Biology, University of Puerto Rico - Cayey, Cayey, United States; Providence College, Providence, United States; Biology and Medicine, Brown University, Providence, United States; Biology, Saint Joseph's University, Philadelphia, United States; Biological Sciences, Lehigh University, Bethlehem, United States; Biology, University of Louisiana at Monroe, Monroe, United States; Biological Sciences, Lehigh University, Bethlehem, United States; Biological Sciences, Carnegie Mellon University, Pittsburgh, United States; University of Colorado at Boulder, Boulder, United States; Ohio State University, Columbus, United States; Biology, University of Louisiana at Monroe, Monroe, United States; Biology, Montana Tech of the University of Montana, Butte, United States; Montclair State University, Montclair, United States; University of Colorado at Boulder, Boulder, United States; Microbiology and Molecular Biology, Brigham Young University, Provo, United States; University of Florida, Gainsville, United States; Biology, Merrimack College, North Andover, United States; Biology, Gonzaga University, Spokane, United States; Biology, Calvin College, Grand Rapids, United States; Biology, Trinity College, Hartford, United States; School of Science and Technology, Georgia Gwinnett College, Lawrenceville, United States; Natural Sciences, Del Mar College, Corpus Christi, United States; Biological Sciences, University of North Texas, Denton, United States; Xavier University of Louisiana, New Orleans, United States; Biological Sciences, Carnegie Mellon University, Pittsburgh, United States; Morehouse College, Atlanta, United States; Biology, Gonzaga University, Spokane, United States; Biology, Gettysburg College, Gettysburg, United States; Department of Microbiology, Immunology, and Molecular Genetics, University of California, Los Angeles, Los Angeles, United States; Biological Sciences, Carnegie Mellon University, Pittsburgh, United States; Biology, University of Louisiana at Monroe, Monroe, United States; Biology, University of Louisiana at Monroe, Monroe, United States; Xavier University of Louisiana, New Orleans, United States; Biology, Howard College, Washington, DC, United States; Biology, Washington University in St. Louis, St. Louis, United States; Biology, Illinois Wesleyan University, Bloomington, United States; Science, Cabrini College, Radnor, United States; Biological Sciences, Carnegie Mellon University, Pittsburgh, United States; Biology, College of William and Mary, Williamsburg, United States; University of Maine, Honors College, Orono, United States; Biology, Trinity College, Hartford, United States; Morehouse College, Atlanta, United States; Xavier University of Louisiana, New Orleans, United States; Biology, University of Wisconsin-River Falls, River Falls, United States; Biological Sciences, Carnegie Mellon University, Pittsburgh, United States; Washington State University, Pullman, United States; Biology, University of California San Diego, La Jolla, United States; Biology, Gettysburg College, Gettysburg, United States; Biological Sciences, Carnegie Mellon University, Pittsburgh, United States; Biological Sciences, Lehigh University, Bethlehem, United States; Ohio State University, Columbus, United States; The Evergreen State College, Olympia, United States; Providence College, Providence, United States; Biology, Saint Joseph's University, Philadelphia, United States; Biology, Gonzaga University, Spokane, United States; Biology, Gonzaga University, Spokane, United States; University of California Santa Cruz, Santa Cruz, United States; Microbiology, Miami University, Oxford, United States; Science, Cabrini College, Radnor, United States; Biology, University of California San Diego, La Jolla, United States; Biology, College of Charleston, Charleston, United States; University of California Santa Cruz, Santa Cruz, United States; Biology, Calvin College, Grand Rapids, United States; Microbiology and Biotechnology, North Carolina State University, Raleigh, United States; Biology, Loyola Marymount University, Los Angeles, United States; Biology, Gonzaga University, Spokane, United States; Biology, Loyola Marymount University, Los Angeles, United States; Biology, North Carolina Central University, Durham, United States; Biology, Washington University in St. Louis, St. Louis, United States; Biology, Loyola Marymount University, Los Angeles, United States; Microbiology, Miami University, Oxford, United States; Biology, Washington University in St. Louis, St. Louis, United States; Biology, Hope College, Holland, United States; University of Maine, Honors College, Orono, United States; Biology, Merrimack College, North Andover, United States; Department of Microbiology, Immunology, and Molecular Genetics, University of California, Los Angeles, Los Angeles, United States; Biology, Saint Joseph's University, Philadelphia, United States; University of Florida, Gainsville, United States; University of Colorado at Boulder, Boulder, United States; Biology, University of Alabama Birmingham, Birmingham, United States; Washington State University, Pullman, United States; Ohio State University, Columbus, United States; Biological Sciences, University of Pittsburgh, Pittsburgh, United States; Biology, Washington University in St. Louis, St. Louis, United States; Purdue University, West Lafayette, United States; Science, Cabrini College, Radnor, United States; Biology, University of Texas at El Paso, El Paso, United States; Biological Sciences and Geology, Queensboro Community College, Bayside, United States; Biology, University of Texas at El Paso, El Paso, United States; Biology, University of Texas at El Paso, El Paso, United States; Biology, University of Texas at El Paso, El Paso, United States; Biology, University of Puerto Rico - Cayey, Cayey, United States; Molecular and Cell Biology Program, Oregon State University, Corvallis, United States; Microbiology and Molecular Biology, Brigham Young University, Provo, United States; Morehouse College, Atlanta, United States; The Evergreen State College, Olympia, United States; Biology, Illinois Wesleyan University, Bloomington, United States; Honors Program, Florida Gulf Coast University, Fort Myers, United States; Biology, Loyola Marymount University, Los Angeles, United States; Biology, Loyola Marymount University, Los Angeles, United States; Washington State University, Pullman, United States; Microbiology, Miami University, Oxford, United States; Biology, Calvin College, Grand Rapids, United States; Biology, Illinois Wesleyan University, Bloomington, United States; Biology, Loyola Marymount University, Los Angeles, United States; Biological Sciences, University of Pittsburgh, Pittsburgh, United States; Biology, University of Wisconsin-River Falls, River Falls, United States; Biology, Smith College, Northampton, United States; Biology, Gonzaga University, Spokane, United States; Biology, Loyola Marymount University, Los Angeles, United States; Biological Sciences, University of Pittsburgh, Pittsburgh, United States; Department of Biology, Baylor University, Waco, United States; Biological Sciences, University of North Texas, Denton, United States; Biology, Illinois Wesleyan University, Bloomington, United States; Biology, Trinity College, Hartford, United States; Biology, Howard College, Washington, DC, United States; Biology, Calvin College, Grand Rapids, United States; University of Colorado at Boulder, Boulder, United States; The Evergreen State College, Olympia, United States; Biology, James Madison University, Harrisonburg, United States; University of California Santa Cruz, Santa Cruz, United States; Biology, University of Louisiana at Monroe, Monroe, United States; Providence College, Providence, United States; Biology, Washington University in St. Louis, St. Louis, United States; Biology, Ouachita Baptist University, Arkadelphia, United States; Washington State University, Pullman, United States; Biology, Hampden-Sydney College, Farmville, United States; Microbiology, Miami University, Oxford, United States; Department of Biology, Baylor University, Waco, United States; Natural Sciences, Del Mar College, Corpus Christi, United States; Biological Sciences and Geology, Queensboro Community College, Bayside, United States; Biology and Medicine, Brown University, Providence, United States; Biology, University of Puerto Rico - Cayey, Cayey, United States; Biology, University of Puerto Rico - Cayey, Cayey, United States; Biological Sciences, University of North Texas, Denton, United States; Providence College, Providence, United States; Department of Microbiology, Immunology, and Molecular Genetics, University of California, Los Angeles, Los Angeles, United States; Montclair State University, Montclair, United States; ISBT, LaSalle University, Philadelphia, United States; Ohio State University, Columbus, United States; Biology, University of Texas at El Paso, El Paso, United States; Biology, University of Texas at El Paso, El Paso, United States; Biology, University of Puerto Rico - Cayey, Cayey, United States; Biology, University of Puerto Rico - Cayey, Cayey, United States; Biology, University of Puerto Rico - Cayey, Cayey, United States; Biology, Gonzaga University, Spokane, United States; Department of Microbiology, Immunology, and Molecular Genetics, University of California, Los Angeles, Los Angeles, United States; Biology, College of Idaho, Caldwell, United States; Providence College, Providence, United States; Biological Sciences, Lehigh University, Bethlehem, United States; Biology, Culver-Stockton College, Canton, United States; Ohio State University, Columbus, United States; Ohio State University, Columbus, United States; Biological Sciences, Lehigh University, Bethlehem, United States; Division of Natural and Health Sciences, Seton Hill University, Greensburg, United States; Biology, Illinois Wesleyan University, Bloomington, United States; Biology, Howard College, Washington, DC, United States; Western Kentucky University, Bowling Green, United States; Biological Sciences, University of Pittsburgh, Pittsburgh, United States; Biology, Washington University in St. Louis, St. Louis, United States; Biological Sciences, Lehigh University, Bethlehem, United States; Biology, University of Texas at El Paso, El Paso, United States; Biology, University of Texas at El Paso, El Paso, United States; Biology, University of Wisconsin-River Falls, River Falls, United States; Biology, Carthage College, Kenosha, United States; Biological Sciences and Geology, Queensboro Community College, Bayside, United States; Biology, Nebraska Wesleyan University, Lincoln, Nebraska, United States; Biology, University of Wisconsin-River Falls, River Falls, United States; Microbiology and Biotechnology, North Carolina State University, Raleigh, United States; Biological Sciences, University of North Texas, Denton, United States; Biology, Culver-Stockton College, Canton, United States; School of Science and Technology, Georgia Gwinnett College, Lawrenceville, United States; The Evergreen State College, Olympia, United States; University of California Santa Cruz, Santa Cruz, United States; Biology, College of William and Mary, Williamsburg, United States; University of Colorado at Boulder, Boulder, United States; Biology, Spelman College, Atlanta, United States; Ohio State University, Columbus, United States; Environmental and Biological Science, University of Maine, Machias, Machias, United States; Biology, Gonzaga University, Spokane, United States; Natural Sciences, University of Houston-Downtown, Houston, United States; Biology, Jacksonville State University, Jacksonville, United States; Department of Biology, Baylor University, Waco, United States; Biology, Trinity College, Hartford, United States; Biology, University of Texas at El Paso, El Paso, United States; Biology, North Carolina Central University, Durham, United States; Biology, University of Louisiana at Monroe, Monroe, United States; Biology, Gonzaga University, Spokane, United States; Microbiology and Molecular Biology, Brigham Young University, Provo, United States; University of Maine, Honors College, Orono, United States; Biology, University of Wisconsin-River Falls, River Falls, United States; Biological Sciences, University of Pittsburgh, Pittsburgh, United States; Microbiology and Biotechnology, North Carolina State University, Raleigh, United States; University of California Santa Cruz, Santa Cruz, United States; Biology, Trinity College, Hartford, United States; Marine Science, Southern Maine Community College, South Portland, United States; Biology, Loyola Marymount University, Los Angeles, United States; Biological Sciences, Carnegie Mellon University, Pittsburgh, United States; Biological Sciences, Carnegie Mellon University, Pittsburgh, United States; Biology, University of Texas at El Paso, El Paso, United States; Biology, Howard College, Washington, DC, United States; University of Colorado at Boulder, Boulder, United States; Biology, Gonzaga University, Spokane, United States; Xavier University of Louisiana, New Orleans, United States; Xavier University of Louisiana, New Orleans, United States; Biology, University of California San Diego, La Jolla, United States; Biology, University of Alabama Birmingham, Birmingham, United States; Biological Sciences, Carnegie Mellon University, Pittsburgh, United States; Ohio State University, Columbus, United States; Biology, Washington University in St. Louis, St. Louis, United States; Biology, University of California San Diego, La Jolla, United States; Biology, CUNY, Queens College, Queens, United States; Biology, Washington University in St. Louis, St. Louis, United States; Biology, Howard College, Washington, DC, United States; Biology, University of Texas at El Paso, El Paso, United States; Biology, University of Texas at El Paso, El Paso, United States; Biology, University of Texas at El Paso, El Paso, United States; Department of Microbiology, Immunology, and Molecular Genetics, University of California, Los Angeles, Los Angeles, United States; Purdue University, West Lafayette, United States; Biology, Culver-Stockton College, Canton, United States; Biology and Chemistry, Nyack College, Nyack, United States; Biology and Medicine, Brown University, Providence, United States; Biology, College of St. Scholastica, Duluth, United States; Department of Biology, Baylor University, Waco, United States; Biological Sciences, University of North Texas, Denton, United States; Biology, College of St. Scholastica, Duluth, United States; Western Kentucky University, Bowling Green, United States; Biology, Culver-Stockton College, Canton, United States; Biology, College of William and Mary, Williamsburg, United States; Biology, Washington University in St. Louis, St. Louis, United States; Biology, Washington University in St. Louis, St. Louis, United States; Virginia Commonwealth University, Richmond, United States; The Evergreen State College, Olympia, United States; Biology, University of Texas at El Paso, El Paso, United States; Department of Microbiology, Immunology, and Molecular Genetics, University of California, Los Angeles, Los Angeles, United States; Biology, Culver-Stockton College, Canton, United States; Biological Sciences, Carnegie Mellon University, Pittsburgh, United States; Biology, College of Charleston, Charleston, United States; School of Science and Technology, Georgia Gwinnett College, Lawrenceville, United States; Biology, College of Idaho, Caldwell, United States; Biology, College of Charleston, Charleston, United States; Microbiology and Molecular Biology, Brigham Young University, Provo, United States; Ohio State University, Columbus, United States; University of California Santa Cruz, Santa Cruz, United States; Biology, Spelman College, Atlanta, United States; Biology, Spelman College, Atlanta, United States; Biology, Spelman College, Atlanta, United States; University of Colorado at Boulder, Boulder, United States; Biology, University of Louisiana at Monroe, Monroe, United States; Purdue University, West Lafayette, United States; Biology, University of Louisiana at Monroe, Monroe, United States; Biology, Loyola Marymount University, Los Angeles, United States; Biology, University of Texas at El Paso, El Paso, United States; Biology, North Carolina Central University, Durham, United States; Biology and Chemistry, Nyack College, Nyack, United States; Biology, Calvin College, Grand Rapids, United States; Biology, Gettysburg College, Gettysburg, United States; Biology, College of William and Mary, Williamsburg, United States; University of Maine, Honors College, Orono, United States; Biology, Saint Joseph's University, Philadelphia, United States; Biological Sciences, Lehigh University, Bethlehem, United States; Science, Cabrini College, Radnor, United States; University of Maine, Honors College, Orono, United States; Biology, University of Texas at El Paso, El Paso, United States; University of California Santa Cruz, Santa Cruz, United States; Biology, Jacksonville State University, Jacksonville, United States; Biology, Howard College, Washington, DC, United States; Biology, College of William and Mary, Williamsburg, United States; Biology, College of William and Mary, Williamsburg, United States; Biology, Gonzaga University, Spokane, United States; University of Maine, Honors College, Orono, United States; Biological Sciences, University of Pittsburgh, Pittsburgh, United States; Microbiology and Molecular Biology, Brigham Young University, Provo, United States; University of California Santa Cruz, Santa Cruz, United States; Biology, Illinois Wesleyan University, Bloomington, United States; Natural Sciences, Del Mar College, Corpus Christi, United States; Microbiology, Miami University, Oxford, United States; Morehouse College, Atlanta, United States; Biology, College of Charleston, Charleston, United States; Biology, University of Louisiana at Monroe, Monroe, United States; Western Kentucky University, Bowling Green, United States; Biology, Gettysburg College, Gettysburg, United States; Biology, Gonzaga University, Spokane, United States; Biology, University of Louisiana at Monroe, Monroe, United States; School of Science and Technology, Georgia Gwinnett College, Lawrenceville, United States; Biology, Howard College, Washington, DC, United States; Southern Connecticut State University, New Haven, United States; Biology, University of Louisiana at Monroe, Monroe, United States; Biology, Washington University in St. Louis, St. Louis, United States; Biology, Washington University in St. Louis, St. Louis, United States; Biology, University of California San Diego, La Jolla, United States; Biological Sciences, Carnegie Mellon University, Pittsburgh, United States; Biological Sciences, Carnegie Mellon University, Pittsburgh, United States; Biology, Gettysburg College, Gettysburg, United States; Biological Sciences, Lehigh University, Bethlehem, United States; Biology, Hope College, Holland, United States; Environmental and Biological Science, University of Maine, Machias, Machias, United States; Department of Biology, Baylor University, Waco, United States; Ohio State University, Columbus, United States; Biology, University of Wisconsin-River Falls, River Falls, United States; Washington State University, Pullman, United States; Department of Biology, Baylor University, Waco, United States; Microbiology and Biotechnology, North Carolina State University, Raleigh, United States; Biology, Gonzaga University, Spokane, United States; Microbiology, Miami University, Oxford, United States; Biology and Chemistry, Nyack College, Nyack, United States; Biology, Hope College, Holland, United States; Biological Sciences and Geology, Queensboro Community College, Bayside, United States; Biology, Gonzaga University, Spokane, United States; Chemistry, Montana Tech of the University of Montana, Butte, United States; Biology, University of Texas at El Paso, El Paso, United States; Biology and Medicine, Brown University, Providence, United States; Biology, North Carolina Central University, Durham, United States; Department of Biology, Baylor University, Waco, United States; Biology, Calvin College, Grand Rapids, United States; Biology, Jacksonville State University, Jacksonville, United States; Montclair State University, Montclair, United States; Microbiology and Biotechnology, North Carolina State University, Raleigh, United States; Biology, University of California San Diego, La Jolla, United States; University of Colorado at Boulder, Boulder, United States; Biology, University of Texas at El Paso, El Paso, United States; Biology, College of Charleston, Charleston, United States; Biology, Loyola Marymount University, Los Angeles, United States; Biology, Gonzaga University, Spokane, United States; Biology, College of Idaho, Caldwell, United States; Biology, Smith College, Northampton, United States; Biology, Hope College, Holland, United States; Biology, Hope College, Holland, United States; University of Colorado at Boulder, Boulder, United States; University of California Santa Cruz, Santa Cruz, United States; Biology, University of Louisiana at Monroe, Monroe, United States; Biological Sciences, Carnegie Mellon University, Pittsburgh, United States; Biology, Illinois Wesleyan University, Bloomington, United States; Biology, Gonzaga University, Spokane, United States; Biology, Hope College, Holland, United States; Biology, Howard College, Washington, DC, United States; Morehouse College, Atlanta, United States; Biology, North Carolina Central University, Durham, United States; School of Science and Technology, Georgia Gwinnett College, Lawrenceville, United States; School of Science and Technology, Georgia Gwinnett College, Lawrenceville, United States; Biology, Gettysburg College, Gettysburg, United States; University of California Santa Cruz, Santa Cruz, United States; University of California Santa Cruz, Santa Cruz, United States; Microbiology, Miami University, Oxford, United States; Biology, Calvin College, Grand Rapids, United States; University of California Santa Cruz, Santa Cruz, United States; Biology, University of California San Diego, La Jolla, United States; Environmental and Biological Science, University of Maine, Machias, Machias, United States; Biology, Carthage College, Kenosha, United States; Division of Natural and Health Sciences, Seton Hill University, Greensburg, United States; Department of Biology, Baylor University, Waco, United States; Biology, College of Charleston, Charleston, United States; Biology, Washington University in St. Louis, St. Louis, United States; Biology, Washington University in St. Louis, St. Louis, United States; University of California Santa Cruz, Santa Cruz, United States; Biology, Washington University in St. Louis, St. Louis, United States; The Evergreen State College, Olympia, United States; Biology, University of Louisiana at Monroe, Monroe, United States; Biology, University of Louisiana at Monroe, Monroe, United States; Biological Sciences, University of North Texas, Denton, United States; Biology, Calvin College, Grand Rapids, United States; Purdue University, West Lafayette, United States; Biology, Howard College, Washington, DC, United States; Biology, Gonzaga University, Spokane, United States; Providence College, Providence, United States; Biology, Howard College, Washington, DC, United States; Southern Connecticut State University, New Haven, United States; Microbiology and Biotechnology, North Carolina State University, Raleigh, United States; Biological Sciences, University of North Texas, Denton, United States; Molecular and Biomedical Sciences, University of Maine, Honors College, Orono, United States; Biology, Gonzaga University, Spokane, United States; University of Colorado at Boulder, Boulder, United States; Biology, Smith College, Northampton, United States; Biology, University of California San Diego, La Jolla, United States; Western Kentucky University, Bowling Green, United States; University of Florida, Gainsville, United States; Biology, Gonzaga University, Spokane, United States; Montclair State University, Montclair, United States; Biology, University of Puerto Rico - Cayey, Cayey, United States; University of California Santa Cruz, Santa Cruz, United States; School of Science and Technology, Georgia Gwinnett College, Lawrenceville, United States; Biology, Gonzaga University, Spokane, United States; University of Maine, Honors College, Orono, United States; Biology, College of Charleston, Charleston, United States; Department of Microbiology, Immunology, and Molecular Genetics, University of California, Los Angeles, Los Angeles, United States; Department of Biology, Baylor University, Waco, United States; Biology, College of St. Scholastica, Duluth, United States; Biology, University of California San Diego, La Jolla, United States; Department of Microbiology, Immunology, and Molecular Genetics, University of California, Los Angeles, Los Angeles, United States; Xavier University of Louisiana, New Orleans, United States; Biology, Saint Joseph's University, Philadelphia, United States; Biological Sciences, Florida Gulf Coast University, Fort Myers, United States; Biology, Howard College, Washington, DC, United States; Microbiology and Molecular Biology, Brigham Young University, Provo, United States; University of Florida, Gainsville, United States; Biological Sciences, University of Pittsburgh, Pittsburgh, United States; Biology, Howard College, Washington, DC, United States; Morehouse College, Atlanta, United States; Western Kentucky University, Bowling Green, United States; Biology, University of Louisiana at Monroe, Monroe, United States; Biology, Howard College, Washington, DC, United States; Biology, Washington University in St. Louis, St. Louis, United States; Biological Sciences, University of North Texas, Denton, United States; Biology, University of California San Diego, La Jolla, United States; Biology, Ouachita Baptist University, Arkadelphia, United States; Physical and Life sciences, Chadron State College, Chadron, United States; Providence College, Providence, United States; Biology, Carthage College, Kenosha, United States; Biology, Gettysburg College, Gettysburg, United States; Microbiology, Miami University, Oxford, United States; Biological Sciences, Carnegie Mellon University, Pittsburgh, United States; Biological Sciences, Carnegie Mellon University, Pittsburgh, United States; Biology, Washington University in St. Louis, St. Louis, United States; Biology, Washington University in St. Louis, St. Louis, United States; Biological Sciences, Carnegie Mellon University, Pittsburgh, United States; Microbiology, Miami University, Oxford, United States; Biology, Howard College, Washington, DC, United States; Biology, College of William and Mary, Williamsburg, United States; Microbiology and Biotechnology, North Carolina State University, Raleigh, United States; Biology, Loyola Marymount University, Los Angeles, United States; Biology, Calvin College, Grand Rapids, United States; Biology, College of Charleston, Charleston, United States; Honors Program, Florida Gulf Coast University, Fort Myers, United States; Biology, Saint Joseph's University, Philadelphia, United States; Biology, College of St. Scholastica, Duluth, United States; Biology, Gonzaga University, Spokane, United States; Science, Cabrini College, Radnor, United States; University of California Santa Cruz, Santa Cruz, United States; Department of Microbiology, Immunology, and Molecular Genetics, University of California, Los Angeles, Los Angeles, United States; Biological Sciences, Carnegie Mellon University, Pittsburgh, United States; Biological Sciences and Geology, Queensboro Community College, Bayside, United States; Center for the Study of Biological Complexity, Virginia Commonwealth University, Richmond, United States; Biology, College of St. Scholastica, Duluth, United States; Biology, Washington University in St. Louis, St. Louis, United States; Biology, Jacksonville State University, Jacksonville, United States; The Evergreen State College, Olympia, United States; Morehouse College, Atlanta, United States; Xavier University of Louisiana, New Orleans, United States; Biology, Saint Joseph's University, Philadelphia, United States; The Evergreen State College, Olympia, United States; Biology, Calvin College, Grand Rapids, United States; Biology, Culver-Stockton College, Canton, United States; Biology, Hope College, Holland, United States; Western Kentucky University, Bowling Green, United States; Biology, Nebraska Wesleyan University, Lincoln, Nebraska, United States; Biology, Loyola Marymount University, Los Angeles, United States; Biology, Culver-Stockton College, Canton, United States; Biology, University of Alabama Birmingham, Birmingham, United States; Biological Sciences, University of North Texas, Denton, United States; Biology, University of Louisiana at Monroe, Monroe, United States; Biology, Gonzaga University, Spokane, United States; Biology, Illinois Wesleyan University, Bloomington, United States; Biology, Howard College, Washington, DC, United States; Western Kentucky University, Bowling Green, United States; Washington State University, Pullman, United States; Biology, Gonzaga University, Spokane, United States; ISBT, LaSalle University, Philadelphia, United States; Biology, University of Louisiana at Monroe, Monroe, United States; Providence College, Providence, United States; Western Kentucky University, Bowling Green, United States; Western Kentucky University, Bowling Green, United States; Biology, Gonzaga University, Spokane, United States; Biological Sciences, University of North Texas, Denton, United States; Biological Sciences and Geology, Queensboro Community College, Bayside, United States; Biology, Gonzaga University, Spokane, United States; Biology, University of Puerto Rico - Cayey, Cayey, United States; Biological Sciences, Lehigh University, Bethlehem, United States; The Evergreen State College, Olympia, United States; Biology, Calvin College, Grand Rapids, United States; Science, Cabrini College, Radnor, United States; Biology, Washington University in St. Louis, St. Louis, United States; Biology, Saint Joseph's University, Philadelphia, United States; Biology, Saint Joseph's University, Philadelphia, United States; Biological Sciences, Carnegie Mellon University, Pittsburgh, United States; Biological Sciences, University of North Texas, Denton, United States; Biology, Trinity College, Hartford, United States; Biology, University of Texas at El Paso, El Paso, United States; Biology, Nebraska Wesleyan University, Lincoln, Nebraska, United States; Ohio State University, Columbus, United States; Biology, Calvin College, Grand Rapids, United States; Biology, University of California San Diego, La Jolla, United States; Biological Sciences and Geology, Queensboro Community College, Bayside, United States; The Evergreen State College, Olympia, United States; Biological Sciences, University of North Texas, Denton, United States; Virginia Commonwealth University, Richmond, United States; Biology, University of California San Diego, La Jolla, United States; Biology, Loyola Marymount University, Los Angeles, United States; Biology, Gonzaga University, Spokane, United States; University of Colorado at Boulder, Boulder, United States; University of Florida, Gainsville, United States; Virginia Commonwealth University, Richmond, United States; Biology, Washington University in St. Louis, St. Louis, United States; Montclair State University, Montclair, United States; Biology, University of Louisiana at Monroe, Monroe, United States; University of California Santa Cruz, Santa Cruz, United States; Environmental and Biological Science, University of Maine, Machias, Machias, United States; Ohio State University, Columbus, United States; Biology, James Madison University, Harrisonburg, United States; University of Florida, Gainsville, United States; Department of Microbiology, Immunology, and Molecular Genetics, University of California, Los Angeles, Los Angeles, United States; University of Colorado at Boulder, Boulder, United States; Purdue University, West Lafayette, United States; Western Kentucky University, Bowling Green, United States; Biological Sciences, Lehigh University, Bethlehem, United States; University of California Santa Cruz, Santa Cruz, United States; ISBT, LaSalle University, Philadelphia, United States; Ohio State University, Columbus, United States; Biology, Trinity College, Hartford, United States; Washington State University, Pullman, United States; School of Science and Technology, Georgia Gwinnett College, Lawrenceville, United States; Xavier University of Louisiana, New Orleans, United States; Biology, University of Alabama Birmingham, Birmingham, United States; Biology, Illinois Wesleyan University, Bloomington, United States; Biology, University of Wisconsin-River Falls, River Falls, United States; Microbiology and Biotechnology, North Carolina State University, Raleigh, United States; The Evergreen State College, Olympia, United States; Department of Biological Sciences, University of Maryland, Baltimore County, Baltimore, United States; Biological Sciences, Lehigh University, Bethlehem, United States; Biology, Loyola Marymount University, Los Angeles, United States; Biological Sciences, Lehigh University, Bethlehem, United States; Biology, North Carolina Central University, Durham, United States; Biology, University of Alabama Birmingham, Birmingham, United States; Biology, Washington University in St. Louis, St. Louis, United States; Biology, Calvin College, Grand Rapids, United States; Biology and Chemistry, Nyack College, Nyack, United States; Biology, Loyola Marymount University, Los Angeles, United States; Biology, University of Louisiana at Monroe, Monroe, United States; Biology, Saint Joseph's University, Philadelphia, United States; Biology, Culver-Stockton College, Canton, United States; School of Science and Technology, Georgia Gwinnett College, Lawrenceville, United States; Department of Microbiology, Immunology, and Molecular Genetics, University of California, Los Angeles, Los Angeles, United States; University of Colorado at Boulder, Boulder, United States; Montclair State University, Montclair, United States; Biology, University of California San Diego, La Jolla, United States; Biology, Washington University in St. Louis, St. Louis, United States; Western Kentucky University, Bowling Green, United States; Biology, Smith College, Northampton, United States; Biology, Gettysburg College, Gettysburg, United States; Virginia Commonwealth University, Richmond, United States; Biology, Trinity College, Hartford, United States; Biology, Gettysburg College, Gettysburg, United States; Department of Biology, Baylor University, Waco, United States; Biology, Illinois Wesleyan University, Bloomington, United States; Biological Sciences, Carnegie Mellon University, Pittsburgh, United States; Biological Sciences, Lehigh University, Bethlehem, United States; Biological Sciences, Carnegie Mellon University, Pittsburgh, United States; Biology and Chemistry, Nyack College, Nyack, United States; ISBT, LaSalle University, Philadelphia, United States; Biology, Trinity College, Hartford, United States; Virginia Commonwealth University, Richmond, United States; Biology, College of Idaho, Caldwell, United States; Western Kentucky University, Bowling Green, United States; Biology, Ouachita Baptist University, Arkadelphia, United States; Biology, University of Louisiana at Monroe, Monroe, United States; Western Kentucky University, Bowling Green, United States; Biology, Saint Joseph's University, Philadelphia, United States; Southern Connecticut State University, New Haven, United States; Biology, Carthage College, Kenosha, United States; University of Florida, Gainsville, United States; The Evergreen State College, Olympia, United States; Virginia Commonwealth University, Richmond, United States; Ohio State University, Columbus, United States; Biology, Trinity College, Hartford, United States; Biology, Carthage College, Kenosha, United States; Ohio State University, Columbus, United States; Microbiology, Miami University, Oxford, United States; Biology and Chemistry, Nyack College, Nyack, United States; Purdue University, West Lafayette, United States; Natural Sciences, University of Houston-Downtown, Houston, United States; Biology, College of St. Scholastica, Duluth, United States; Biology, University of Wisconsin-River Falls, River Falls, United States; Biological Sciences, University of North Texas, Denton, United States; Microbiology and Biotechnology, North Carolina State University, Raleigh, United States; Biology, Hope College, Holland, United States; Biology, Trinity College, Hartford, United States; Biology, University of Wisconsin-River Falls, River Falls, United States; Biology, Washington University in St. Louis, St. Louis, United States; Department of Microbiology, Immunology, and Molecular Genetics, University of California, Los Angeles, Los Angeles, United States; Biology, Gonzaga University, Spokane, United States; Biology, College of Idaho, Caldwell, United States; Biology, Washington University in St. Louis, St. Louis, United States; Microbiology and Biotechnology, North Carolina State University, Raleigh, United States; University of Colorado at Boulder, Boulder, United States; Department of Biology, Baylor University, Waco, United States; Biology, College of Idaho, Caldwell, United States; Biological Sciences, University of North Texas, Denton, United States; Biology, Gonzaga University, Spokane, United States; Biology, Ouachita Baptist University, Arkadelphia, United States; University of Colorado at Boulder, Boulder, United States; Biology, College of Charleston, Charleston, United States; Biology, Gettysburg College, Gettysburg, United States; Biology, Illinois Wesleyan University, Bloomington, United States; Biology, Gonzaga University, Spokane, United States; Biology, University of Wisconsin-River Falls, River Falls, United States; Biology, University of Wisconsin-River Falls, River Falls, United States; Biological Sciences, Lehigh University, Bethlehem, United States; Biological Sciences, University of Pittsburgh, Pittsburgh, United States; Biology, Smith College, Northampton, United States; Biology, Gonzaga University, Spokane, United States; Biology, Gonzaga University, Spokane, United States; Biology, Washington University in St. Louis, St. Louis, United States; Biology, Illinois Wesleyan University, Bloomington, United States; Purdue University, West Lafayette, United States; Biology, University of Wisconsin-River Falls, River Falls, United States; Biology, College of St. Scholastica, Duluth, United States; University of California Santa Cruz, Santa Cruz, United States; Biology, Carthage College, Kenosha, United States; Biology, Washington University in St. Louis, St. Louis, United States; Biology, Gettysburg College, Gettysburg, United States; Biology, Loyola Marymount University, Los Angeles, United States; Biology, University of California San Diego, La Jolla, United States; Department of Biology, Baylor University, Waco, United States; Biology, Ouachita Baptist University, Arkadelphia, United States; Biology, Loyola Marymount University, Los Angeles, United States; Biology, University of Alabama Birmingham, Birmingham, United States; Biology, University of Louisiana at Monroe, Monroe, United States; Department of Microbiology, Immunology, and Molecular Genetics, University of California, Los Angeles, Los Angeles, United States; University of Maine, Honors College, Orono, United States; Biology, University of California San Diego, La Jolla, United States; Biology, Trinity College, Hartford, United States; Biology, University of Puerto Rico - Cayey, Cayey, United States; Biology, Culver-Stockton College, Canton, United States; University of Maine, Honors College, Orono, United States; Purdue University, West Lafayette, United States; Biology, Saint Joseph's University, Philadelphia, United States; Biological Sciences, Lehigh University, Bethlehem, United States; Department of Microbiology, Immunology, and Molecular Genetics, University of California, Los Angeles, Los Angeles, United States; Biology, Wilkes University, Wilkes-Barre, United States; Biology, Gonzaga University, Spokane, United States; University of Maine, Honors College, Orono, United States; Purdue University, West Lafayette, United States; Biology, University of California San Diego, La Jolla, United States; Biology, Trinity College, Hartford, United States; University of Florida, Gainsville, United States; Biological Sciences, Lehigh University, Bethlehem, United States; Biology, College of St. Scholastica, Duluth, United States; Biology, College of Idaho, Caldwell, United States; Biology, University of California San Diego, La Jolla, United States; Biology, Gettysburg College, Gettysburg, United States; Honors Program, Florida Gulf Coast University, Fort Myers, United States; Biology, Illinois Wesleyan University, Bloomington, United States; Biology, Calvin College, Grand Rapids, United States; The Evergreen State College, Olympia, United States; Biology, Merrimack College, North Andover, United States; Biology, Carthage College, Kenosha, United States; Biological Sciences, University of Pittsburgh, Pittsburgh, United States; Biology, Gonzaga University, Spokane, United States; Biology, Gonzaga University, Spokane, United States; Natural Sciences, Del Mar College, Corpus Christi, United States; Biological Sciences, Lehigh University, Bethlehem, United States; University of Florida, Gainsville, United States; Biology, Howard College, Washington, DC, United States; Washington State University, Pullman, United States; Biology, Trinity College, Hartford, United States; Montclair State University, Montclair, United States; Biology, Loyola Marymount University, Los Angeles, United States; Washington State University, Pullman, United States; Biology, University of Louisiana at Monroe, Monroe, United States; Biology, University of Alabama Birmingham, Birmingham, United States; Biology, University of Texas at El Paso, El Paso, United States; Department of Microbiology, Immunology, and Molecular Genetics, University of California, Los Angeles, Los Angeles, United States; Biology and Medicine, Brown University, Providence, United States; University of Florida, Gainsville, United States; Providence College, Providence, United States; Biology, Merrimack College, North Andover, United States; Biology, Gonzaga University, Spokane, United States; University of Colorado at Boulder, Boulder, United States; Biological Sciences, Carnegie Mellon University, Pittsburgh, United States; Biology, University of Alabama Birmingham, Birmingham, United States; Biological Sciences, Carnegie Mellon University, Pittsburgh, United States; Department of Biology, Baylor University, Waco, United States; Biology, Washington University in St. Louis, St. Louis, United States; Biology, Saint Joseph's University, Philadelphia, United States; Biology, North Carolina Central University, Durham, United States; Western Kentucky University, Bowling Green, United States; Biology, College of Charleston, Charleston, United States; Biology, Jacksonville State University, Jacksonville, United States; Biology, Jacksonville State University, Jacksonville, United States; Department of Microbiology, Immunology, and Molecular Genetics, University of California, Los Angeles, Los Angeles, United States; Biology, Hope College, Holland, United States; Department of Biology, Baylor University, Waco, United States; Biology, Gettysburg College, Gettysburg, United States; Biology, North Carolina Central University, Durham, United States; Microbiology, Miami University, Oxford, United States; Biology, Gettysburg College, Gettysburg, United States; Biology, Montana Tech of the University of Montana, Butte, United States; Biology, Gonzaga University, Spokane, United States; Biology, Spelman College, Atlanta, United States; Western Kentucky University, Bowling Green, United States; Biology, Howard College, Washington, DC, United States; Biological Sciences, Lehigh University, Bethlehem, United States; Biological Sciences, University of Mary Washington, Fredericksburg, United States; Biology, Jacksonville State University, Jacksonville, United States; Biology, Southern Connecticut State University, New Haven, United States; Department of Microbiology, Immunology, and Molecular Genetics, University of California, Los Angeles, Los Angeles, United States; Biology, University of California San Diego, La Jolla, United States; University of Colorado at Boulder, Boulder, United States; Ohio State University, Columbus, United States; Purdue University, West Lafayette, United States; Biological Sciences, Carnegie Mellon University, Pittsburgh, United States; Biology, Washington University in St. Louis, St. Louis, United States; Master of Physician Assistant Studies, Indiana Universiy, Indianapolis, United States; Biological Sciences, Carnegie Mellon University, Pittsburgh, United States; Providence College, Providence, United States; Montclair State University, Montclair, United States; Biology, College of Charleston, Charleston, United States; University of California Santa Cruz, Santa Cruz, United States; Microbiology, Miami University, Oxford, United States; Biology, Calvin College, Grand Rapids, United States; Biology, Washington University in St. Louis, St. Louis, United States; Biology, Montana Tech of the University of Montana, Butte, United States; Western Kentucky University, Bowling Green, United States; Purdue University, West Lafayette, United States; University of Maine, Honors College, Orono, United States; Biology, Washington University in St. Louis, St. Louis, United States; Biology, University of Alabama Birmingham, Birmingham, United States; The Evergreen State College, Olympia, United States; Biology, University of Texas at El Paso, El Paso, United States; University of Colorado at Boulder, Boulder, United States; Biology, University of Puerto Rico - Cayey, Cayey, United States; Ohio State University, Columbus, United States; Biology, University of Alabama Birmingham, Birmingham, United States; Biology, Loyola Marymount University, Los Angeles, United States; Biological Sciences, University of Mary Washington, Fredericksburg, United States; Biology, Saint Joseph's University, Philadelphia, United States; Morehouse College, Atlanta, United States; Ohio State University, Columbus, United States; Microbiology, Howard College, Washington, DC, United States; Biology, Saint Joseph's University, Philadelphia, United States; Pathology, University of Alabama Birmingham, Birmingham, United States; Biology, College of Charleston, Charleston, United States; Biology, University of Alabama Birmingham, Birmingham, United States; Biological Sciences, University of Pittsburgh, Pittsburgh, United States; Biology, University of Texas at El Paso, El Paso, United States; Biological Sciences, Carnegie Mellon University, Pittsburgh, United States; Natural Sciences, Del Mar College, Corpus Christi, United States; Biology, University of Puerto Rico - Cayey, Cayey, United States; Department of Microbiology, Immunology, and Molecular Genetics, University of California, Los Angeles, Los Angeles, United States; University of California Santa Cruz, Santa Cruz, United States; Biology, Washington University in St. Louis, St. Louis, United States; Biology, Hope College, Holland, United States; Biology, University of Louisiana at Monroe, Monroe, United States; Biology, Gettysburg College, Gettysburg, United States; Virginia Commonwealth University, Richmond, United States; University of Colorado at Boulder, Boulder, United States; Biology, University of Wisconsin-River Falls, River Falls, United States; Biological Sciences and Geology, Queensboro Community College, Bayside, United States; Biology, Washington University in St. Louis, St. Louis, United States; Biology, University of California San Diego, La Jolla, United States; Biology, University of Texas at El Paso, El Paso, United States; Division of Natural and Health Sciences, Seton Hill University, Greensburg, United States; Biology, Washington University in St. Louis, St. Louis, United States; Biology, University of California San Diego, La Jolla, United States; Department of Microbiology, Immunology, and Molecular Genetics, University of California, Los Angeles, Los Angeles, United States; Biology, Hope College, Holland, United States; Microbiology and Molecular Biology, Brigham Young University, Provo, United States; Purdue University, West Lafayette, United States; Biology, Saint Joseph's University, Philadelphia, United States; Biology Department, Georgia State University, Milledgeville, United States; Biology, North Carolina Central University, Durham, United States; Health Sciences, James Madison University, Harrisonburg, United States; Biological Sciences, Lehigh University, Bethlehem, United States; Biology, Gonzaga University, Spokane, United States; ISBT, LaSalle University, Philadelphia, United States; Biology, Saint Joseph's University, Philadelphia, United States; Biology, University of Puerto Rico - Cayey, Cayey, United States; Biology, Carthage College, Kenosha, United States; Biology, College of Charleston, Charleston, United States; Biological Sciences, Lehigh University, Bethlehem, United States; Biology, Gonzaga University, Spokane, United States; Physical and Life Sciences, Chadron State College, Chadron, United States; Biology, University of Texas at El Paso, El Paso, United States; Biology, University of California San Diego, La Jolla, United States; Biology, Loyola Marymount University, Los Angeles, United States; Biological Sciences, Lehigh University and Cabrini College, Bethlehem, United States; Biological Sciences, Lehigh University, Bethlehem, United States; Montclair State University, Montclair, United States; Biology, University of Puerto Rico - Cayey, Cayey, United States; Biology, University of Puerto Rico - Cayey, Cayey, United States; Biology and Chemistry, Nyack College, Nyack, United States; Department of Biology, Baylor University, Waco, United States; Biological Sciences, University of North Texas, Denton, United States; Biology, Howard College, Washington, DC, United States; Montclair State University, Montclair, United States; Biology, Washington University in St. Louis, St. Louis, United States; Biology, University of Alabama Birmingham, Birmingham, United States; Honors Program, Florida Gulf Coast University, Fort Myers, United States; Biology, Nebraska Wesleyan University, Lincoln, Nebraska, United States; Biology, Illinois Wesleyan University, Bloomington, United States; Biological Sciences and Geology, Queensboro Community College, Bayside, United States; Natural Sciences, Del Mar College, Corpus Christi, United States; Biology, Howard College, Washington, DC, United States; The Evergreen State College, Olympia, United States; Biology, University of Texas at El Paso, El Paso, United States; Marine Science, Southern Maine Community College, South Portland, United States; Biology, College of St. Scholastica, Duluth, United States; Biology, Culver-Stockton College, Canton, United States; Biology, Howard College, Washington, DC, United States; University of Colorado at Boulder, Boulder, United States; Ohio State University, Columbus, United States; Biology, University of Texas at El Paso, El Paso, United States; Virginia Commonwealth University, Richmond, United States; Biology, Calvin College, Grand Rapids, United States; Biology, Saint Joseph's University, Philadelphia, United States; Biology, Saint Joseph's University, Philadelphia, United States; Biological Sciences, Carnegie Mellon University, Pittsburgh, United States; Biological Sciences, Lehigh University, Bethlehem, United States; Purdue University, West Lafayette, United States; Division of Natural and Health Sciences, Seton Hill University, Greensburg, United States; Biology, Carthage College, Kenosha, United States; The Evergreen State College, Olympia, United States; Biology, Gonzaga University, Spokane, United States; Department of Biology, Baylor University, Waco, United States; Biology, Gonzaga University, Spokane, United States; Biology, University of California San Diego, La Jolla, United States; Biology, Hope College, Holland, United States; Biology, Loyola Marymount University, Los Angeles, United States; Biology, Gonzaga University, Spokane, United States; Biology, Saint Joseph's University, Philadelphia, United States; Biology, University of Alabama Birmingham, Birmingham, United States; Microbiology and Biotechnology, North Carolina State University, Raleigh, United States; Microbiology and Biotechnology, North Carolina State University, Raleigh, United States; Biology, Trinity College, Hartford, United States; Biology, University of California San Diego, La Jolla, United States; Biology, Trinity College, Hartford, United States; Biology, University of California San Diego, La Jolla, United States; Xavier University of Louisiana, New OrleansUnited States; Microbiology and Biotechnology, North Carolina State University, Raleigh, United States; Biology, Montana Tech of the University of Montana, Butte, United States; Biology, University of Louisiana at Monroe, Monroe, United States; Microbiology, Miami University, Oxford, United States; Biology, Howard College, Washington, DC, United States; Morehouse College, Atlanta, United States; Morehouse College, Atlanta, United States; Biology, Howard College, Washington, DC, United States; Purdue University, West Lafayette, United States; The Evergreen State College, Olympia, United States; Biology, University of Alabama Birmingham, Birmingham, United States; Environmental and Biological Science, University of Maine, Machias, Machias, United States; Biology, Nebraska Wesleyan University, Lincoln, Nebraska, United States; Biology, College of Charleston, Charleston, United States; Microbiology, Miami University, Oxford, United States; Biology, Nebraska Wesleyan University, Lincoln, Nebraska, United States; School of Science and Technology, Georgia Gwinnett College, Lawrenceville, United States; Biology, Hope College, Holland, United States; Honors Program, Florida Gulf Coast University, Fort Myers, United States; Biology, University of Alabama Birmingham, Birmingham, United States; Biology, Trinity College, Hartford, United States; Ohio State University, Columbus, United States; Biological Sciences, University of Pittsburgh, Pittsburgh, United States; Biology, College of Charleston, Charleston, United States; Biology, North Carolina Central University, Durham, United States; Biology, Loyola Marymount University, Los Angeles, United States; Biological Sciences, Carnegie Mellon University, Pittsburgh, United States; Biology, University of Puerto Rico - Cayey, Cayey, United States; Biology, Loyola Marymount University, Los Angeles, United States; Department of Biology, Baylor University, Waco, United States; Biology, Jacksonville State University, Jacksonville, United States; Ohio State University, Columbus, United States; Department of Biological Sciences, Genetics Course - Univeristy of Pittsburgh, Pittsburgh, United States; Biology, Calvin College, Grand Rapids, United States; Biology, Gonzaga University, Spokane, United States; Biology, University of Puerto Rico - Cayey, Cayey, United States; Molecular and Cell Biology Program, Oregon State University, Corvallis, United States; Natural Sciences, University of Houston-Downtown, Houston, United States; University of Florida, Gainsville, United States; Biology, Gettysburg College, Gettysburg, United States; Biology, University of Alabama Birmingham, Birmingham, United States; Biology, Hope College, Holland, United States; Montclair State University, Montclair, United States; Biological Sciences and Geology, Queensboro Community College, Bayside, United States; Biological Sciences, University of North Texas, Denton, United States; Microbiology and Molecular Biology, Brigham Young University, Provo, United States; Biology, Hope College, Holland, United States; Biology, Smith College, Northampton, United States; Montclair State University, Montclair, United States; Montclair State University, Montclair, United States; Microbiology, Miami University, Oxford, United States; Purdue University, West Lafayette, United States; Biology, Hope College, Holland, United States; Department of Microbiology, Immunology, and Molecular Genetics, University of California, Los Angeles, Los Angeles, United States; Xavier University of Louisiana, New Orleans, United States; Biological Sciences, Florida Gulf Coast University, Fort Myers, United States; Washington State University, Pullman, United States; Biology, Calvin College, Grand Rapids, United States; Biology, Gonzaga University, Spokane, United States; Biology, Gonzaga University, Spokane, United States; Biology, College of St. Scholastica, Duluth, United States; Microbiology and Biotechnology, North Carolina State University, Raleigh, United States; University of Colorado at Boulder, Boulder, United States; Biological Sciences, Lehigh University, Bethlehem, United States; Biology, Illinois Wesleyan University, Bloomington, United States; The Evergreen State College, Olympia, United States; Microbiology and Biotechnology, North Carolina State University, Raleigh, United States; Biological Sciences, Lehigh University, Bethlehem, United States; Biology, University of Louisiana at Monroe, Monroe, United States; Biology, Gettysburg College, Gettysburg, United States; Biology, Culver-Stockton College, Canton, United States; Biology, Smith College, Northampton, United States; Biology, Gonzaga University, Spokane, United States; University of Colorado at Boulder, Boulder, United States; Biology, Gonzaga University, Spokane, United States; Washington State University, Pullman, United States; Xavier University of Louisiana, New Orleans, United States; Xavier University of Louisiana, New Orleans, United States; Biology, University of Puerto Rico - Cayey, Cayey, United States; Microbiology, Miami University, Oxford, United States; Biology, Gonzaga University, Spokane, United States; Biological Sciences, Carnegie Mellon University, Pittsburgh, United States; Virginia Commonwealth University, Richmond, United States; Biology, Washington University in St. Louis, St. Louis, United States; Biology, College of St. Scholastica, Duluth, United States; Microbiology, Miami University, Oxford, United States; Department of Microbiology, Immunology, and Molecular Genetics, University of California, Los Angeles, Los Angeles, United States; Biology, Hope College, Holland, United States; Biology, University of Wisconsin-River Falls, River Falls, United States; Biology, Washington University in St. Louis, St. Louis, United States; Biology, University of Wisconsin-River Falls, River Falls, United States; Biology, University of Puerto Rico - Cayey, Cayey, United States; Ohio State University, Columbus, United States; Molecular and Biomedical Sciences, University of Maine, Honors College, Orono, United States; Montclair State University, Montclair, United States; Biology, College of St. Scholastica, Duluth, United States; Biology, College of Idaho, Caldwell, United States; Biology, University of Alabama Birmingham, Birmingham, United States; Biological Sciences and Geology, Queensboro Community College, Bayside, United States; Biology, Gonzaga University, Spokane, United States; Biology, Montana Tech of the University of Montana, Butte, United States; Morehouse College, Atlanta, United States; The Evergreen State College, Olympia, United States; Ohio State University, Columbus, United States; Microbiology, Miami University, Oxford, United States; University of California Santa Cruz, Santa Cruz, United States; Biological Sciences and Geology, Queensboro Community College, Bayside, United States; Biology, University of Puerto Rico - Cayey, Cayey, United States; Science, Cabrini College, Radnor, United States; Biology, CUNY, Queens College, Queens, United States; Marine Science, Southern Maine Community College, South Portland, United States; Biology, University of Louisiana at Monroe, Monroe, United States; Ohio State University, Columbus, United States; Biology, Culver-Stockton College, Canton, United States; University of Colorado at Boulder, Boulder, United States; Biology, University of Louisiana at Monroe, Monroe, United States; Biology, University of California San Diego, La Jolla, United States; Biology, University of Louisiana at Monroe, Monroe, United States; Biology and Chemistry, Nyack College, Nyack, United States; Microbiology and Molecular Biology, Brigham Young University, Provo, United States; Biology, University of Wisconsin-River Falls, River Falls, United States; Biological Sciences, Carnegie Mellon University, Pittsburgh, United States; Biology, Loyola Marymount University, Los Angeles, United States; Purdue University, West Lafayette, United States; Biological Sciences, University of North Texas, Denton, United States; Biology, Howard College, Washington, DC, United States; University of Colorado at Boulder, Boulder, United States; Ohio State University, Columbus, United States; Biology, University of Louisiana at Monroe, Monroe, United States; Biology, Trinity College, Hartford, United States; Department of Biology, Baylor University, Waco, United States; Biology, University of Louisiana at Monroe, Monroe, United States; Biology, University of Texas at El Paso, El Paso, United States; Biological Sciences, Carnegie Mellon University, Pittsburgh, United States; Biology, University of Puerto Rico - Cayey, Cayey, United States; Biology, Jacksonville State University, Jacksonville, United States; Biology, Saint Joseph's University, Philadelphia, United States; Biology, Gonzaga University, Spokane, United States; Biology, College of William and Mary, Williamsburg, United States; Providence College, Providence, United States; Biology, Illinois Wesleyan University, Bloomington, United States; Ohio State University, Columbus, United States; Microbiology, Miami University, Oxford, United States; Biology, College of Charleston, Charleston, United States; Biology, College of William and Mary, Williamsburg, United States; Biology, Carthage College, Kenosha, United States; Biology, Montana Tech of the University of Montana, Butte, United States; Biology, Illinois Wesleyan University, Bloomington, United States; Biology, Washington University in St. Louis, St. Louis, United States; Biology, University of Alabama Birmingham, Birmingham, United States; Department of Biology, Baylor University, Waco, United States; Biology, University of California San Diego, La Jolla, United States; Biology, University of Alabama Birmingham, Birmingham, United States; Biology, Loyola Marymount University, Los Angeles, United States; The Evergreen State College, Olympia, United States; Department of Microbiology, Immunology, and Molecular Genetics, University of California, Los Angeles, Los Angeles, United States; Biology, University of Texas at El Paso, El Paso, United States; Biology, University of Texas at El Paso, El Paso, United States; Biology, Howard College, Washington, DC, United States; Montclair State University, Montclair, United States; Montclair State University, Montclair, United States; Biology, University of Louisiana at Monroe, Monroe, United States; Biology, Howard College, Washington, DC, United States; Biology, University of California San Diego, La Jolla, United States; Biology, Gonzaga University, Spokane, United States; University of California Santa Cruz, Santa Cruz, United States; Washington State University, Pullman, United States; Biology, University of Wisconsin-River Falls, River Falls, United States; The Evergreen State College, Olympia, United States; Biology, Gonzaga University, Spokane, United States; University of Colorado at Boulder, Boulder, United States; Arts and Sciences Division, University of Maine, Fort Kent, Fort Kent, United States; University of Colorado at Boulder, Boulder, United States; Department of Biology, Baylor University, Waco, United States; Biology, Gonzaga University, Spokane, United States; Biology, Ouachita Baptist University, Arkadelphia, United States; Microbiology, Miami University, Oxford, United States; Department of Biology, Baylor University, Waco, United States; Biology, University of Wisconsin-River Falls, River Falls, United States; Biology, Calvin College, Grand Rapids, United States; Biology, Carthage College, Kenosha, United States; Morehouse College, Atlanta, United States; Department of Microbiology, Immunology, and Molecular Genetics, University of California, Los Angeles, Los Angeles, United States; School of Science and Technology, Georgia Gwinnett College, Lawrenceville, United States; Biology, Howard College, Washington, DC, United States; Biology, University of California San Diego, La Jolla, United States; Biology, University of California San Diego, La Jolla, United States; Department of Microbiology, Immunology, and Molecular Genetics, University of California, Los Angeles, Los Angeles, United States; Biology, University of California San Diego, La Jolla, United States; Biology, University of California San Diego, La Jolla, United States; School of Science and Technology, Georgia Gwinnett College, Lawrenceville, United States; Biology, University of Alabama Birmingham, Birmingham, United States; Biology, University of Louisiana at Monroe, Monroe, United States; University of Colorado at Boulder, Boulder, United States; ISBT, LaSalle University, Philadelphia, United States; University of California Santa Cruz, Santa Cruz, United States; Biology, Saint Joseph's University, Philadelphia, United States; Biology, Gonzaga University, Spokane, United States; School of Science and Technology, Georgia Gwinnett College, Lawrenceville, United States; Biology, Gonzaga University, Spokane, United States; Biology, University of Louisiana at Monroe, Monroe, United States; Purdue University, West Lafayette, United States; Biology, University of Wisconsin-River Falls, River Falls, United States; Biology, University of California San Diego, La Jolla, United States; Ohio State University, Columbus, United States; Biological Sciences, University of Pittsburgh, Pittsburgh, United States; Biological Sciences, Lehigh University, Bethlehem, United States; Biology, Gonzaga University, Spokane, United States; Biology, CUNY, Queens College, Queens, United States; Biological Sciences and Geology, Queensboro Community College, Bayside, United States; Biology, Calvin College, Grand Rapids, United States; Washington State University, Pullman, United States; Biology, University of Alabama Birmingham, Birmingham, United States; Providence College, Providence, United States; Biology, Hope College, Holland, United States; Washington State University, Pullman, United States; Biological Sciences, Carnegie Mellon University, Pittsburgh, United States; Biology, Gonzaga University, Spokane, United States; Biology, Saint Joseph's University, Philadelphia, United States; Montclair State University, Montclair, United States; ISBT, LaSalle University, Philadelphia, United States; Biology, Gonzaga University, Spokane, United States; Biology, University of Puerto Rico - Cayey, Cayey, United States; Biology, University of Texas at El Paso, El Paso, United States; Montclair State University, Montclair, United States; Biology, Howard College, Washington, DC, United States; Morehouse College, Atlanta, United States; Biology, University of Alabama Birmingham, Birmingham, United States; Biology, College of St. Scholastica, Duluth, United States; Biology, University of Louisiana at Monroe, Monroe, United States; Department of Biology, Baylor University, Waco, United States; Biology, Calvin College, Grand Rapids, United States; University of Florida, Gainsville, United States; Biology, Loyola Marymount University, Los Angeles, United States; Biology, University of Puerto Rico - Cayey, Cayey, United States; Biological Sciences, University of North Texas, Denton, United States; Biology, Howard College, Washington, DC, United States; Biological Sciences, University of North Texas, Denton, United States; Biology, Howard College, Washington, DC, United States; Biology, Loyola Marymount University, Los Angeles, United States; Biology, Saint Joseph's University, Philadelphia, United States; Biology, University of Texas at El Paso, El Paso, United States; Biological Sciences, Carnegie Mellon University, Pittsburgh, United States; Biology, Gonzaga University, Spokane, United States; Department of Biology, Baylor University, Waco, United States; Biology, Illinois Wesleyan University, Bloomington, United States; Biology, University of Texas at El Paso, El Paso, United States; Department of Biology, Baylor University, Waco, United States; Biology, University of Puerto Rico - Cayey, Cayey, United States; Biology, University of Puerto Rico - Cayey, Cayey, United States; Biology, University of Puerto Rico - Cayey, Cayey, United States; Ohio State University, Columbus, United States; Biology, Culver-Stockton College, Canton, United States; Biology, Saint Joseph's University, Philadelphia, United States; School of Science and Technology, Georgia Gwinnett College, Lawrenceville, United States; Biology, Calvin College, Grand Rapids, United States; Biology, Gonzaga University, Spokane, United States; Biology, University of Puerto Rico - Cayey, Cayey, United States; Biology, University of Puerto Rico - Cayey, Cayey, United States; Biological Sciences, University of Pittsburgh, Pittsburgh, United States; Western Kentucky University, Bowling Green, United States; Biology, Gonzaga University, Spokane, United States; Biology, Howard College, Washington, DC, United States; Biology Department, Georgia State University, Milledgeville, United States; Biology, University of Puerto Rico - Cayey, Cayey, United States; Virginia Commonwealth University, Richmond, United States; Environmental and Biological Science, University of Maine, Machias, Machias, United States; Biology, University of Texas at El Paso, El Paso, United States; Biology, Howard College, Washington, DC, United States; University of Colorado at Boulder, Boulder, United States; Biology, Saint Joseph's University, Philadelphia, United States; University of Colorado at Boulder, Boulder, United States; Biological Sciences, University of Pittsburgh, Pittsburgh, United States; Biological Sciences, Carnegie Mellon University, Pittsburgh, United States; Biology, Saint Joseph's University, Philadelphia, United States; Biology, University of California San Diego, La Jolla, United States; Biological Sciences and Geology, Queensboro Community College, Bayside, United States; Biological Sciences, University of Pittsburgh, Pittsburgh, United States; Biology and Chemistry, Nyack College, Nyack, United States; Biology, Gonzaga University, Spokane, United States; Biology, Spelman College, Atlanta, United States; Biological Sciences, Carnegie Mellon University, Pittsburgh, United States; Western Kentucky University, Bowling Green, United States; Microbiology, Miami University, Oxford, United States; Montclair State University, Montclair, United States; Biology, Loyola Marymount University, Los Angeles, United States; Department of Biology, Baylor University, Waco, United States; Biology, University of Alabama Birmingham, Birmingham, United States; Biology, Gettysburg College, Gettysburg, United States; Montclair State University, Montclair, United States; Montclair State University, Montclair, United States; Biology, College of Charleston, Charleston, United States; Biology, Saint Joseph's University, Philadelphia, United States; Biology, Loyola Marymount University, Los Angeles, United States; Biology, University of Alabama Birmingham, Birmingham, United States; Biology, University of Alabama Birmingham, Birmingham, United States; Biology, Washington University in St. Louis, St. Louis, United States; Biology, Illinois Wesleyan University, Bloomington, United States; Biology, Loyola Marymount University, Los Angeles, United States; Biology, North Carolina Central University, Durham, United States; Biology, Carthage College, Kenosha, United States; Biology, University of Alabama Birmingham, Birmingham, United States; Microbiology and Molecular Biology, Brigham Young University, Provo, United States; University of Colorado at Boulder, Boulder, United States; Biology, Carthage College, Kenosha, United States; Biology, Howard College, Washington, DC, United States; Department of Biology, Baylor University, Waco, United States; Biological Sciences, Carnegie Mellon University, Pittsburgh, United States; Biology, Montana Tech of the University of Montana, Butte, United States; Biology, Montana Tech of the University of Montana, Butte, United States; Biology, Jacksonville State University, Jacksonville, United States; Morehouse College, Atlanta, United States; Biology, Smith College, Northampton, United States; Biology, Gonzaga University, Spokane, United States; Biological Sciences, University of North Texas, Denton, United States; University of Colorado at Boulder, Boulder, United States; Xavier University of Louisiana, New Orleans, United States; University of California Santa Cruz, Santa Cruz, United States; Biology and Medicine, Brown University, Providence, United States; Biology, College of Charleston, Charleston, United States; Biology, University of Puerto Rico - Cayey, Cayey, United States; Biology, University of California San Diego, La Jolla, United States; School of Science and Technology, Georgia Gwinnett College, Lawrenceville, United States; University of Florida, Gainsville, United States; Biology, University of Puerto Rico - Cayey, Cayey, United States; Biology, University of Puerto Rico - Cayey, Cayey, United States; Biology, University of Puerto Rico - Cayey, Cayey, United States; Biological Sciences, Lehigh University, Bethlehem, United States; Providence College, Providence, United States; Biology, College of Charleston, Charleston, United States; Biology, University of California San Diego, La Jolla, United States; Biological Sciences, Lehigh University, Bethlehem, United States; Biology, Gonzaga University, Spokane, United States; Biology, College of St. Scholastica, Duluth, United States; The Evergreen State College, Olympia, United States; Biology, Loyola Marymount University, Los Angeles, United States; Western Kentucky University, Bowling Green, United States; Biology, Culver-Stockton College, Canton, United States; Biology, Carthage College, Kenosha, United States; Biology, Gonzaga University, Spokane, United States; Biological Sciences, Carnegie Mellon University, Pittsburgh, United States; University of Maine, Honors College, Orono, United States; Xavier University of Louisiana, New Orleans, United States; University of Maine, Honors College, Orono, United States; Biology, University of California San Diego, La Jolla, United States; Biology, College of Charleston, Charleston, United States; University of California Santa Cruz, Santa Cruz, United States; Western Kentucky University, Bowling Green, United States; Science, Cabrini College, Radnor, United States; Microbiology and Biotechnology, North Carolina State University, Raleigh, United States; Purdue University, West Lafayette, United States; Biology, Nebraska Wesleyan University, Lincoln, Nebraska, United States; Biology, Gettysburg College, Gettysburg, United States; Biology, Gonzaga University, Spokane, United States; Biology, Loyola Marymount University, Los Angeles, United States; Purdue University, West Lafayette, United States; Biology Department, Georgia State University, Milledgeville, United States; Providence College, Providence, United States; Biological Sciences, University of North Texas, Denton, United States; Biology, University of Texas at El Paso, El Paso, United States; Biology, Illinois Wesleyan University, Bloomington, United States; Biology, University of California San Diego, La Jolla, United States; Biology, Illinois Wesleyan University, Bloomington, United States; The Evergreen State College, Olympia, United States; Biology, Loyola Marymount University, Los Angeles, United States; Microbiology, Miami University, Oxford, United States; Biology, College of Charleston, Charleston, United States; Biology, Jacksonville State University, Jacksonville, United States; Biological Sciences, University of North Texas, Denton, United States; Department of Biology, Bucknell University, Lewisburg, United States; Biology, Calvin College, Grand Rapids, United States; Biology, University of California San Diego, La Jolla, United States; University of California Santa Cruz, Santa Cruz, United States; Biology, Ouachita Baptist University, Arkadelphia, United States; Biology, Gettysburg College, Gettysburg, United States; Biology, University of California San Diego, La Jolla, United States; Biology, University of California San Diego, La Jolla, United States; Biology, North Carolina Central University, Durham, United States; Biology, Calvin College, Grand Rapids, United States; Biology, Trinity College, Hartford, United States; Biology, Trinity College, Hartford, United States; Biology, College of St. Scholastica, Duluth, United States; Biology, Howard College, Washington, DC, United States; University of California Santa Cruz, Santa Cruz, United States; Biology, University of Puerto Rico - Cayey, Cayey, United States; University of Colorado at Boulder, Boulder, United States; Biology, Hope College, Holland, United States; Biology, College of Charleston, Charleston, United States; Western Kentucky University, Bowling Green, United States; Biology, University of Texas at El Paso, El Paso, United States; Biology, Saint Joseph's University, Philadelphia, United States; Biological Sciences, Carnegie Mellon University, Pittsburgh, United States; Ohio State University, Columbus, United States; Science, Cabrini College, Radnor, United States; The Evergreen State College, Olympia, United States; Biology, North Carolina Central University, Durham, United States; Morehouse College, Atlanta, United States; Biology, Jacksonville State University, Jacksonville, United States; Xavier University of Louisiana, New Orleans, United States; Honors Program, Florida Gulf Coast University, Fort Myers, United States; Biology, Gonzaga University, Spokane, United States; Biology, Calvin College, Grand Rapids, United States; Biology, CUNY, Queens College, Queens, United States; Biological Sciences, Lehigh University, Bethlehem, United States; Biology, Merrimack College, North Andover, United States; Morehouse College, Atlanta, United States; Biology, Gonzaga University, Spokane, United States; Biological Sciences, University of North Texas, Denton, United States; Biology, University of Puerto Rico - Cayey, Cayey, United States; Biology, Hope College, Holland, United States; Biology, College of St. Scholastica, Duluth, United States; Washington State University, Pullman, United States; Biological Sciences and Geology, Queensboro Community College, Bayside, United States; Biology, Montana Tech of the University of Montana, Butte, United States; Washington State University, Pullman, United States; Biological Sciences, University of Pittsburgh, Pittsburgh, United States; Biology, University of Alabama Birmingham, Birmingham, United States; Montclair State University, Montclair, United States; Microbiology, Miami University, Oxford, United States; Biology, University of Wisconsin-River Falls, River Falls, United States; Biology, Howard College, Washington, DC, United States; Biology, Saint Joseph's University, Philadelphia, United States; Biology, Washington University in St. Louis, St. Louis, United States; Biology, Spelman College, Atlanta, United States; Biology, University of California San Diego, La Jolla, United States; University of Colorado at Boulder, Boulder, United States; Biology, College of Charleston, Charleston, United States; Microbiology, Howard College, Washington, DC, United States; Biology, North Carolina Central University, Durham, United States; Biology, College of St. Scholastica, Duluth, United States; Biology, University of Puerto Rico - Cayey, Cayey, United States; Biology, University of Puerto Rico - Cayey, Cayey, United States; Biology, Washington University in St. Louis, St. Louis, United States; Biology, University of Puerto Rico - Cayey, Cayey, United States; Biology, College of St. Scholastica, Duluth, United States; Microbiology, Miami University, Oxford, United States; Biological Sciences, University of North Texas, Denton, United States; Biology, Saint Joseph's University, Philadelphia, United States; Biology, College of Charleston, Charleston, United States; University of Colorado at Boulder, Boulder, United States; Biology, Culver-Stockton College, Canton, United States; Biology, Trinity College, Hartford, United States; Biology, Illinois Wesleyan University, Bloomington, United States; Biology, University of Texas at El Paso, El Paso, United States; Washington State University, Pullman, United States; University of Florida, Gainsville, United States; Biology, University of Puerto Rico - Cayey, Cayey, United States; Biological Sciences and Geology, Queensboro Community College, Bayside, United States; Biology, University of Puerto Rico - Cayey, Cayey, United States; Biology, University of Puerto Rico - Cayey, Cayey, United States; Biology, Gettysburg College, Gettysburg, United States; The Evergreen State College, Olympia, United States; Natural Sciences, Del Mar College, Corpus Christi, United States; Biology, University of Alabama Birmingham, Birmingham, United States; Biology, North Carolina Central University, Durham, United States; Montclair State University, Montclair, United States; Biology, University of Texas at El Paso, El Paso, United States; Biology, Washington University in St. Louis, St. Louis, United States; Biology, Smith College, Northampton, United States; University of California Santa Cruz, Santa Cruz, United States; Biology, Gettysburg College, Gettysburg, United States; Biological Sciences and Geology, Queensboro Community College, Bayside, United States; Biology, College of Charleston, Charleston, United States; Biological Sciences and Geology, Queensboro Community College, Bayside, United States; Microbiology, Miami University, Oxford, United States; Department of Microbiology, Immunology, and Molecular Genetics, University of California, Los Angeles, Los Angeles, United States; Xavier University of Louisiana, New Orleans, United States; Biology, University of Wisconsin-River Falls, River Falls, United States; Biology, University of Louisiana at Monroe, Monroe, United States; Biology, Calvin College, Grand Rapids, United States; Biology, Washington University in St. Louis, St. Louis, United States; Biology, Gonzaga University, Spokane, United States; Providence College, Providence, United States; Biological Sciences, Lehigh University, Bethlehem, United States; Ohio State University, Columbus, United States; Biology, Carthage College, Kenosha, United States; Ohio State University, Columbus, United States; Biological Sciences, Carnegie Mellon University, Pittsburgh, United States; Microbiology, Miami University, Oxford, United States; Virginia Commonwealth University, Richmond, United States; University of Colorado at Boulder, Boulder, United States; Biology, Chadron State College, Chadron, United States; Biology, University of Texas at El Paso, El Paso, United States; Biology, University of Puerto Rico - Cayey, Cayey, United States; Biology, University of Puerto Rico - Cayey, Cayey, United States; Biology, Ouachita Baptist University, Arkadelphia, United States; Microbiology, Miami University, Oxford, United States; The Evergreen State College, Olympia, United States; Biology, Loyola Marymount University, Los Angeles, United States; Biology, Loyola Marymount University, Los Angeles, United States; Biology, Calvin College, Grand Rapids, United States; Biology, Washington University in St. Louis, St. Louis, United States; Biology, University of California San Diego, La Jolla, United States; Washington State University, Pullman, United States; Biology, University of California San Diego, La Jolla, United States; Biology, College of Charleston, Charleston, United States; Biology, Ouachita Baptist University, Arkadelphia, United States; Biology, University of Louisiana at Monroe, Monroe, United States; Purdue University, West Lafayette, United States; Biology, Gonzaga University, Spokane, United States; ISBT, LaSalle University, Philadelphia, United States; Department of Biology, Baylor University, Waco, United States; Western Kentucky University, Bowling Green, United States; University of Florida, Gainsville, United States; Biology, University of Louisiana at Monroe, Monroe, United States; Biology, University of Puerto Rico - Cayey, Cayey, United States; Biology, University of Puerto Rico - Cayey, Cayey, United States; Biology, University of Puerto Rico - Cayey, Cayey, United States; Biology, University of Puerto Rico - Cayey, Cayey, United States; Biology, University of Puerto Rico - Cayey, Cayey, United States; Biology, University of Puerto Rico - Cayey, Cayey, United States; Biology, University of Puerto Rico - Cayey, Cayey, United States; Biology, University of Puerto Rico - Cayey, Cayey, United States; Biology, University of Puerto Rico - Cayey, Cayey, United States; Biology, University of Puerto Rico - Cayey, Cayey, United States; Biology, University of Puerto Rico - Cayey, Cayey, United States; Biology, University of Puerto Rico - Cayey, Cayey, United States; Biology, University of Puerto Rico - Cayey, Cayey, United States; Biology, University of Puerto Rico - Cayey, Cayey, United States; Montclair State University, Montclair, United States; Biology, University of Louisiana at Monroe, Monroe, United States; University of Maine, Honors College, Orono, United States; Biological Sciences, University of North Texas, Denton, United States; Biological Sciences, Lehigh University, Bethlehem, United States; Natural Sciences, Del Mar College, Corpus Christi, United States; Biology, University of Wisconsin-River Falls, River Falls, United States; Biology, Howard College, Washington, DC, United States; Biology, University of Alabama Birmingham, Birmingham, United States; Biology, University of Louisiana at Monroe, Monroe, United States; Biological Sciences, University of North Texas, Denton, United States; Division of Natural and Health Sciences, Seton Hill University, Greensburg, United States; Biology, Gettysburg College, Gettysburg, United States; Biology, Loyola Marymount University, Los Angeles, United States; Biology, Trinity College, Hartford, United States; Biology, University of Texas at El Paso, El Paso, United States; Biological Sciences, University of North Texas, Denton, United States; Department of Biology, Baylor University, Waco, United States; University of California Santa Cruz, Santa Cruz, United States; Biology, University of Puerto Rico - Cayey, Cayey, United States; Biology, University of Puerto Rico - Cayey, Cayey, United States; Biology, University of Puerto Rico - Cayey, Cayey, United States; Biology, University of Puerto Rico - Cayey, Cayey, United States; Providence College, Providence, United States; Biology, College of St. Scholastica, Duluth, United States; Biological Sciences, Carnegie Mellon University, Pittsburgh, United States; Biology, University of Texas at El Paso, El Paso, United States; Purdue University, West Lafayette, United States; Biology, Saint Joseph's University, Philadelphia, United States; Biology, University of Texas at El Paso, El Paso, United States; Biology, University of Louisiana at Monroe, Monroe, United States; Honors Program, Florida Gulf Coast University, Fort Myers, United States; Biology, University of Texas at El Paso, El Paso, United States; Department of Biology, Baylor University, Waco, United States; Department of Biology, Baylor University, Waco, United States; Xavier University of Louisiana, New Orleans, United States; University of Florida, Gainsville, United States; Biology, Smith College, Northampton, United States; Ohio State University, Columbus, United States; Biological Sciences, Lehigh University, Bethlehem, United States; School of Science and Technology, Georgia Gwinnett College, Lawrenceville, United States; Biology, University of Puerto Rico - Cayey, Cayey, United States; University of California Santa Cruz, Santa Cruz, United States; University of California Santa Cruz, Santa Cruz, United States; Xavier University of Louisiana, New Orleans, United States; Biology, University of California San Diego, La Jolla, United States; Ohio State University, Columbus, United States; Montclair State University, Montclair, United States; Biology, Loyola Marymount University, Los Angeles, United States; Washington State University, Pullman, United States; University of California Santa Cruz, Santa Cruz, United States; Biology, Gonzaga University, Spokane, United States; Biology, Gonzaga University, Spokane, United States; ISBT, LaSalle University, Philadelphia, United States; University of Florida, Gainsville, United States; Biological Sciences, University of North Texas, Denton, United States; Biology, Gonzaga University, Spokane, United States; Biological Sciences, Carnegie Mellon University, Pittsburgh, United States; Natural Sciences, University of Houston-Downtown, Houston, United States; Biology, College of William and Mary, Williamsburg, United States; Biology, University of Alabama Birmingham, Birmingham, United States; Montclair State University, Montclair, United States; ISBT, LaSalle University, Philadelphia, United States; Montclair State University, Montclair, United States; Biology, University of Louisiana at Monroe, Monroe, United States; Biology, Jacksonville State University, Jacksonville, United States; Biology, College of St. Scholastica, Duluth, United States; Biology, Loyola Marymount University, Los Angeles, United States; Biology, University of Louisiana at Monroe, Monroe, United States; Biology, University of Texas at El Paso, El Paso, United States; Biology, University of Puerto Rico - Cayey, Cayey, United States; Department of Microbiology, Immunology, and Molecular Genetics, University of California, Los Angeles, Los Angeles, United States; Department of Biological Sciences, University of Maryland, Baltimore County, Baltimore, United States; The Evergreen State College, Olympia, United States; Biological Sciences, Lehigh University, Bethlehem, United States; Biology, Loyola Marymount University, Los Angeles, United States; Biology, Loyola Marymount University, Los Angeles, United States; Biology, University of Puerto Rico - Cayey, Cayey, United States; Biology, University of Puerto Rico - Cayey, Cayey, United States; Biology, University of Texas at El Paso, El Paso, United States; Biology and Chemistry, Nyack College, Nyack, United States; Biological Sciences, University of North Texas, Denton, United States; Xavier University of Louisiana, New Orleans, United States; Microbiology and Biotechnology, North Carolina State University, Raleigh, United States; Biology, Smith College, Northampton, United States; Biology, Loyola Marymount University, Los Angeles, United States; Biological Sciences, Carnegie Mellon University, Pittsburgh, United States; Department of Biology, Baylor University, Waco, United States; Biology, Washington University in St. Louis, St. Louis, United States; Western Kentucky University, Bowling Green, United States; Western Kentucky University, Bowling Green, United States; Biology, College of Charleston, Charleston, United States; Biology, Hope College, Holland, United States; Biological Sciences, University of North Texas, Denton, United States; Biological Sciences, University of Pittsburgh, Pittsburgh, United States; Biology, Culver-Stockton College, Canton, United States; Biology, College of St. Scholastica, Duluth, United States; Biology, Calvin College, Grand Rapids, United States; Biology, Saint Joseph's University, Philadelphia, United States; Biology, University of Louisiana at Monroe, Monroe, United States; University of Colorado at Boulder, Boulder, United States; Biological Sciences, Lehigh University, Bethlehem, United States; Biology, Calvin College, Grand Rapids, United States; Biology, College of Charleston, Charleston, United States; Ohio State University, Columbus, United States; Biology, Gonzaga University, Spokane, United States; Washington State University, Pullman, United States; Washington State University, Pullman, United States; Integrative Biology, Oregon State University, Corvallis, United States; Biology, Washington University in St. Louis, St. Louis, United States; Biological Sciences, Carnegie Mellon University, Pittsburgh, United States; Biology, University of Louisiana at Monroe, Monroe, United States; Western Kentucky University, Bowling Green, United States; Purdue University, West Lafayette, United States; Division of Natural and Health Sciences, Seton Hill University, Greensburg, United States; Ohio State University, Columbus, United States; Ohio State University, Columbus, United States; Biology, Gonzaga University, Spokane, United States; Biology, Calvin College, Grand Rapids, United States; University of Florida, Gainsville, United States; Washington State University, Pullman, United States; Biology, Gonzaga University, Spokane, United States; Ohio State University, Columbus, United States; Biology, University of Wisconsin-River Falls, River Falls, United States; Biology, Calvin College, Grand Rapids, United States; Biology, Washington University in St. Louis, St. Louis, United States; Biology, Nebraska Wesleyan University, Lincoln, Nebraska, United States; Biology, Gonzaga University, Spokane, United States; Washington State University, Pullman, United States; Providence College, Providence, United States; Biological Sciences, University of Pittsburgh, Pittsburgh, United States; Providence College, Providence, United States; Biology, University of Louisiana at Monroe, Monroe, United States; Department of Biology, Baylor University, Waco, United States; Biology and Chemistry, Nyack College, Nyack, United States; Biology, College of Charleston, Charleston, United States; Biology, Culver-Stockton College, Canton, United States; Western Kentucky University, Bowling Green, United States; Microbiology and Molecular Biology, Brigham Young University, Provo, United States; Montclair State University, Montclair, United States; Biology, Culver-Stockton College, Canton, United States; Biology, University of California San Diego, La Jolla, United States; Biology, College of Charleston, Charleston, United States; Biology, University of Texas at El Paso, El Paso, United States; Biology, Gonzaga University, Spokane, United States; Science, Cabrini College, Radnor, United States; University of California Santa Cruz, Santa Cruz, United States; Biological Sciences and Geology, Queensboro Community College, Bayside, United States; Biological Sciences, Carnegie Mellon University, Pittsburgh, United States; University of Maine, Honors College, Orono, United States; Biology, Gonzaga University, Spokane, United States; Biological Sciences, Carnegie Mellon University, Pittsburgh, United States; Biology, Washington University in St. Louis, St. Louis, United States; Ohio State University, Columbus, United States; Biology, University of Alabama Birmingham, Birmingham, United States; Virginia Commonwealth University, Richmond, United States; Department of Biology, Baylor University, Waco, United States; Western Kentucky University, Bowling Green, United States; Biology and Medicine, Brown University, Providence, United States; University of California Santa Cruz, Santa Cruz, United States; Western Kentucky University, Bowling Green, United States; University of Florida, Gainsville, United States; Washington State University, Pullman, United States; Biology, Nebraska Wesleyan University, Lincoln, Nebraska, United States; Biological Sciences, Carnegie Mellon University, Pittsburgh, United States; Microbiology and Molecular Biology, Brigham Young University, Provo, United States; Biological Sciences, Carnegie Mellon University, Pittsburgh, United States; Microbiology and Molecular Biology, Brigham Young University, Provo, United States; Biology, Gonzaga University, Spokane, United States; University of Maine, Honors College, Orono, United States; Biological Sciences, University of North Texas, Denton, United States; Department of Microbiology, Immunology, and Molecular Genetics, University of California, Los Angeles, Los Angeles, United States; Ohio State University, Columbus, United States; Biology, Culver-Stockton College, Canton, United States; Biology, Spelman College, Atlanta, United States; Biology, University of California San Diego, La Jolla, United States; Biology, University of California San Diego, La Jolla, United States; Biological Sciences, Carnegie Mellon University, Pittsburgh, United States; Biological Sciences and Geology, Queensboro Community College, Bayside, United States; Ohio State University, Columbus, United States; Ohio State University, Columbus, United States; Washington State University, Pullman, United States; Biology, Illinois Wesleyan University, Bloomington, United States; The Evergreen State College, Olympia, United States; Biology, Howard College, Washington, DC, United States; Biological Sciences, University of North Texas, Denton, United States; University of Florida, Gainsville, United States; Natural Sciences, University of Houston-Downtown, Houston, United States; University of Florida, Gainsville, United States; Biological Sciences, Lehigh University, Bethlehem, United States; Biology, Washington University in St. Louis, St. Louis, United States; Biological Sciences, University of Pittsburgh, Pittsburgh, United States; Biology, University of Texas at El Paso, El Paso, United States; Montclair State University, Montclair, United States; Department of Microbiology, Immunology, and Molecular Genetics, University of California, Los Angeles, Los Angeles, United States; Biology and Chemistry, Nyack College, Nyack, United States; Department of Biology, Baylor University, Waco, United States; Biological Sciences, Carnegie Mellon University, Pittsburgh, United States; Biology, University of Louisiana at Monroe, Monroe, United States; Biological Sciences, University of North Texas, Denton, United States; University of California Santa Cruz, Santa Cruz, United States; Biology, Loyola Marymount University, Los Angeles, United States; Biology, University of Louisiana at Monroe, Monroe, United States; Biology, Washington University in St. Louis, St. Louis, United States; Biological Sciences, Lehigh University, Bethlehem, United States; Biology, University of Alabama Birmingham, Birmingham, United States; Biology, Gettysburg College, Gettysburg, United States; Biology and Medicine, Brown University, Providence, United States; Biology, University of Wisconsin-River Falls, River Falls, United States; Ohio State University, Columbus, United States; School of Science and Technology, Georgia Gwinnett College, Lawrenceville, United States; biology, North Carolina Central University, Durham, United States; Biology, University of Wisconsin-River Falls, River Falls, United States; Biological Sciences, University of North Texas, Denton, United States; Biology, Illinois Wesleyan University, Bloomington, United States; Biology, Saint Joseph's University, Philadelphia, United States; Biology, College of Charleston, Charleston, United States; Environmental and Biological Science, University of Maine, Machias, Machias, United States; Biology, College of William and Mary, Williamsburg, United States; Biology, College of Charleston, Charleston, United States; University of California Santa Cruz, Santa Cruz, United States; Biology, College of Charleston, Charleston, United States; Biology, Saint Joseph's University, Philadelphia, United States; Biology, Saint Joseph's University, Philadelphia, United States; Biology, Jacksonville State University, Jacksonville, United States; Department of Biology, Baylor University, Waco, United States; ISBT, LaSalle University, Philadelphia, United States; Biology, Ouachita Baptist University, Arkadelphia, United States; The Evergreen State College, Olympia, United States; Morehouse College, Atlanta, United States; Microbiology and Molecular Biology, Brigham Young University, Provo, United States; Science, Cabrini College, Radnor, United States; Biology, College of Charleston, Charleston, United States; Division of Natural and Health Sciences, Seton Hill University, Greensburg, United States; University of Florida, Gainsville, United States; Biology, Gonzaga University, Spokane, United States; University of Maine, Honors College, Orono, United States; University of Maine, Honors College, Orono, United States; Biological Sciences, Carnegie Mellon University, Pittsburgh, United States; Biology, Illinois Wesleyan University, Bloomington, United States; Biology, University of Wisconsin-River Falls, River Falls, United States; Department of Biology, Baylor University, Waco, United States; University of Colorado at Boulder, Boulder, United States; Biology, University of Texas at El Paso, El Paso, United States; Biology, Gonzaga University, Spokane, United States; Biology, Gonzaga University, Spokane, United States; Biology, Saint Joseph's University, Philadelphia, United States; Biology, University of Wisconsin-River Falls, River Falls, United States; Biology, Saint Joseph's University, Philadelphia, United States; Biology, Spelman College, Atlanta, United States; Biology, Carthage College, Kenosha, United States; Natural Sciences, Del Mar College, Corpus Christi, United States; Biology, Saint Joseph's University, Philadelphia, United States; Ohio State University, Columbus, United States; Biological Sciences, Carnegie Mellon University, Pittsburgh, United States; Department of Biology, Baylor University, Waco, United States; Western Kentucky University, Bowling Green, United States; Division of Natural and Health Sciences, Seton Hill University, Greensburg, United States; Washington State University, Pullman, United States; Montclair State University, Montclair, United States; Western Kentucky University, Bowling Green, United States; Ohio State University, Columbus, United States; Biology, Carthage College, Kenosha, United States; Pedagogy, University of Puerto Rico - Cayey, Cayey, United States; Department of Microbiology, Immunology, and Molecular Genetics, University of California, Los Angeles, Los Angeles, United States; Department of Biology, Baylor University, Waco, United States; Biology, University of California San Diego, La Jolla, United States; Biological Sciences, Lehigh University, Bethlehem, United States; Microbiology, Miami University, Oxford, United States; Biology, College of Idaho, Caldwell, United States; Biology, University of California San Diego, La Jolla, United States; School of Science and Technology, Georgia Gwinnett College, Lawrenceville, United States; Xavier University of Louisiana, New Orleans, United States; Biology, Calvin College, Grand Rapids, United States; Division of Natural and Health Sciences, Seton Hill University, Greensburg, United States; Microbiology and Biotechnology, North Carolina State University, Raleigh, United States; Ohio State University, Columbus, United States; University of California Santa Cruz, Santa Cruz, United States; Biology, Gonzaga University, Spokane, United States; Biological Sciences, University of North Texas, Denton, United States; Purdue University, West Lafayette, United States; Biology, Saint Joseph's University, Philadelphia, United States; Department of Biology, Bucknell University, Lewisburg, United States; Biology, Carthage College, Kenosha, United States; University of California Santa Cruz, Santa Cruz, United States; Biology, University of Louisiana at Monroe, Monroe, United States; Biology, Washington University in St. Louis, St. Louis, United States; Biology, Ouachita Baptist University, Arkadelphia, United States; Biology, Hope College, Holland, United States; Department of Biological Sciences, University of Maryland, Baltimore County, Baltimore, United States; University of California Santa Cruz, Santa Cruz, United States; University of Colorado at Boulder, Boulder, United States; University of Maine, Honors College, Orono, United States; Biological Sciences, University of North Texas, Denton, United States; Microbiology, Miami University, Oxford, United States; Biology, Nebraska Wesleyan University, Lincoln, Nebraska, United States; Biology, College of St. Scholastica, Duluth, United States; Montclair State University, Montclair, United States; Division of Natural and Health Sciences, Seton Hill University, Greensburg, United States; Department of Biology, Baylor University, Waco, United States; Xavier University of Louisiana, New Orleans, United States; Biology, University of Alabama Birmingham, Birmingham, United States; University of Florida, Gainsville, United States; Biological Sciences, Lehigh University, Bethlehem, United States; Montclair State University, Montclair, United States; Biological Sciences, University of North Texas, Denton, United States; Biology, Trinity College, Hartford, United States; Xavier University of Louisiana, New Orleans, United States; Biological Sciences, Lehigh University, Bethlehem, United States; Biology, Saint Joseph's University, Philadelphia, United States; Biological Sciences and Geology, Queensboro Community College, Bayside, United States; Biology, College of Idaho, Caldwell, United States; Biology, Gonzaga University, Spokane, United States; Department of Microbiology, Immunology, and Molecular Genetics, University of California, Los Angeles, Los Angeles, United States; Biology, College of Charleston, Charleston, United States; University of Colorado at Boulder, Boulder, United States; Department of Microbiology, Immunology, and Molecular Genetics, University of California, Los Angeles, Los Angeles, United States; Montclair State University, Montclair, United States; Biology, University of Texas at El Paso, El Paso, United States; Biology, Gonzaga University, Spokane, United States; Biology, Loyola Marymount University, Los Angeles, United States; Department of Microbiology, Immunology, and Molecular Genetics, University of California, Los Angeles, Los Angeles, United States; Biology, University of California San Diego, La Jolla, United States; Biology, Gettysburg College, Gettysburg, United States; Biology, Hope College, Holland, United States; Marine Science, Southern Maine Community College, South Portland, United States; Biological Sciences, University of North Texas, Denton, United States; Biological Sciences, Lehigh University, Bethlehem, United States; Biology, Smith College, Northampton, United States; Biology and Medicine, Brown University, Providence, United States; Integrative Biology, Oregon State University, Corvallis, United States; Biology, University of Wisconsin-River Falls, River Falls, United States; Microbiology, Miami University, Oxford, United States; Biology, Hope College, Holland, United States; ISBT, LaSalle University, Philadelphia, United States; University of Colorado at Boulder, Boulder, United States; Biology, Saint Joseph's University, Philadelphia, United States; Integrated Science & Technology, James Madison University, Harrisonburg, United States; Washington State University, Pullman, United States; Biology, University of Texas at El Paso, El Paso, United States; Xavier University of Louisiana, New Orleans, United States; Biology and Chemistry, Nyack College, Nyack, United States; Biological Sciences, Carnegie Mellon University, Pittsburgh, United States; Biology, Saint Joseph's University, Philadelphia, United States; Biology, University of Texas at El Paso, El Paso, United States; University of Maine, Honors College, Orono, United States; Department of Biology, Bucknell University, Lewisburg, United States; Biological Sciences, Carnegie Mellon University, Pittsburgh, United States; Biology, College of Charleston, Charleston, United States; Biology, University of Alabama Birmingham, Birmingham, United States; Montclair State University, Montclair, United States; Montclair State University, Montclair, United States; Biology, University of Louisiana at Monroe, Monroe, United States; Biological Sciences, Carnegie Mellon University, Pittsburgh, United States; Biology, University of Louisiana at Monroe, Monroe, United States; University of Colorado at Boulder, Boulder, United States; Biology, University of Alabama Birmingham, Birmingham, United States; Biology, Illinois Wesleyan University, Bloomington, United States; Purdue University, West Lafayette, United States; The Evergreen State College, Olympia, United States; Biology, University of California San Diego, La Jolla, United States; Department of Microbiology, Immunology, and Molecular Genetics, University of California, Los Angeles, Los Angeles, United States; Biology, Carthage College, Kenosha, United States; Biology, Gettysburg College, Gettysburg, United States; Biological Sciences, Lehigh University, Bethlehem, United States; ISBT, LaSalle University, Philadelphia, United States; Biology, University of California San Diego, La Jolla, United States; Western Kentucky University, Bowling Green, United States; Biological Sciences, University of Pittsburgh, Pittsburgh, United States; Biological Sciences, University of Pittsburgh, Pittsburgh, United States; Montclair State University, Montclair, United States; Biology, Carthage College, Kenosha, United States; Biology, Washington University in St. Louis, St. Louis, United States; Department of Biology, Bucknell University, Lewisburg, United States; Biology, CUNY, Queens College, Queens, United States; Biology, Gonzaga University, Spokane, United States; Biology, Gettysburg College, Gettysburg, United States; Department of Microbiology, Immunology, and Molecular Genetics, University of California, Los Angeles, Los Angeles, United States; Biology, University of Alabama Birmingham, Birmingham, United States; Biology, Smith College, Northampton, United States; Biological Sciences, University of North Texas, Denton, United States; Biology, University of Texas at El Paso, El Paso, United States; Biology, Gonzaga University, Spokane, United States; Biology, Loyola Marymount University, Los Angeles, United States; Ohio State University, Columbus, United States; School of Science and Technology, Georgia Gwinnett College, Lawrenceville, United States; Biological Sciences, University of North Texas, Denton, United States; Biology, University of Louisiana at Monroe, Monroe, United States; Biology, University of California San Diego, La Jolla, United States; Biology, College of Charleston, Charleston, United States; Biology, Washington University in St. Louis, St. Louis, United States; Department of Microbiology, Immunology, and Molecular Genetics, University of California, Los Angeles, Los Angeles, United States; Biology, University of Louisiana at Monroe, Monroe, United States; Biology, Saint Joseph's University, Philadelphia, United States; Biology, Hope College, Holland, United States; University of Colorado at Boulder, Boulder, United States; Biology, University of Louisiana at Monroe, Monroe, United States; Washington State University, Pullman, United States; Biological Sciences, University of North Texas, Denton, United States; Purdue University, West Lafayette, United States; Biology, University of Alabama Birmingham, Birmingham, United States; Biology, University of California San Diego, La Jolla, United States; Biological Sciences and Geology, Queensboro Community College, Bayside, United States; Biological Sciences, Carnegie Mellon University, Pittsburgh, United States; Biological Sciences and Geology, Queensboro Community College, Bayside, United States; The Evergreen State College, Olympia, United States; Montclair State University, Montclair, United States; Biology, College of Charleston, Charleston, United States; University of Florida, Gainsville, United States; Biology, University of Wisconsin-River Falls, River Falls, United States; University of Florida, Gainsville, United States; Biology, Culver-Stockton College, Canton, United States; Xavier University of Louisiana, New Orleans, United States; Biology, University of Alabama Birmingham, Birmingham, United States; Biological Sciences, Lehigh University, Bethlehem, United States; Biology, Washington University in St. Louis, St. Louis, United States; Montclair State University, Montclair, United States; Biology, University of California San Diego, La Jolla, United States; Biology, University of Alabama Birmingham, Birmingham, United States; Biology, Howard College, Washington, DC, United States; Biology, Gettysburg College, Gettysburg, United States; Biology, Gonzaga University, Spokane, United States; Biology, Illinois Wesleyan University, Bloomington, United States; Washington State University, Pullman, United States; Biology, Hope College, Holland, United States; Biology, University of Louisiana at Monroe, Monroe, United States; Biology, Washington University in St. Louis, St. Louis, United States; Biology, Loyola Marymount and University of Detroit, Los Angeles, United States; Biology, University of Texas at El Paso, El Paso, United States; Natural Sciences, University of Houston-Downtown, Houston, United States; University of Maine, Honors College, Orono, United States; University of California Santa Cruz, Santa Cruz, United States; Biology, Hope College, Holland, United States; Biology, Gonzaga University, Spokane, United States; Biology, Gettysburg College, Gettysburg, United States; University of California Santa Cruz, Santa Cruz, United States; Biology and Chemistry, Nyack College, Nyack, United States; Biology, Calvin College, Grand Rapids, United States; Xavier University of Louisiana, New Orleans, United States; Biology, Calvin College, Grand Rapids, United States; Biology, Hope College, Holland, United States; University of Colorado at Boulder, Boulder, United States; Biology, Hope College, Holland, United States; Biology, Gonzaga University, Spokane, United States; Biology, Calvin College, Grand Rapids, United States; Biology, Merrimack College, North Andover, United States; biology, North Carolina Central University, Durham, United States; Biology, University of California San Diego, La Jolla, United States; Biology, University of Puerto Rico - Cayey, Cayey, United States; Biological Sciences, Lehigh University, Bethlehem, United States; Montclair State University, Montclair, United States; Biology, University of Texas at El Paso, El Paso, United States; Biology, University of Texas at El Paso, El Paso, United States; Biology, Gonzaga University, Spokane, United States; Biology, Calvin College, Grand Rapids, United States; ISBT, LaSalle University, Philadelphia, United States; Biology, Calvin College, Grand Rapids, United States; University of Florida, Gainsville, United States; Biology, University of California San Diego, La Jolla, United States; University of Colorado at Boulder, Boulder, United States; Biology, Carthage College, Kenosha, United States; Biology, College of St. Scholastica, Duluth, United States; Biology, Hope College, Holland, United States; Department of Biology, Bucknell University, Lewisburg, United States; University of California Santa Cruz, Santa Cruz, United States; University of Colorado at Boulder, Boulder, United States; Department of Microbiology, Immunology, and Molecular Genetics, University of California, Los Angeles, Los Angeles, United States; Microbiology and Biotechnology, North Carolina State University, Raleigh, United States; University of Florida, Gainsville, United States; Biology, Gettysburg College, Gettysburg, United States; Biology, University of Texas at El Paso, El Paso, United States; University of California Santa Cruz, Santa Cruz, United States; Biological Sciences and Geology, Queensboro Community College, Bayside, United States; Montclair State University, Montclair, United States; Biology, University of Puerto Rico - Cayey, Cayey, United States; Biological Sciences, University of North Texas, Denton, United States; Department of Microbiology, Immunology, and Molecular Genetics, University of California, Los Angeles, Los Angeles, United States; University of California Santa Cruz, Santa Cruz, United States; Molecular and Cell Biology Program, Oregon State University, Corvallis, United States; Biology, Gonzaga University, Spokane, United States; Biology, Loyola Marymount University, Los Angeles, United States; Biological Sciences, University of Pittsburgh, Pittsburgh, United States; Biology, Hope College, Holland, United States; Biological Sciences, University of North Texas, Denton, United States; Biological Sciences, University of North Texas, Denton, United States; Department of Microbiology, Immunology, and Molecular Genetics, University of California, Los Angeles, Los Angeles, United States; Biology, University of Louisiana at Monroe, Monroe, United States; Natural Sciences, Del Mar College, Corpus Christi, United States; University of California Santa Cruz, Santa Cruz, United States; Virginia Commonwealth University, Richmond, United States; Biology, Saint Joseph's University, Philadelphia, United States; Xavier University of Louisiana, New Orleans, United States; Purdue University, West Lafayette, United States; Ohio State University, Columbus, United States; Microbiology, Miami University, Oxford, United States; Biology, College of St. Scholastica, Duluth, United States; Biological Sciences, University of Pittsburgh, Pittsburgh, United States; Western Kentucky University, Bowling Green, United States; Western Kentucky University, Bowling Green, United States; The Evergreen State College, Olympia, United States; Biology, Illinois Wesleyan University, Bloomington, United States; Microbiology, Miami University, Oxford, United States; Biology, Illinois Wesleyan University, Bloomington, United States; Biology, Saint Joseph's University, Philadelphia, United States; Biology, University of California San Diego, La Jolla, United States; Virginia Commonwealth University, Richmond, United States; Honors Program, Florida Gulf Coast University, Fort Myers, United States; Biology, Ouachita Baptist University, Arkadelphia, United States; Biology, University of California San Diego, La Jolla, United States; Biology, Illinois Wesleyan University, Bloomington, United States; University of Florida, Gainsville, United States; Biology, Washington University in St. Louis, St. Louis, United States; Virginia Commonwealth University, Richmond, United States; Biology, Washington University in St. Louis, St. Louis, United States; Biological Sciences, Lehigh University, Bethlehem, United States; Biology, University of Louisiana at Monroe, Monroe, United States; Biology and Medicine, Brown University, Providence, United States; Ohio State University, Columbus, United States; Biology and Chemistry, Nyack College, Nyack, United States; Purdue University, West Lafayette, United States; Biological Sciences, University of North Texas, Denton, United States; Biology, Washington University in St. Louis, St. Louis, United States; Division of Natural and Health Sciences, Seton Hill University, Greensburg, United States; Western Kentucky University, Bowling Green, United States; Biology, Culver-Stockton College, Canton, United States; biology, North Carolina Central University, Durham, United States; Ohio State University, Columbus, United States; Biology, Washington University in St. Louis, St. Louis, United States; University of California Santa Cruz, Santa Cruz, United States; Biology, Washington University in St. Louis, St. Louis, United States; Ohio State University, Columbus, United States; Biology, Spelman College, Atlanta, United States; Biology, Hope College, Holland, United States; Purdue University, West Lafayette, United States; Biology, Calvin College, Grand Rapids, United States; ISBT, LaSalle University, Philadelphia, United States; Biology, College of St. Scholastica, Duluth, United States; University of Florida, Gainsville, United States; Biology, Washington University in St. Louis, St. Louis, United States; Biology, Calvin College, Grand Rapids, United States; Biology, Trinity College, Hartford, United States; Providence College, Providence, United States; Biology, Washington University in St. Louis, St. Louis, United States; University of Maine, Honors College, Orono, United States; Biology, Gonzaga University, Spokane, United States; Department of Biology, Baylor University, Waco, United States; biology, North Carolina Central University, Durham, United States; Department of Biology, Baylor University, Waco, United States; Biological Sciences, University of North Texas, Denton, United States; Biology, Illinois Wesleyan University, Bloomington, United States; Purdue University, West Lafayette, United States; Biology, Calvin College, Grand Rapids, United States; Biology, University of Louisiana at Monroe, Monroe, United States; Biological Sciences, Carnegie Mellon University, Pittsburgh, United States; ISBT, LaSalle University, Philadelphia, United States; Biology, Loyola Marymount University, Los Angeles, United States; Biology, Gonzaga University, Spokane, United States; Biology, Gonzaga University, Spokane, United States; Washington State University, Pullman, United States; Biology, Culver-Stockton College, Canton, United States; Biology, University of Louisiana at Monroe, Monroe, United States; Biological Sciences, University of Pittsburgh, Pittsburgh, United States; Biological Sciences, University of North Texas, Denton, United States; Morehouse College, Atlanta, United States; biology, North Carolina Central University, Durham, United States; Biology, Spelman College, Atlanta, United States; Department of Microbiology, Immunology, and Molecular Genetics, University of California, Los Angeles, Los Angeles, United States; Biology, Loyola Marymount University, Los Angeles, United States; Biological Sciences, Carnegie Mellon University, Pittsburgh, United States; University of Colorado at Boulder, Boulder, United States; Morehouse College, Atlanta, United States; Environmental and Biological Science, University of Maine, Machias, Machias, United States; Biology, Ouachita Baptist University, Arkadelphia, United States; Biology, College of William and Mary, Williamsburg, United States; Virginia Commonwealth University, Richmond, United States; Biology, Ouachita Baptist University, Arkadelphia, United States; Biological Sciences and Geology, Queensboro Community College, Bayside, United States; Department of Biology, Baylor University, Waco, United States; Department of Biology, Baylor University, Waco, United States; Biology, Gonzaga University, Spokane, United States; Environmental and Biological Science, University of Maine, Machias, Machias, United States; Biology, College of Idaho, Caldwell, United States; Biology, Gettysburg College, Gettysburg, United States; Biology, University of Louisiana at Monroe, Monroe, United States; Biology, University of Alabama Birmingham, Birmingham, United States; Ohio State University, Columbus, United States; Biology, Gonzaga University, Spokane, United States; Biology, Smith College, Northampton, United States; Biology, University of California San Diego, La Jolla, United States; Biological Sciences, Carnegie Mellon University, Pittsburgh, United States; Morehouse College, Atlanta, United States; Biology, Washington University in St. Louis, St. Louis, United States; Science, Cabrini College, Radnor, United States; Microbiology and Biotechnology, North Carolina State University, Raleigh, United States; Biology, Smith College, Northampton, United States; Microbiology and Biotechnology, North Carolina State University, Raleigh, United States; Biology, Loyola Marymount University, Los Angeles, United States; Biology, Hampden-Sydney College, Farmville, United States; Biology, University of California San Diego, La Jolla, United States; Biology, Loyola Marymount University, Los Angeles, United States; University of Maine, Honors College, Orono, United States; Biology and Chemistry, Nyack College, Nyack, United States; Microbiology, Miami University, Oxford, United States; Morehouse College, Atlanta, United States; Washington State University, Pullman, United States; Biological Sciences, Carnegie Mellon University, Pittsburgh, United States; Microbiology, Miami University, Oxford, United States; Biology, Smith College, Northampton, United States; Honors Program, Florida Gulf Coast University, Fort Myers, United States; Biology, Smith College, Northampton, United States; Microbiology and Molecular Biology, Brigham Young University, Provo, United States; Biology, University of Texas at El Paso, El Paso, United States; Biology, College of Charleston, Charleston, United States; Western Kentucky University, Bowling Green, United States; Biology, University of Louisiana at Monroe, Monroe, United States; Biology, Calvin College, Grand Rapids, United States; Biology, University of California San Diego, La Jolla, United States; Department of Biology, Baylor University, Waco, United States; Biology, University of California San Diego, La Jolla, United States; Biological Sciences and Geology, Queensboro Community College, Bayside, United States; Purdue University, West Lafayette, United States; Biology, University of California San Diego, La Jolla, United States; Biology, Nebraska Wesleyan University, Lincoln, Nebraska, United States; biology, North Carolina Central University, Durham, United States; Biology, Loyola Marymount University, Los Angeles, United States; Biological Sciences, Lehigh University, Bethlehem, United States; Biology, Saint Joseph's University, Philadelphia, United States; Biological Sciences, University of Pittsburgh, Pittsburgh, United States; Biology, Gonzaga University, Spokane, United States; University of Florida, Gainsville, United States; Biology, University of California San Diego, La Jolla, United States; Department of Microbiology, Immunology, and Molecular Genetics, University of California, Los Angeles, Los Angeles, United States; University of Colorado at Boulder, Boulder, United States; Department of Microbiology, Immunology, and Molecular Genetics, University of California, Los Angeles, Los Angeles, United States; Microbiology and Biotechnology, North Carolina State University, Raleigh, United States; Biological Sciences and Geology, Queensboro Community College, Bayside, United States; Department of Biology, Baylor University, Waco, United States; University of Colorado at Boulder, Boulder, United States; Biology and Medicine, Brown University, Providence, United States; University of California Santa Cruz, Santa Cruz, United States; Biology, Gonzaga University, Spokane, United States; Biology, University of Texas at El Paso, El Paso, United States; Montclair State University, Montclair, United States; Biological Sciences, University of Pittsburgh, Pittsburgh, United States; Montclair State University, Montclair, United States; Biology, Smith College, Northampton, United States; Honors Program, Florida Gulf Coast University, Fort Myers, United States; Biology, University of Texas at El Paso, El Paso, United States; Biology, Gonzaga University, Spokane, United States; Biology, Saint Joseph's University, Philadelphia, United States; Biology, University of California San Diego, La Jolla, United States; University of Florida, Gainsville, United States; Department of Microbiology, Immunology, and Molecular Genetics, University of California, Los Angeles, Los Angeles, United States; Biological Sciences, University of Pittsburgh, Pittsburgh, United States; Biology, Calvin College, Grand Rapids, United States; Biology, Washington University in St. Louis, St. Louis, United States; Biology, Washington University in St. Louis, St. Louis, United States; Microbiology and Biotechnology, North Carolina State University, Raleigh, United States; Virginia Commonwealth University, Richmond, United States; Biological Sciences, Carnegie Mellon University, Pittsburgh, United States; Biology, University of California San Diego, La Jolla, United States; Biology, Loyola Marymount University, Los Angeles, United States; Biology, Illinois Wesleyan University, Bloomington, United States; ISBT, LaSalle University, Philadelphia, United States; Xavier University of Louisiana, New Orleans, United States; Environmental and Biological Science, University of Maine, Machias, Machias, United States; Environmental and Biological Science, University of Maine, Machias, Machias, United States; University of Colorado at Boulder, Boulder, United States; Biology, University of Wisconsin-River Falls, River Falls, United States; Biology, Gettysburg College, Gettysburg, United States; Biology, University of California San Diego, La Jolla, United States; Biology, Washington University in St. Louis, St. Louis, United States; Biology, University of California San Diego, La Jolla, United States; Natural Sciences, Del Mar College, Corpus Christi, United States; Biology, Washington University in St. Louis, St. Louis, United States; Biology, University of California San Diego, La Jolla, United States; Department of Microbiology, Immunology, and Molecular Genetics, University of California, Los Angeles, Los Angeles, United States; Biology, University of California San Diego, La Jolla, United States; Biology, Washington University in St. Louis, St. Louis, United States; Biology, Illinois Wesleyan University, Bloomington, United States; Biology, College of Charleston, Charleston, United States; University of Colorado at Boulder, Boulder, United States; Biology, Nebraska Wesleyan University, Lincoln, Nebraska, United States; Biology, Spelman College, Atlanta, United States; Department of Biological Sciences, University of Pittsburgh, Pittsburgh, United States; Department of Biological Sciences, University of Pittsburgh, Pittsburgh, United States; Department of Biological Sciences, University of Pittsburgh, Pittsburgh, United States; Department of Biological Sciences, University of Pittsburgh, Pittsburgh, United States; Department of Biological Sciences, University of Pittsburgh, Pittsburgh, United States; Department of Biological Sciences, University of Pittsburgh, Pittsburgh, United States; Department of Biological Sciences, University of Pittsburgh, Pittsburgh, United States; Department of Biological Sciences, University of Pittsburgh, Pittsburgh, United States; Department of Biological Sciences, University of Pittsburgh, Pittsburgh, United States; Department of Biological Sciences, University of Pittsburgh, Pittsburgh, United States; Department of Biological Sciences, University of Pittsburgh, Pittsburgh, United States; Department of Biological Sciences, University of Pittsburgh, Pittsburgh, United States; Department of Biological Sciences, University of Pittsburgh, Pittsburgh, United States; Department of Biological Sciences, University of Pittsburgh, Pittsburgh, United States; Department of Biological Sciences, University of Pittsburgh, Pittsburgh, United States; Department of Biological Sciences, University of Pittsburgh, Pittsburgh, United States; Department of Biological Sciences, University of Pittsburgh, Pittsburgh, United States; Department of Biological Sciences, University of Pittsburgh, Pittsburgh, United States; Department of Biological Sciences, University of Pittsburgh, Pittsburgh, United States; Department of Biological Sciences, University of Pittsburgh, Pittsburgh, United States; Department of Biological Sciences, University of Pittsburgh, Pittsburgh, United States; Department of Biological Sciences, University of Pittsburgh, Pittsburgh, United States; Department of Biological Sciences, University of Pittsburgh, Pittsburgh, United States; Department of Biological Sciences, University of Pittsburgh, Pittsburgh, United States; Department of Biological Sciences, University of Pittsburgh, Pittsburgh, United States; Department of Biological Sciences, University of Pittsburgh, Pittsburgh, United States; Department of Biological Sciences, University of Pittsburgh, Pittsburgh, United States; Department of Biological Sciences, University of Pittsburgh, Pittsburgh, United States; Department of Biological Sciences, University of Pittsburgh, Pittsburgh, United States; Department of Biological Sciences, University of Pittsburgh, Pittsburgh, United States; Department of Biological Sciences, University of Pittsburgh, Pittsburgh, United States; Department of Biological Sciences, University of Pittsburgh, Pittsburgh, United States; Department of Biological Sciences, University of Pittsburgh, Pittsburgh, United States; Department of Biological Sciences, University of Pittsburgh, Pittsburgh, United States; Department of Biological Sciences, University of Pittsburgh, Pittsburgh, United States; Department of Biological Sciences, University of Pittsburgh, Pittsburgh, United States; Department of Biological Sciences, University of Pittsburgh, Pittsburgh, United States; Department of Biological Sciences, University of Pittsburgh, Pittsburgh, United States; Department of Biological Sciences, University of Pittsburgh, Pittsburgh, United States; Department of Biological Sciences, University of Pittsburgh, Pittsburgh, United States; Department of Biological Sciences, University of Pittsburgh, Pittsburgh, United States; Department of Biological Sciences, University of Pittsburgh, Pittsburgh, United States; Department of Biological Sciences, University of Pittsburgh, Pittsburgh, United States; Department of Biological Sciences, University of Pittsburgh, Pittsburgh, United States; Department of Biological Sciences, University of Pittsburgh, Pittsburgh, United States; Department of Biological Sciences, University of Pittsburgh, Pittsburgh, United States; Department of Biological Sciences, University of Pittsburgh, Pittsburgh, United States; Department of Biological Sciences, University of Pittsburgh, Pittsburgh, United States; Department of Biological Sciences, University of Pittsburgh, Pittsburgh, United States; Department of Biological Sciences, University of Pittsburgh, Pittsburgh, United States; University of KwaZulu-Natal, Durban, South Africa; University of KwaZulu-Natal, Durban, South Africa; University of KwaZulu-Natal, Durban, South Africa; University of KwaZulu-Natal, Durban, South Africa; University of KwaZulu-Natal, Durban, South Africa; University of KwaZulu-Natal, Durban, South Africa; University of KwaZulu-Natal, Durban, South Africa; University of KwaZulu-Natal, Durban, South Africa; University of KwaZulu-Natal, Durban, South Africa; University of KwaZulu-Natal, Durban, South Africa; University of KwaZulu-Natal, Durban, South Africa; University of KwaZulu-Natal, Durban, South Africa; University of KwaZulu-Natal, Durban, South Africa; University of KwaZulu-Natal, Durban, South Africa; Microbiology and Immunology, Albert Einstein College of Medicine, Bronx, United States; University of KwaZulu-Natal, Durban, South Africa; University of KwaZulu-Natal, Durban, South Africa; University of KwaZulu-Natal, Durban, South Africa; University of KwaZulu-Natal, Durban, South Africa; University of KwaZulu-Natal, Durban, South Africa; University of KwaZulu-Natal, Durban, South Africa; University of KwaZulu-Natal, Durban, South Africa; University of KwaZulu-Natal, Durban, South Africa; University of KwaZulu-Natal, Durban, South Africa; University of KwaZulu-Natal, Durban, South Africa; University of KwaZulu-Natal, Durban, South Africa; University of KwaZulu-Natal, Durban, South Africa; University of KwaZulu-Natal, Durban, South Africa; University of KwaZulu-Natal, Durban, South Africa; University of KwaZulu-Natal, Durban, South Africa; University of KwaZulu-Natal, Durban, South Africa; University of KwaZulu-Natal, Durban, South Africa; University of KwaZulu-Natal, Durban, South Africa; University of KwaZulu-Natal, Durban, South Africa; University of KwaZulu-Natal, Durban, South Africa; University of KwaZulu-Natal, Durban, South Africa; Department of Immunology and Infectious Diseases, Harvard School of Public Health, United States; University of KwaZulu-Natal, Durban, South Africa; University of KwaZulu-Natal, Durban, South Africa; University of KwaZulu-Natal, Durban, South Africa; University of KwaZulu-Natal, Durban, South Africa; University of KwaZulu-Natal, Durban, South Africa; 1 Department of Biological Sciences, University of Pittsburgh, Pittsburgh, United States; 2 Howard Hughes Medical Institute, Chevy Chase, United States; 3 Department of Biology, James Madison University, Harrisonburg, United States; 4 Department of Microbiology and Immunology, Albert Einstein College of Medicine, Bronx, United States; Harvard Medical School, United States

**Keywords:** bacteriophage, genomic, evolution, Virus

## Abstract

The bacteriophage population is large, dynamic, ancient, and genetically diverse. Limited genomic information shows that phage genomes are mosaic, and the genetic architecture of phage populations remains ill-defined. To understand the population structure of phages infecting a single host strain, we isolated, sequenced, and compared 627 phages of *Mycobacterium smegmatis*. Their genetic diversity is considerable, and there are 28 distinct genomic types (clusters) with related nucleotide sequences. However, amino acid sequence comparisons show pervasive genomic mosaicism, and quantification of inter-cluster and intra-cluster relatedness reveals a continuum of genetic diversity, albeit with uneven representation of different phages. Furthermore, rarefaction analysis shows that the mycobacteriophage population is not closed, and there is a constant influx of genes from other sources. Phage isolation and analysis was performed by a large consortium of academic institutions, illustrating the substantial benefits of a disseminated, structured program involving large numbers of freshman undergraduates in scientific discovery.

**DOI:** http://dx.doi.org/10.7554/eLife.06416.001

## eLife digest

Viruses are unable to replicate independently. To generate copies of itself, a virus must instead invade a target cell and commandeer that cell's replication machinery. Different viruses are able to invade different types of cell, and a group of viruses known as bacteriophages (or phages for short) replicate within bacteria. The enormous number and diversity of phages in the world means that they play an important role in virtually every ecosystem.

Despite their importance, relatively little is known about how different phage populations are related to each other and how they evolved. Many phages contain their genetic information in the form of strands of DNA. Using genetic sequencing to find out where and how different genes are encoded in the DNA can reveal information about how different viruses are related to each other. These relationships are particularly complicated in phages, as they can exchange genes with other viruses and microbes.

Previous studies comparing the genomes—the complete DNA sequence—of reasonably small numbers of phages that infect the *Mycobacterium* group of bacteria have found that the phages can be sorted into ‘clusters’ based on similarities in their genes and where these are encoded in their DNA. However, the number of phages investigated so far has been too small to conclude how different clusters are related. Are the clusters separate, or do they form a ‘continuum’ with different genes and DNA sequences shared between different clusters?

Here, Pope, Bowman, Russell et al. compare the individual genomes of 627 bacteriophages that infect the bacterial species *Mycobacterium smegmatis*. This is by far the largest number of phage genomes analyzed from a single host species. The large number of genomes analyzed allowed a much clearer understanding of the complexity and diversity of these phages to be obtained. The isolation, sequencing and analysis of the hundreds of *M. smegmatis* bacteriophage genomes was performed by an integrated research and education program, called the Science Education Alliance Phage Hunters Advancing Genomics and Evolutionary Science (SEA-PHAGES) program. This enabled thousands of undergraduate students from different institutions to contribute to the phage discovery and sequencing project, and co-author the report. SEA-PHAGES therefore shows that it is possible to successfully incorporate genuine scientific research into an undergraduate course, and that doing so can benefit both the students and researchers involved.

The results show that while the genomes could be categorized into 28 clusters, the genomes are not completely unrelated. Instead, a spread of diversity is seen, as genes and groups of genes are shared between different clusters. Pope, Bowman, Russell et al. further reveal that the phage population is in a constant state of change, and continuously acquires genes from other microorganisms and viruses.

**DOI:** http://dx.doi.org/10.7554/eLife.06416.002

## Introduction

Bacteriophages are the dark matter of the biological universe, forming a vast, ancient, dynamic, and genetically diverse population, replete with genes of unknown function (Pedulla et al., 2003). Phages are the most abundant organisms in the biosphere, and the ∼10^31^ tailed phage particles participate in ∼10^23^ infections per second on a global scale, with the entire population turning over every few days (Suttle, 2007). The population is not only vast and dynamic, but comparisons of virion structures suggest that it is also extremely old (Krupovic and Bamford, 2010). It is thus not surprising that bacteriophages are genetically highly diverse, although their comparative genomics has lagged behind that of other microbes, largely due to the lack of individual isolates for genomic analyses (Hatfull and Hendrix, 2011). To date, there are approximately 2000 completely sequenced bacteriophage genomes in the GenBank database, a small number relative to the more than 30,000 sequenced prokaryotic genomes (http://www.ncbi.nlm.nih.gov/genome/browse/), in spite of phage genomes being only 1–5% of the size of their host genomes.

Double-stranded DNA tailed phages are proposed to have evolved with common ancestry but with different phages having differential access to a large common gene pool (Hendrix et al., 1999). Phage genomes are typified by their mosaic architectures generated by gene loss and gain through horizontal genetic exchange; however, the parameters influencing access to the common gene pool are numerous and likely include host range, genome size, replication mode, and life style (temperate vs lytic). Migration to new hosts is probably common, but is affected by local host diversity and mutation rates, as well as resistance mechanisms such as receptor availability, restriction, CRISPRs, and abortive infection systems (Buckling and Brockhurst, 2012; Jacobs-Sera et al., 2012; Hoskisson et al., 2015). Constraints on gene acquisition may also be imposed by synteny—particularly among virion structural genes—and by size limits of DNA packaging (Juhala et al., 2000; Hatfull and Hendrix, 2011).

We have previously described comparative analyses of modest numbers of mycobacteriophages and shown that they can be sorted by nucleotide sequence and gene content comparisons into groups of closely related genomes referred to as ‘clusters’ (designated Cluster A, B, C, etc.); phages without any close relatives are referred to as ‘singletons’. Some of the clusters can be further divided into subclusters (e.g., Subcluster A1, A2, A3, etc.) according to nucleotide sequence relatedness (Pedulla et al., 2003; Hatfull et al., 2006, 2010; Pope et al., 2011b). The genomes are mosaic whereby individual phages are constructed as assemblages of modules, many of which are single genes (Pedulla et al., 2003). Each mycobacteriophage cluster has features particular to that cluster (e.g., regulatory systems, repeated sequences, tRNA genes, etc. [Pope et al., 2011a, 2011b, 2013, 2014a, 2014b]), but because of the pervasive mosaicism, the relationships among phages within clusters and between clusters are complex. Collections of phages have been isolated on other hosts such as *Bacillus* spp., *Escherichia coli*, *Pseudomonas* spp., *Propionibacterium* spp. and *Staphylococcus* spp. (Kwan et al., 2005, 2006; Kropinski et al., 2007; Marinelli et al., 2012; Hatfull et al., 2013; Grose and Casjens, 2014; Grose et al., 2014; Lee et al., 2014) and these can be similarly divided into clusters based on DNA similarity. Recent analysis of 337 phages infecting 31 bacterial species within the *Enterobacteriaceae* (Grose and Casjens, 2014) reveals 56 clusters of phage genomes. It is thus clear that there is substantial diversity within the phage population, even when comparing phages of a common host and which are expected to be in direct genetic contact with each other in their natural environment (Hatfull and Hendrix, 2011). Nonetheless, the numbers of genomes isolated on a particular host generally are too small to define the nature and the size of the populations at large with any substantial resolution.

Viral metagenomic studies provide valuable insights into phage diversity and population dynamics, but typically generate few complete genome sequences or any specific information relating viral genomes to specific bacterial hosts (Hambly and Suttle, 2005; Rodriguez-Brito et al., 2010; Mokili et al., 2012). A recent analysis of *Synechococcus* phages using metagenomic analysis coupled with viral tagging showed that there are multiple ‘populations’ of these phages (similar to the clusters described above), but suggested that these represent distinct groups of related phages rather than a continuous spectrum of diversity (Deng et al., 2014). This differs from prior predictions that the phage population as a whole likely spans a continuum of diversity—albeit with uneven representation of different groups of related phages—because of genomic mosaicism (Hendrix, 2003; Hatfull, 2010, 2012). However, as the *Synechococcus* phage data are derived from a single sample using a single host, it is unclear if this extends to phages of other hosts (Deng et al., 2014).

Here we describe the comparative analysis of a large number of completely sequenced mycobacteriophage genomes and demonstrate that they represent a spectrum of diversity and do not constitute discrete populations. Rarefaction analyses of their constituent genes are consistent with populations of gene families shared among mycobacteriophages being augmented by the introduction of new gene families from outside sources. The assembling of a large and highly informative collection of bacteriophages by a consortium of students and faculty at multiple institutions demonstrates that a course-based research experience (CRE) can be successfully implemented at large scale without compromising the authenticity or richness of a scientific investigation imbued with discovery and project ownership.

## Results and discussion

### A genome-by-genome approach to defining phage diversity

Exploring phage diversity using a genome-by-genome approach has notable advantages and some potential disadvantages. The main advantage is that complete genome sequences give information about genome length and composition, providing key insights into genome mosaicism and how genome segments are shared and exchanged. A difficulty is that there are not large extant phage collections available for most bacterial hosts, and isolation, purification, and characterization of phages can be slow and time-consuming. Because isolation typically requires plaque formation and growth in the laboratory, some naturally occurring phages may escape isolation using standard methods. Thus, although the diversity of phages isolated and propagated in the laboratory may not capture all types of phage, it represents a minimum, not a maximum, index of diversity.

### Authentic research in a CRE

The 2012 report from the President's Council of Advisors on Science and Technology (PCAST) focused on the poor retention of undergraduate students in science, technology, engineering and mathematics (STEM) as an impediment to meeting US economic demands (PCAST, 2012). One of the PCAST recommendations is to replace traditional introductory laboratory courses with research-based experiences that would inspire freshman students and promote STEM retention. A powerful strategy is to engage students in scientific discovery through CREs. The successful implementation of this strategy depends on (i) identifying research questions that can engage students in contributing genuine advances in scientific knowledge without requiring prior expert knowledge, and (ii) designing the project so that large numbers of students can participate in a meaningful fashion.

We have previously described the Howard Hughes Medical Institute (HHMI) Science Education Alliance Phage Hunters Advancing Genomics and Evolutionary Science (SEA-PHAGES) program, in which beginning undergraduate students isolate, purify, sequence, annotate, and compare bacteriophages, and have described its educational advantages (Jordan et al., 2014). By taking advantage of the massive diversity of the phage population so that each student can isolate a unique phage, the program encourages student ownership of their science. And because the collective discoveries by many students generate new scientific insights, the program creates a scientific community of students engaged in authentic research.

**Figure 1.**
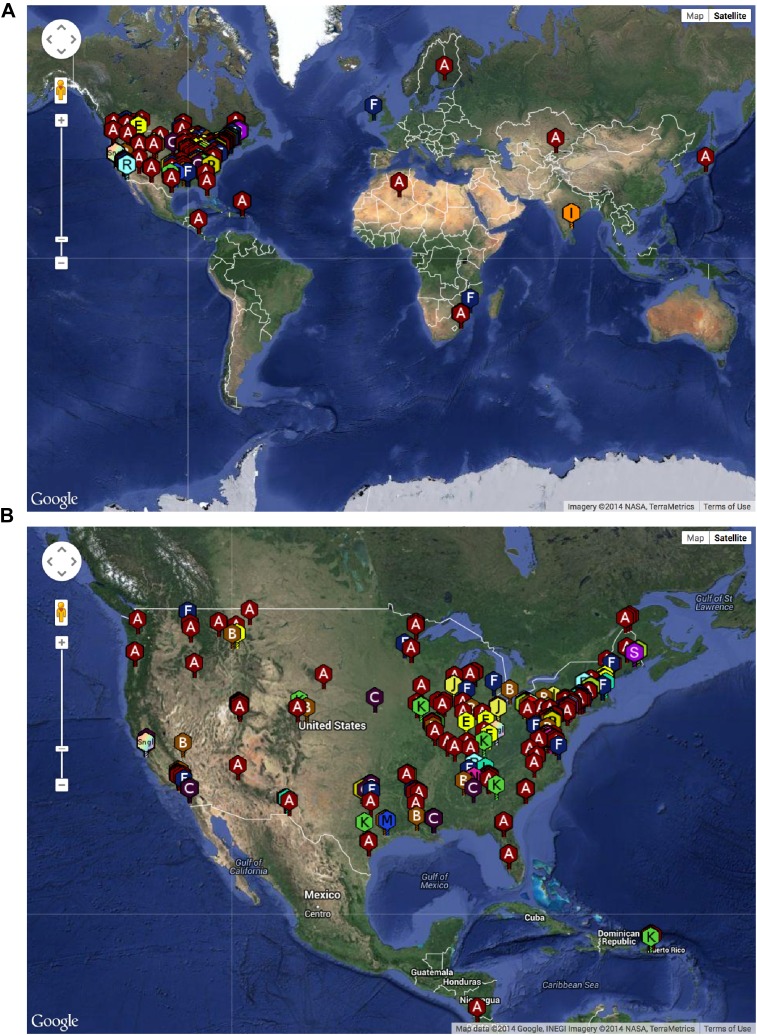
Geographical distribution of sequenced mycobacteriophages. (**A**) Locations of sequenced mycobacteriophages across the globe. (**B**) Locations of sequenced mycobacteriophages across the United States. Colors and letter designations on the isolates refer to the cluster to which the genomes belong. Data from www.phagesdb.org. **DOI:** http://dx.doi.org/10.7554/eLife.06416.003

The SEA-PHAGES program has contributed to the growth of the collection of sequenced mycobacteriophages to nearly 700 individual isolates (http://phagesdb.org), of which 627 were selected for a detailed analysis (Supplementary file 1). This is by far the largest collection of sequenced phage genomes for any single host and thus promises to substantially advance our understanding of phage diversity. The phages were isolated using either direct plating or by enrichment using *Mycobacterium smegmatis* mc^2^155 as a host, and sequenced using next-generation approaches (see ‘Materials and methods’). More than 5000 students—primarily freshmen—at 74 institutions have been involved since inception of the SEA-PHAGES program in 2008, and the phages isolated represent a broad geographical distribution (Figure 1) and a variety of viral morphotypes (http://phagesdb.org). The new insights gained from comparative genomic analyses of these phages—as described below—demonstrate the effectiveness of viral discovery and genomics as a model for CRE development.

### Assembling mycobacteriophages into clusters and subclusters

**Figure 2.**
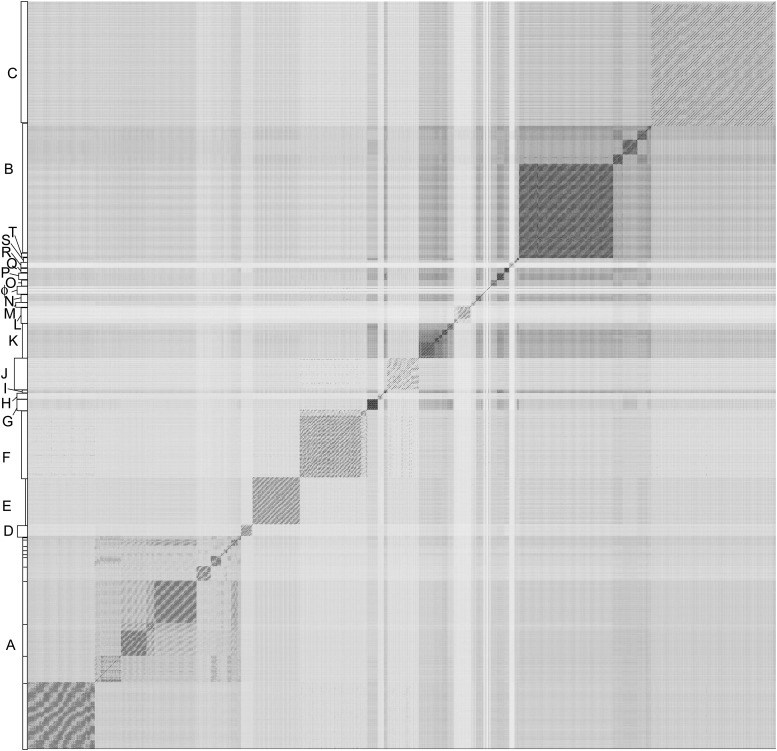
Nucleotide sequence comparison of 627 mycobacteriophages displayed as a dotplot. Complete genome sequences of 627 mycobacteriophages were concatenated into a single file which was compared with itself using Gepard (Krumsiek et al., 2007) and displayed as a dotplot using default parameters (word length, 10). The order of the genomes is as listed in Supplementary file 1. Nucleotide similarity is a primary component in assembling phages into clusters, which typically requires evident DNA similarity spanning more than 50% of the genome lengths. **DOI:** http://dx.doi.org/10.7554/eLife.06416.004 10.7554/eLife.06416.005Figure 2—source data 1.Concatenated DNA sequences for 627 phage genomes.**DOI:** http://dx.doi.org/10.7554/eLife.06416.005

Using previously reported parameters based primarily on nucleotide sequence similarity spanning >50% genome length (Hatfull et al., 2006), the 627 genomes were assembled into 20 clusters (A–T) and eight singletons (with no close relatives) (Figure 2, Supplementary file 1); 11 clusters were subdivided into 2 to 11 subclusters (Table 1). There is considerable variation in cluster size with substantial differences in the numbers of genomes in each cluster (2–232), but there is relatively little variation in either genome length or the numbers of genes per genome in any given cluster (Table 1). Cluster assignment is of practical utility and is generally robust, with clustered phages typically sharing genome architectures, as noted for the *Enterobacteriacea* (Grose and Casjens, 2014). For example, Cluster A phages are similar in size and transcriptional organization, and share an unusual immunity system (Brown et al., 1997; Pope et al., 2011b). Cluster M phages all contain large numbers of tRNA genes (Pope et al., 2014a), Cluster K (Pope et al., 2011a) and Cluster O (Cresawn et al., 2015) phages have different but characteristic repeated sequences, and Cluster J phages have an unusual capsid with a triangulation (T) number of 13 (Pope et al., 2013). Therefore, the organization of related mycobacteriophages into clusters provides a framework for identifying and interpreting gene trafficking within and among potentially distinct groups of genomes.

**Figure 2—figure supplement 1.**
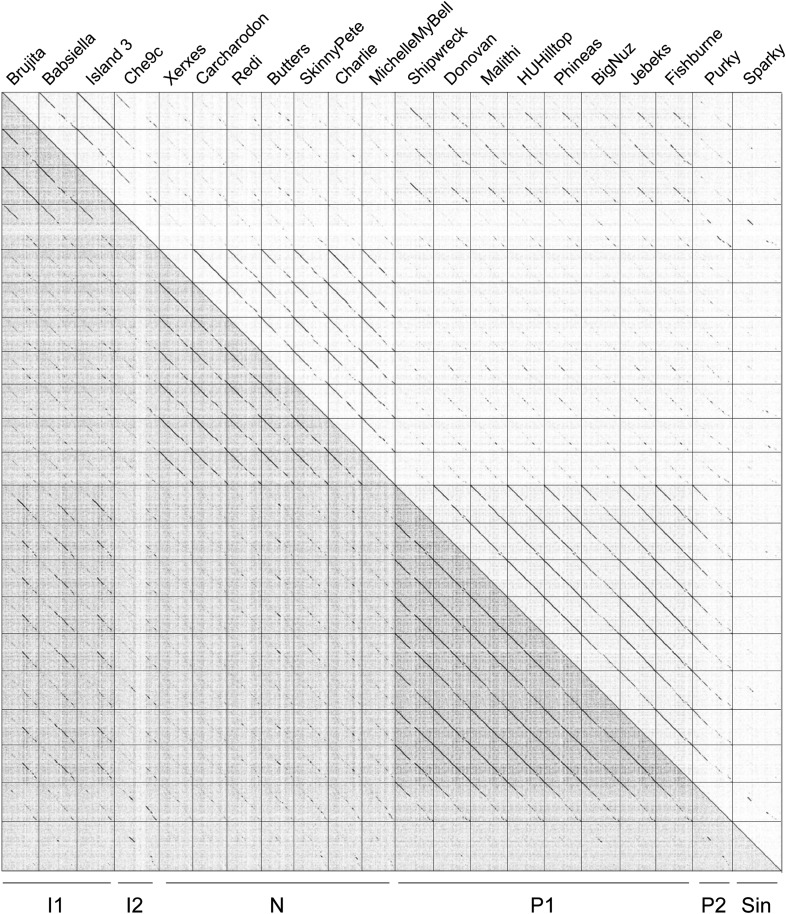
Dotplot of phages in Clusters I, N, P and the singleton Sparky. A dotplot was generated using a concatenated file of genome sequences using Gepard (Krumsiek et al., 2007). The complexity of the genome relationships is illustrated by the Cluster I phages which share varying degrees of similarity to phages in Clusters N and P, as well as the singleton Sparky. Because inclusion of a phage in a cluster typically requires sharing a span of similarity over half of the genome lengths, these phages are not assembled into a single larger cluster. **DOI:** http://dx.doi.org/10.7554/eLife.06416.006

**Figure 2—figure supplement 2.**
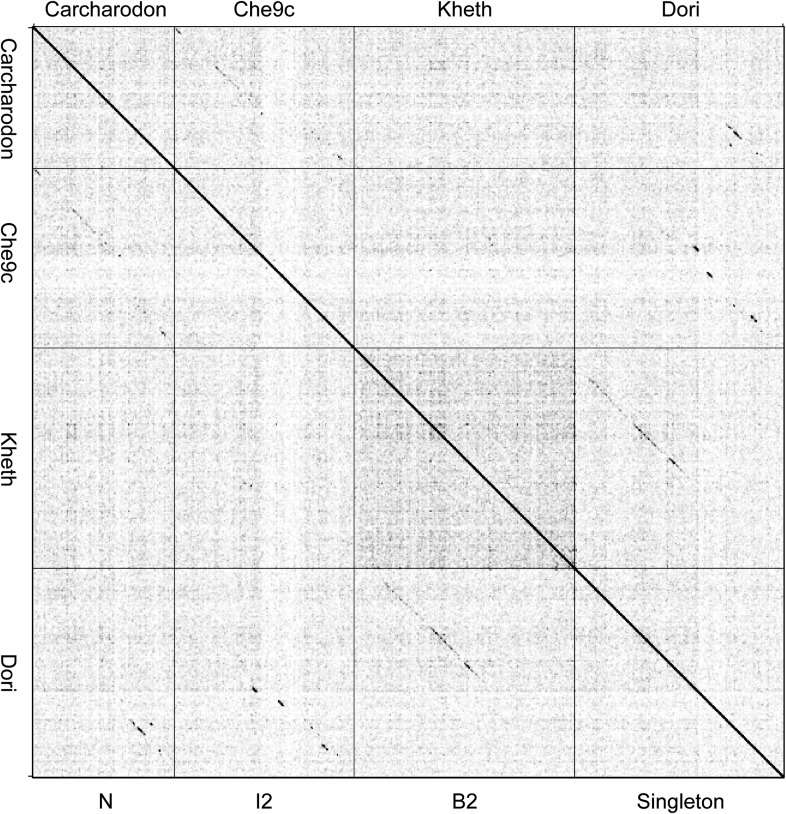
Dotplot of Carcharodon, Che9c, Kheth, and Dori. The dotplot of concatenated genome sequences illustrates the ambiguity of whether the singleton Dori warrants inclusion in Cluster B. Dori shares DNA sequence similarity with its closest relative Kheth (Subcluster B2), but it does not span 50% of the genome lengths. Dori also shares DNA sequence similarity with Che9c (Cluster I2) and Carcharodon (Cluster N). **DOI:** http://dx.doi.org/10.7554/eLife.06416.007

**Figure 2—figure supplement 3.**
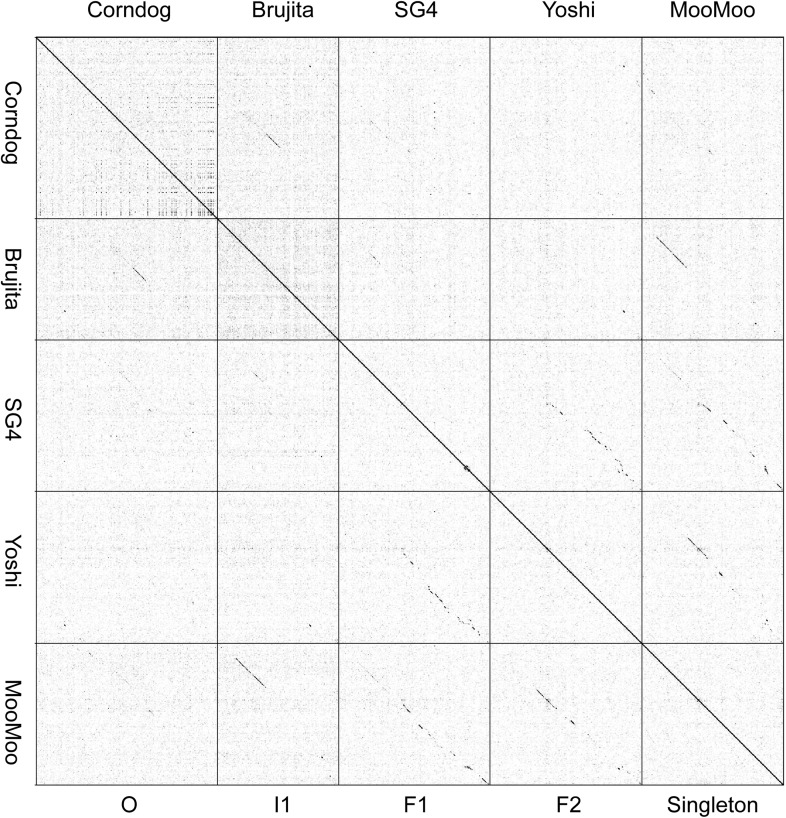
Dotplot of Corndog, Brujita, SG4, Yoshi, and MooMoo. The dotplot of concatenated genome sequences illustrates the complex relationships between the singleton MooMoo and other phages. MooMoo shares DNA sequence similarity with SG4 (Subcluster F1) and Yoshi (Subcluster F2), but also with Brujita (Subcluster I1). MooMoo has barely detectable DNA sequence similarity with Corndog (Cluster O), but has a similar prolate virion morphology. **DOI:** http://dx.doi.org/10.7554/eLife.06416.008

**Table 1.** Diversity and genetic isolation of mycobacteriophage genome clusters **DOI:** http://dx.doi.org/10.7554/eLife.06416.009

Cluster	# Subclusters	# Genomes	Average # genes*	Average length (bp)	Total phams†	Total genes	CLASP‡	CAP§	CCI#	CII¶
A	11	232	90 ± 5.3	51,514	1085	20,880	38.3	12.4	0.08	80.2
B	5	109	100.4 ± 4.5	68,653	421	10,944	66.2	23.2	0.24	81.0
C	2	45	231 ± 5.9	155,504	486	10,395	89.3	29.4	0.48	84.6
D	2	10	89.3 ± 6.4	64,965	147	893	88.1	64.3	0.61	71.4
E	1	35	141.9 ± 3.4	75,526	236	4967	87.2	63.8	0.60	59.3
F	3	66	105.3 ± 5.3	57,416	658	6950	54.4	4.9	0.16	55.8
G	1	14	61.5 ± 1.2	41,845	72	861	96.0	91.1	0.85	55.6
H	2	5	98.4 ± 5.7	69,469	207	492	61.6	31.5	0.48	67.6
I	2	4	78 ± 3.7	49,954	147	312	58.9	35.0	0.53	23.8
J	1	16	239.8 ± 9.3	110,332	530	3776	70.8	40.1	0.45	58.5
K	5	32	95.7 ± 4.6	59,720	411	3069	51.8	20.0	0.23	73.5
L	3	13	127.9 ± 6.5	75,177	246	1663	78.2	50.8	0.52	72.4
M	2	3	141 ± 8.8	81,636	201	423	73.5	63.0	0.70	69.2
N	1	7	69.1 ± 2.2	42,888	152	484	64.1	45.6	0.45	40.8
O	1	5	124.2 ± 3.1	70,651	151	621	90.6	83.3	0.82	64.2
P	2	9	78.8 ± 2.1	47,668	159	709	76.1	42.3	0.50	34.0
Q	1	5	85.2 ± 3.7	53,755	90	426	96.6	90.4	0.95	73.3
R	1	4	101.5 ± 2.5	71,348	117	406	91.4	84.8	0.87	71.8
S	1	2	109 ± 2.0	65,172	117	218	91.7	91.7	0.93	70.9
T	1	3	66.7 ± 2.4	42,833	83	200	86.1	82.5	0.80	62.7
Dori	1	1	94	64,613	94	94	N/A	N/A	N/A	35.8
DS6A	1	1	97	60,588	96	97	N/A	N/A	N/A	58.3
Gaia	1	1	194	90,460	193	194	N/A	N/A	N/A	58.0
MooMoo	1	1	98	55,178	98	98	N/A	N/A	N/A	31.6
Muddy	1	1	71	48,228	70	71	N/A	N/A	N/A	71.4
Patience	1	1	109	70,506	109	109	N/A	N/A	N/A	57.8
Sparky	1	1	93	63,334	93	93	N/A	N/A	N/A	48.4
Wildcat	1	1	148	78,296	148	148	N/A	N/A	N/A	69.6

* Average number of protein-coding genes per genome, with standard deviation.

† Total phams is the sum of all phamilies (groups of homologous mycobacteriophage genes) in that cluster.

‡ The Cluster Averaged Shared Phamilies (CLASP) index is the average of the percentages of phamilies shared pairwise between genomes within a cluster.

§ The Cluster-Associated Phamilies (CAP) index is the percentage of the average number of phamilies per genome within a cluster whose phamilies are present in every cluster member.

# The Cluster Cohesion Index (CCI) is generated by dividing the average number of genes per genome by the total number of phamilies (phams) in that cluster.

¶ The Cluster Isolation Index (CII) is the percentage of phams that are present only in that cluster, and not present in other mycobacteriophages.

N/A: Not applicable.

### Gene content relationships among sequenced mycobacteriophages

**Figure 3.**
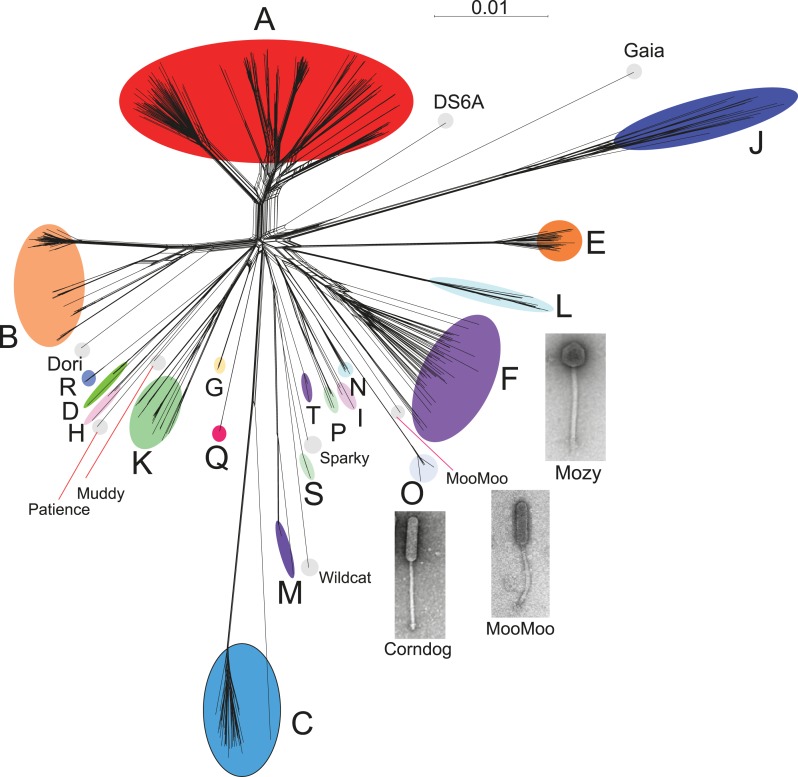
Network phylogeny of 627 mycobacteriophages based on gene content. Genomes of 627 mycobacteriophages were compared according to shared gene content using the Phamerator (Cresawn et al., 2011) database Mykobacteriophage_627, and displayed using SplitsTree (Huson and Bryant, 2006). Colored circles indicate grouping of phages labeled according to their cluster designations generated by nucleotide sequence comparison (Figure 2); singleton genomes with no close relatives are labeled but not circled. Micrographs show morphotypes of the singleton MooMoo, the Cluster F phage Mozy, and the Cluster O phage Corndog. With the exception of DS6A, all of the phages infect *Mycobacterium smegmatis* mc^2^155. **DOI:** http://dx.doi.org/10.7554/eLife.06416.010 10.7554/eLife.06416.011Figure 3—source data 1.Nexus file containing phamily assignments for 627 phage genomes.**DOI:** http://dx.doi.org/10.7554/eLife.06416.011

Genome mosaicism is more apparent from comparison of gene product amino acid sequences than nucleotide sequence comparisons because of the accumulation of genome rearrangements over a longer period of evolution, during which indications of DNA similarity are lost. To compare mycobacteriophage gene contents we grouped related genes into protein families (‘phamilies’ or ‘phams’) using Phamerator (Cresawn et al., 2011), which we modified to use kClust (Hauser et al., 2013) so as to easily accommodate the large numbers of comparisons. The 69,633 genes assembled into 5205 phams of which 1613 (31%) are orphams (single-gene phamilies [Hatfull et al., 2010]). Approximately 25% of phams can be assigned functions in viral structure and assembly, DNA metabolism, integration, lysis, and regulation, but the vast majority are of unknown function. Representation of gene content relationships among all 627 phages as a network phylogeny reveals relationships that are in accord with the cluster and subcluster designations derived from nucleotide sequence comparisons (Figure 3). The multiple branches between clusters/subclusters reflect the phylogenetic complexities that arise from genome mosaicism, where genes within a genome have distinct evolutionary histories.

**Figure 4.**
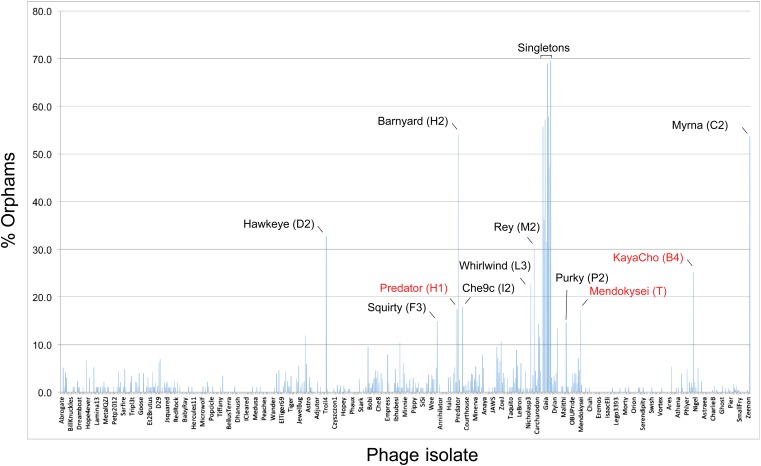
Proportions of orphams in mycobacteriophage genomes. The proportions of genes that are orphams (i.e., single-gene phamilies with no homologues within the mycobacteriophage dataset) are shown for each phage. The order of the phages is as shown in Supplementary file 1. All of the singleton genomes have >30% orphams, and most of the other genomes with relatively high proportions of orphams are the single-genome subclusters (Table 2) including Hawkeye (D2), Myrna (C2), Squirty (F3), Barnyard (H2), Che9c (I2), Whirlwind (L3), Rey (M2), and Purky (P2). Three phages shown in red type are not singletons or single-genome subclusters but have relatively high proportions of orphams. Predator and Mendokysei are members of the diverse and small clusters (five or fewer genomes) H and T, respectively; KayaCho is a member of Subcluster B4 but has a sufficiently high proportion of orphams to arguably warrant formation of a new subcluster, B6. **DOI:** http://dx.doi.org/10.7554/eLife.06416.012 10.7554/eLife.06416.013Figure 4—source data 1.Pham table containing phamily designations for 627 phage genomes.**DOI:** http://dx.doi.org/10.7554/eLife.06416.013

The distribution of orphams (genes without mycobacteriophage homologues) provides additional support for cluster/subcluster assignments; Figure 4). A relatively high proportion of orphams is a characteristic of both singleton genomes and single-genome subclusters (Figure 4). At least 30% of genes in all of the singleton genomes are orphams, and the single-genome subclusters have a minimum of 15% orphams; genomes in other clusters and subclusters typically have fewer than 10% orphams (Figure 4). The presence of numerous orphams ensures that the lack of cluster inclusion did not result from sequence errors or insufficient or inappropriate gene annotation. Notable exceptions are Predator (Subcluster H1) and Mendokysei (Cluster T), both of which are in very small clusters/subclusters, and KayaCho (Subcluster B4). KayaCho may warrant separation into a new subcluster (e.g., B6), but overall the orpham distribution is consistent with the cluster/subcluster designations.

**Figure 4—figure supplement 1.**
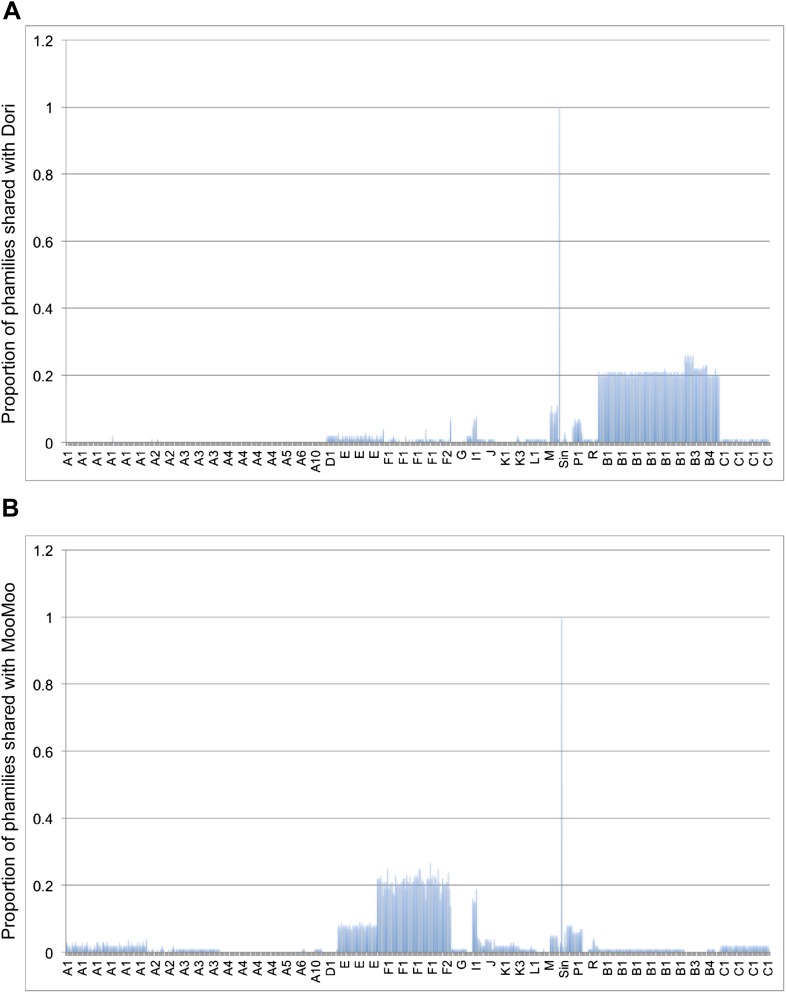
Shared gene content between Dori, MooMoo, and other mycobacteriophages. (**A**) Average percentages of phamilies shared between Dori and other mycobacteriophages. (**B**) Average percentages of phamilies shared between MooMoo and other mycobacteriophages. Genomes on the x axis are listed in the same order as in Supplementary file 1 and the cluster designations are indicated. **DOI:** http://dx.doi.org/10.7554/eLife.06416.014

**Figure 4—figure supplement 2.**
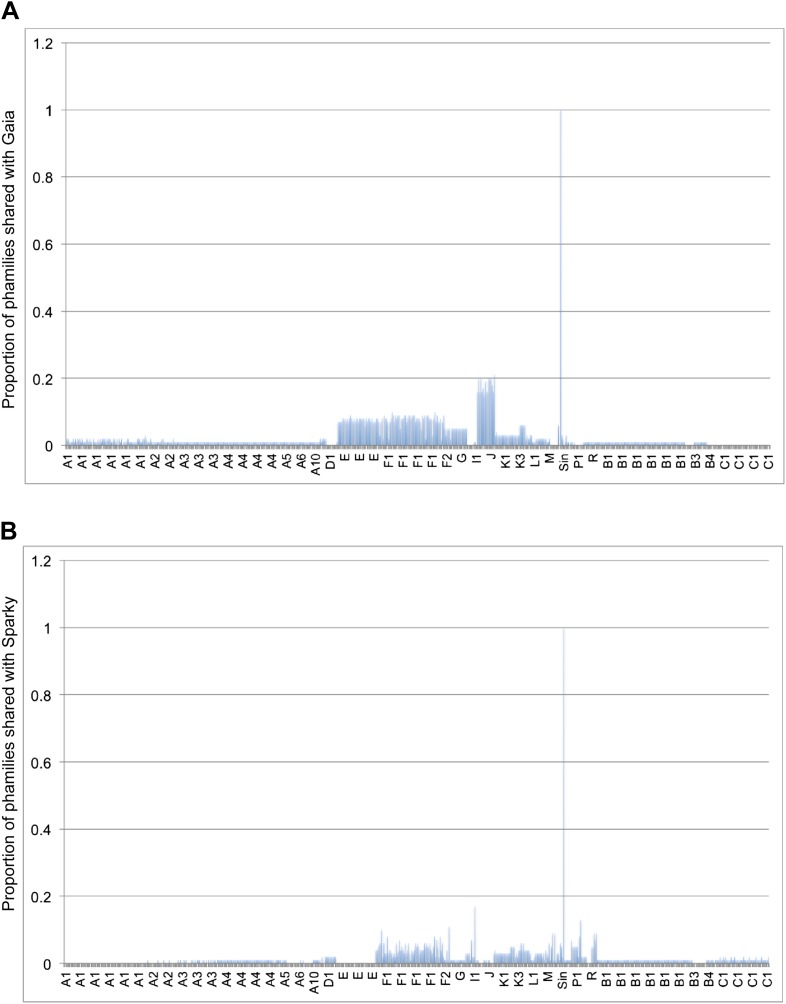
Shared gene content between Gaia, Sparky, and other mycobacteriophages. (**A**) Average percentages of phamilies shared between Gaia and other mycobacteriophages. (**B**) Average percentages of phamilies shared between Sparky and other mycobacteriophages. Genomes on the x axis are listed in the same order as in Supplementary file 1 and the cluster designations are indicated. **DOI:** http://dx.doi.org/10.7554/eLife.06416.015

### The diversity of different clusters is highly varied

**Figure 5.**
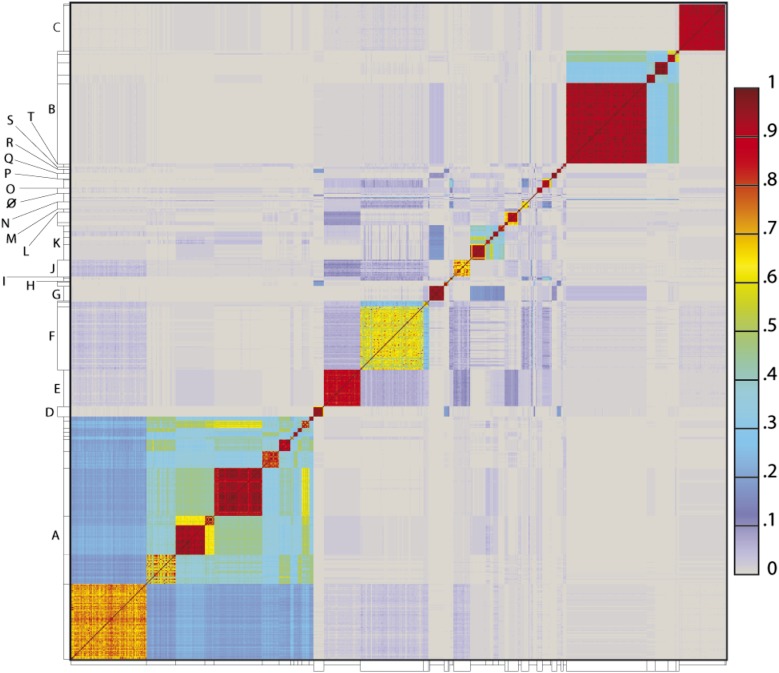
Heat map representation of shared gene content among 627 mycobacteriophages. The percentages of pairwise shared genes was determined using a Phamerator (Cresawn et al., 2011) database (Mykobacteriophage_627) populated with 627 completely sequenced phage genomes. The 69,574 genes were assembled into 5205 phamilies (phams) of related sequences using kClust, and the average proportions of shared phams calculated. Genomes are ordered on both axes according to their cluster and subcluster designations (Supplementary file 1) determined by nucleotide sequence similarities (Figure 2). The values (proportions of pairwise shared phams averaged between each partner) are colored as indicated. **DOI:** http://dx.doi.org/10.7554/eLife.06416.016 10.7554/eLife.06416.017Figure 5—source data 1.Dataset showing percentages of pairwise shared phamilies.**DOI:** http://dx.doi.org/10.7554/eLife.06416.017

**Figure 6.**
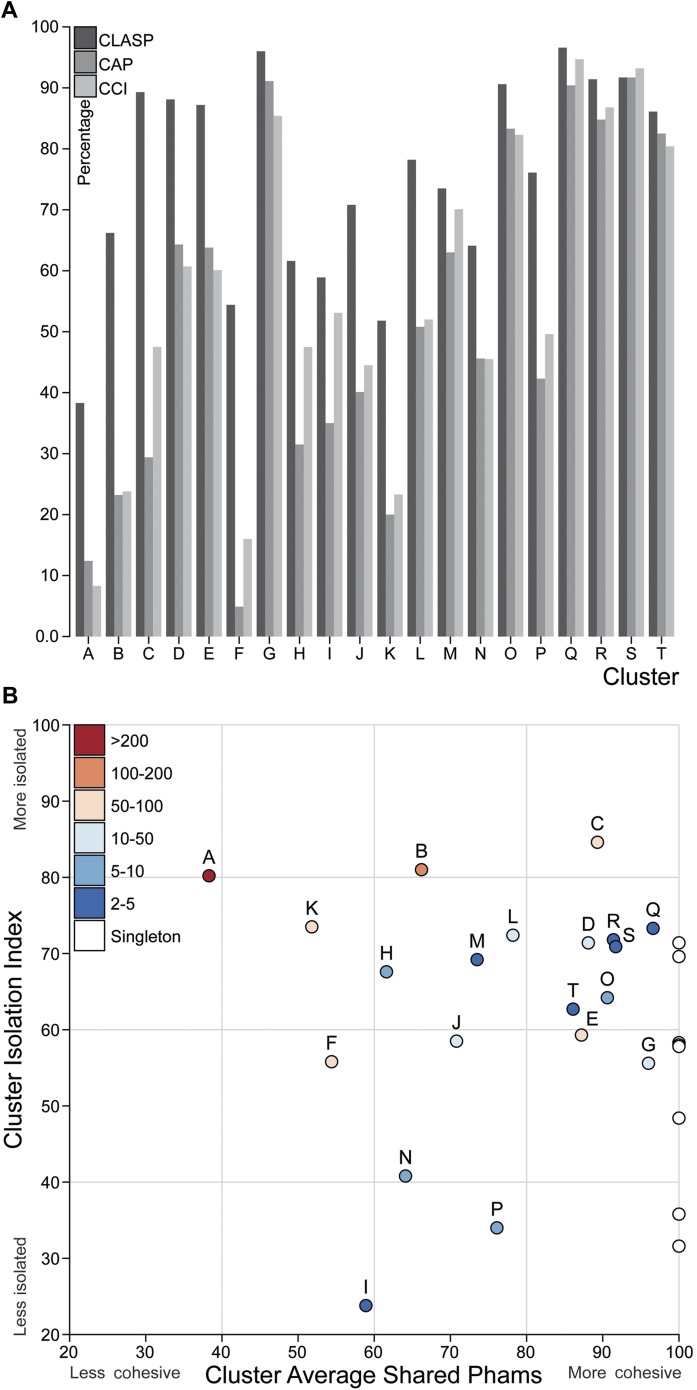
Cluster diversity and isolation. (**A**) The CLuster Averaged Shared Phamilies (CLASP; blue), Cluster Associated Phamilies (CAP; red) and Cluster Cohesion Index (CCI; green) values are plotted for each mycobacteriophage cluster. (**B**) The Cluster Isolation Index (CII) and CLASP values (both shown as percentages) are plotted for each phage cluster. Singletons (white circles) are not individually labeled but correspond to the values shown in Table 1. **DOI:** http://dx.doi.org/10.7554/eLife.06416.018 10.7554/eLife.06416.019Figure 6—source data 1.Datasets showing numbers of CLuster Average Shard Phamilies.**DOI:** http://dx.doi.org/10.7554/eLife.06416.019

To determine the extent to which the various clusters/subclusters represent discrete groups, we generated a heat map showing pairwise shared gene content (Figure 5) and quantified the cluster/subcluster diversity (Table 1, Figure 6). The heat map strikingly illustrates that diversity is non-uniform, with genomes in some clusters (e.g., Subclusters B1, C1) being very closely related, whereas in others they display substantial differences (e.g., Subclusters A1, F1). The variation is also evident within the large Cluster A group, with some subclusters having low diversity (e.g., A4, A5, A6), some being highly diverse (e.g., A1, A2), and some plausibly further splitting into subgroups (A3) (Figure 5).

**Figure 6—figure supplement 1.**
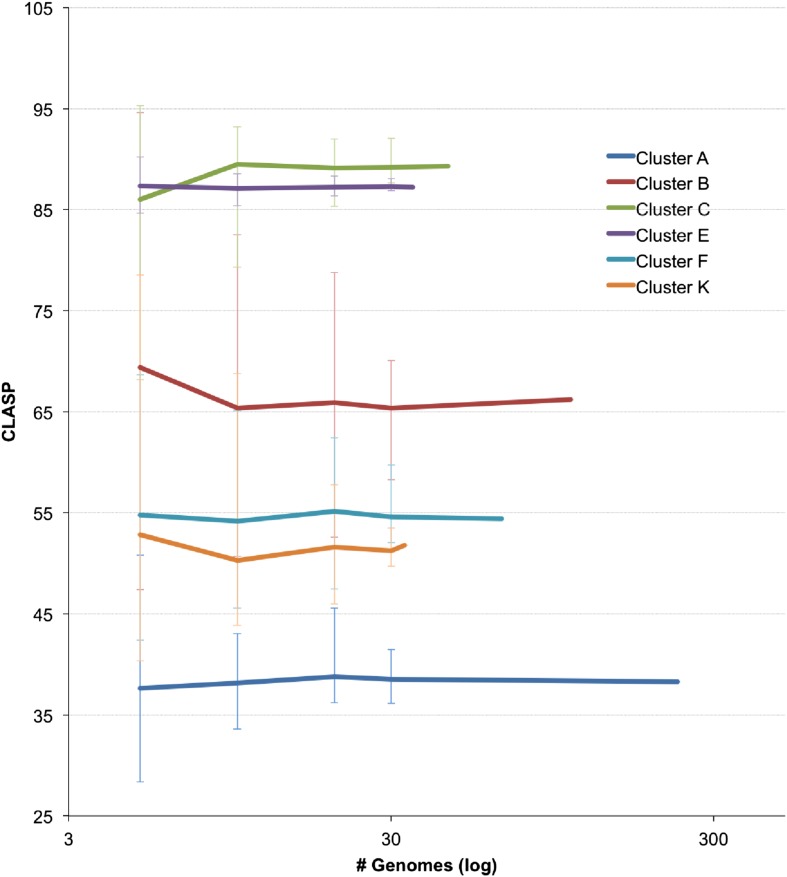
Resampling CLASP values for cluster diversity and size. CLuster Averaged Shared Phamilies (CLASP) values were calculated for Clusters A, B, C, E, F, and K by resampling random subsets of the genomes. The size of the subsets is shown on the x axis and each point is the average of 20 iterations. The minimum and maximum variations among the iterations are shown. **DOI:** http://dx.doi.org/10.7554/eLife.06416.020

**Figure 6—figure supplement 2.**
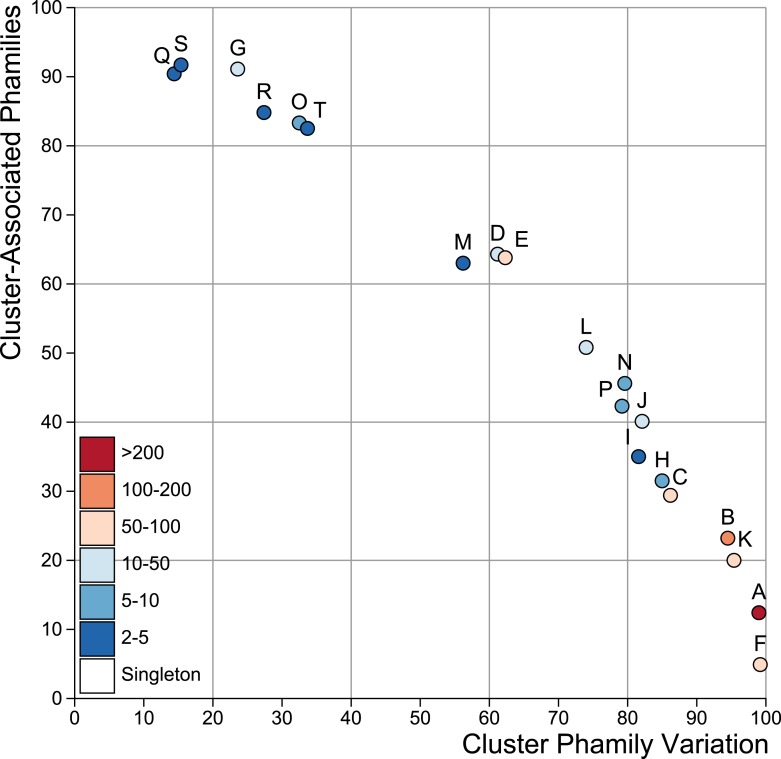
Cluster diversity shown by Cluster-Associated Phamilies (CAP) and Cluster Phamily Variation (CPV) indices. The CAP and CPV values are plotted for each cluster. **DOI:** http://dx.doi.org/10.7554/eLife.06416.021

We quantified the cluster diversity using three different measures, CLuster Average Shared Phamilies (CLASP), Cluster Associated Phamilies (CAP), and Cluster Cohesion Index (CCI) (Tables 1, 2, Figure 6A). Both CAP (the number of phams present in *all* genomes within a cluster divided by the average number of genes per genome) and CCI (the average number of genes per genome as a percentage of the total number of phams in that cluster) show substantial variation between clusters (Table 1, S2), and little evidence for commonly conserved ‘core genes’, as suggested for T4-related phages (Petrov et al., 2010). However, both of these parameters are somewhat influenced by cluster/subcluster size, which varies from cluster to cluster. In contrast, CLASP (the percentage of phamilies shared between two genomes, then averaged across all possible pairs within a cluster or subcluster) is relatively insensitive to cluster/subcluster size (as seen by a resampling analysis; Figure 6—figure supplement 1), but still shows substantial variation from one cluster to another (Table 1, Figure 6A).

**Table 2.** Genometrics and Cluster Cohesion Indexes of mycobacteriophages **DOI:** http://dx.doi.org/10.7554/eLife.06416.022

Cluster	Subcluster	# Genomes	Average # genes	Average length (bp)	# Phams	CLASP*	CAP†	CCI‡
A		232	90.0	51,514	1085	38.3	12.4	8.0
	A1	72	91.2	51,954	416	72.3	36.9	22.0
	A2	28	93.4	52,805	312	64.7	30.1	30.0
	A3	37	87.7	50,325	163	81.1	48.8	54.0
	A4	46	87.4	51,376	125	92.7	70.6	70.0
	A5	16	86.0	50,531	152	81.4	58.7	57.0
	A6	11	97.8	51,677	128	90.2	75.1	76.0
	A7	3	84.3	52,941	115	74.9	64.4	73.0
	A8	4	97.8	51,597	107	93.5	86.8	91.0
	A9	4	96.0	52,838	106	92.7	83.4	91.0
	A10	7	80.0	49,174	112	81.6	60.9	71.0
	A11	4	98.5	52,260	113	93.6	88.3	87.0
B		108	100.4	68,653	421	66.2	23.2	24.0
	B1	77	101.8	68,532	144	93.2	72.9	71.0
	B2	8	89.9	67,267	101	94.9	84.6	89.0
	B3	12	102.8	68,698	121	96.3	84.7	85.0
	B4	8	96.1	70,619	166	79.9	45.8	58.0
	B5	3	96.3	70,033	108	91.7	87.2	89.0
C		45	231.0	155,504	486	89.3	29.4	48.0
	C1	44	231.0	155,297	345	91.9	73.2	67.0
	C2	1	229.0	164,602	227	N/A	N/A	N/A
D		10	89.3	64,965	147	88.1	64.3	61.0
	D1	9	87.3	64,697	100	94.9	88.8	87.0
	D2	1	107.0	67,383	107	N/A	N/A	N/A
E		35	141.9	75,526	235	87.2	63.8	60.0
F		66	105.3	57,416	658	54.4	4.9	16.0
	F1	60	104.8	57,486	573	59.6	20.6	18.0
	F2	5	110.8	55,996	207	65.7	49.0	54.0
	F3	1	107.0	60,285	105	N/A	N/A	N/A
G		14	61.5	41,845	72	96.0	91.1	85.0
H		5	98.4	69,469	207	61.6	31.5	48.0
	H1	4	95.8	69,137	131	81.9	67.9	73.0
	H2	1	109.0	70,797	110	N/A	N/A	N/A
I		4	78.0	49,954	147	58.9	35.0	53.0
	I1	3	76.0	47,588	101	77.5	66.7	75.0
	I2	1	84.0	57,050	84	N/A	N/A	N/A
J		16	239.8	110,332	530	70.8	40.1	45.0
K		33	95.7	59,720	411	51.8	20.0	23.0
	K1	15	94.3	59,877	166	85.5	47.9	57.0
	K2	4	96.3	56,597	128	85.2	77.7	75.0
	K3	3	98.2	61,322	111	92.2	89.5	88.0
	K4	5	94.0	57,865	106	93.7	87.2	89.0
	K5	6	98.2	62,154	144	82.1	68.2	68.0
L		13	127.9	75,177	246	78.2	50.8	52.0
	L1	3	123.7	74,050	135	92.6	88.8	92.0
	L2	9	129.3	75,456	170	90.1	72.2	76.0
	L3	1	128.0	76,050	126	N/A	N/A	N/A
M		3	141.0	81,636	201	73.5	63.0	70.0
	M1	2	135.0	80,593	138	96.6	96.6	98.0
	M2	1	153.0	83,724	152	N/A	N/A	N/A
N		7	69.1	42,888	152	64.1	45.6	45.0
O		5	124.2	70,651	151	90.6	83.3	82.0
P		9	78.8	47,668	159	76.1	42.3	50.0
	P1	8	78.4	47,313	126	82.9	52.9	62.0
	P2	1	82.0	50,513	82	N/A	N/A	N/A
Q		5	85.2	53,755	90	96.6	90.4	95.0
R		4	101.5	71,348	117	91.4	84.8	87.0
S		2	109.0	65,172	117	91.7	91.7	93.0
T		3	66.7	42,833	83	86.1	82.5	80.0

* Cluster Averaged Shared Phamilies.

† Cluster Associated Phamilies.

‡ Cluster Cohesion Index.

### The discreteness of different clusters is highly varied

The heat map of genome comparisons (Figure 5) also illustrates the degrees to which clusters and subclusters share gene content, a reflection of cluster discreteness, or how isolated discrete clusters are from each other. For example, although the Cluster A phages are highly diverse, they also appear relatively isolated and share relatively few genes with other clusters (Figure 5). In contrast, phages in Cluster E share substantial numbers of genes with other clusters, including those in Clusters F, J, L, P, and several singletons. We have quantified these relationships with the Cluster Isolation Index (CII, the percentage of phams present within a cluster that are not present in other mycobacteriophage genomes), which demonstrates the considerable variation in isolation from phages of other clusters/subclusters (Table 1, Figure 6B). For example, at one extreme, 84.6% of Cluster C gene phamilies are found only in Cluster C and not elsewhere. At the other extreme, only 23.8% of Cluster I gene phamilies are constrained to that cluster, with the remainder having relatives present in genomes in other clusters. Other clusters form a spectrum of relationships between these extremes (Table 1, Figure 6B), and clusters such as I and P—which share recognizable DNA sequence similarity (Figure 2—figure supplement 1)—share >60% of their genes with other phages (low CII values; Table 1). Thus, although some clusters could be considered as discrete groups—as reported for the *Synechococcus* phages (Deng et al., 2014)—this is far from being a universal or characteristic feature of groups of related phages.

Cluster isolation analyses reveal additional complexities arising from highly mosaic genomes. For example, the singleton Dori is clearly related to Cluster B phages (Figure 3) with which it shares limited DNA similarity (Figure 2—figure supplement 2) with 20–26% of its genes (Figure 4—figure supplement 1), but also has nucleotide similarity and shares genes with Cluster N and I2 phages, among others (Figure 2—figure supplement 2, Figure 4—figure supplement 1), as reflected in its low CII (Table 1, Figure 6B). Likewise, the singleton MooMoo has segments of DNA similarity and shares ∼20% of its gene content (as determined by shared phams) with Cluster F phages (Figure 3, Figure 2—figure supplement 3, Figure 4—figure supplement 1), but also has similarity to Clusters N and I, as well as a low CII (Table 1, Figure 6B). It has low DNA similarity to Cluster O (Figure 2—figure supplement 3), but has several phams in common with the Cluster O phages, and has the same unusual prolate morphology (Figure 3). Complex relationships are also seen in the singletons Gaia and Sparky (Figure 4—figure supplement 2).

Taken together, the analyses of both cluster diversity and cluster isolation show that mycobacteriophage populations contain a continuum of diversity, with non-uniform abundance of different types of phages. The prevalence of isolated phages may not necessarily reflect the proportions of different types of phages in the environment, but the availability of a large collection of isolated phages enables capture and whole genome analysis of relatively rare phages that are critical to understanding the complexities of genome relationships. We recently reported genomic analysis of the singleton mycobacteriophage Patience, which has a substantially lower GC% than its host (50.3% vs 67.4%), has a different codon usage profile, but is undergoing codon selection for growth in a high GC% environment (Pope et al., 2014b). If there is a flux of phage genomes and genes entering the mycobacterial neighborhood, then we predict that the phages of a single host do not reflect a closed system with discrete populations, but one that is open with ever-expanding diversity.

### The mycobacteriophage population is not a closed system

**Figure 7.**
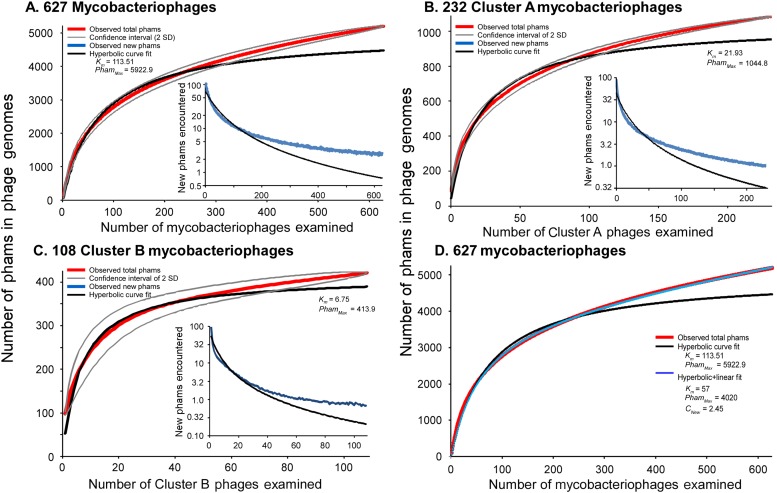
Rarefaction analysis of mycobacteriophage genomes. (**A**) The numbers of phamilies are reported for between 1 and 627 phage genomes sampled at random without replacement; the mean of 10,000 iterations is shown in red; gray lines indicate a confidence interval of two standard deviations. The black line shows a hyperbolic curve fit to the data from phage counts 1 to 314. The inset shows the number of new phams encountered upon the inclusion of each phage, with the mean number for the 10,000 iterations shown in blue and the predicted value from the hyperbolic curve shown in black. (**B**) Rarefaction analysis of 232 Cluster A phages. The total numbers of phamilies are reported for between 1 and 232 phages sampled at random without replacement from Cluster A; the mean of 10,000 iterations is shown in red; gray lines indicate a confidence interval of two standard deviations. The black line shows a hyperbolic curve fit to the data from phage counts 1 to 117. The inset shows the number of new phams encountered upon the inclusion of each phage, with the mean number for 10,000 iterations shown in blue and the predicted value from the hyperbolic curve shown in black. (**C**) Rarefaction analysis of 108 Cluster B phages; the hyperbolic curve was fit to the data from phage counts 1 to 54. (**D**) Fits of the hyperbolic (Equation 1) and hyperbolic with linear (Equation 2) models for phamily identification within genome samples. **DOI:** http://dx.doi.org/10.7554/eLife.06416.023 10.7554/eLife.06416.024Figure 7—source data 1.Datasets for determination of rarefaction curves.**DOI:** http://dx.doi.org/10.7554/eLife.06416.024

Both the huge diversity of phamilies in mycobacteriophages and the high frequency of orphams suggest that genes are constantly added to phage genomes from outside sources just as genes are added to the genomes of their bacterial hosts via horizontal gene transfer. Such gene influx—for example, from host-jumping phages such as Patience (Pope et al., 2014b)—would provide genetic novelty and enable phages to adapt to their ever-changing hosts. To examine gene flux into the mycobacteriophage population, we performed a rarefaction analysis by re-sampling the gene phamilies within the phage population (Figure 7). Remarkably, the rarefaction curves of the entire collection—including the 95% confidence limits—do not fit a hyperbola as would be expected if the mycobacteriophages were limited to an isolated set of genes, and about 2.5 new gene phamilies are predicted to be identified with each newly isolated phage (Figure 7A). Similar independent analyses on the phages of Cluster A or the phages of Cluster B show that this is also observed within these clusters (Figure 7B,C). Thus both individual clusters and the collection as a whole are not genetically fixed, but are in constant flux. While a hyperbola can model sampling of gene phamilies from a finite pool, it does not accommodate the influx of new phamilies. The addition of a linear term (see ‘Materials and methods’), representing the introduction of new phamilies from outside sources, results in a non-asymptotic curve which predicts the continual identification of new phams even after large numbers of genomes have been sampled (R > 0.999; Figure 7D). This linear term acts as a surrogate for the linear range of a second hyperbolic curve, one representing the resampling of a much larger set of gene phamilies available for introduction into mycobacteriophage genomes. Unfortunately, the current dataset remains insufficient to confidently extrapolate to give an estimate of the total number of viral protein families in the biosphere, which has been previously estimated to be anywhere between a half a million and 2 billion (Rohwer, 2003; Ignacio-Espinoza et al., 2013).

We note that because of the generally slow pace of the advancement of phage genomics, we have little insight into the phage populations of other hosts. We retrieved all double-stranded DNA tailed phage genomes in GenBank that we could identify (a total of 1781), corresponding to about 120 host bacterial genera, with a median number of phages per host genus of two. Using similar parameters for pham building as described above, the 181,717 predicted genes assemble into 47,479 phamilies. The relatively low representation of each phamily (3.8 genes/phamily) compared to the mycobacteriophages (13.4 genes/phamily) is a further reflection of the gross under-sampling of the phage population as a whole.

### Implications for bacteriophage taxonomy

Bacteriophage taxonomic classification reflecting phylogeny presents substantial challenges because of genome mosaicism (Lawrence et al., 2002). Classification by viral morphology is well established, but may not accurately reflect the genetic relationships, as illustrated for the prolate-headed MooMoo (Figure 3). We also note that the mycobacteriophage myoviruses have a high CII and form a discrete group (Table 1) as do the *Synechococcus* myophages (Deng et al., 2014), perhaps reflecting a virulent lifestyle that constrains productive gene exchange; T4-related phages from diverse hosts share a core set of 15–20% of their genes, and whole genome comparisons reveal extensive mosaicism (Petrov et al., 2010). Host range mutability thus may differ in phages with different morphotypes, limiting access to the gene pool, and although grouping phages into clusters and subclusters provides analytical advantages because of the wide range in prevalence of different phages (Table 1), it is not suitable as a broadly applicable hierarchical taxonomic system. The comparative analysis of these mycobacteriophages thus supports reticulate taxonomies that more accurately reflect the phylogenetic complexities (Lawrence et al., 2002; Lima-Mendez et al., 2007).

### Implications for student learning through research experiences

A research experience can be a powerful vehicle that enables a person to gain an understanding of the process of science (Hunter et al., 2007). When the research experience occurs early and at a large scale, as described here, the focus can shift from selecting a few ‘qualified’ students to exploring the potential interests of many students. Clearly, an essential ingredient is the nature of the research project, as definitions of research may vary from an inquiry-based exercise to authentic research with the potential to contribute publishable findings. To optimize the educational benefits, the research project must be intellectually and technically accessible to beginning students (i.e., few prerequisites) and scalable so that many students can simultaneously make progress in parallel, yet independently (Hatfull et al., 2006). Importantly, each student's findings should contribute to a scientific question with integration of all students' discoveries advancing a scientific question of significance, as judged by scientific peer review. This, we believe, defines an ‘authentic’ research experience. We note that in the SEA-PHAGES platform, substantial student effort is invested in arriving at high-quality genome annotations by close manual inspection followed by expert verification, a critical component of the detailed comparative analysis of phage gene content described here.

### Concluding comments

Bacteriophage genomics has progressed relatively slowly compared to that of other microbes in spite of their relatively small genome sizes. Here we have demonstrated that programmatically integrating the research and education missions at large scale provides an effective solution to expanding our knowledge of viral diversity, with a multitude of insights gained as a consequence of the scale of phage discovery. The nature of different genomic types, the variations of the diversity both within clusters and shared genome content among clusters, and the expanse of the mycobacteriophage population can be viewed at an unprecedented level of resolution. Our conclusions align well with comparative analyses of phages of *Enterobacteriacea* (Grose and Casjens, 2014) and *Bacillus* spp. (Grose et al., 2014) and we predict that these are general parameters of bacteriophage diversity, at least when sampling broadly across the environment. Both the rarefaction analysis described here and preliminary analysis of phamilies of all sequenced DNA phages illustrate how little of the global phage population has been genomically sampled. With a near endless supply of diverse viruses readily accessible for isolation and analyses, integrated research/education programs will continue to play substantial roles in defining the nature of the virosphere.

## Materials and methods

### Phages and genomes

In addition to extant GenBank sequence information, mycobacteriophages were isolated, sequenced, and annotated in the Phage Hunters Integrating Research and Education (PHIRE) or SEA-PHAGES programs. Phage genomes were shotgun sequenced using either 454, Ion Torrent, or Illumina platforms to at least 20-fold coverage. Shotgun reads were assembled de novo with Newbler versions 2.1 to 2.9. Assemblies were checked for low coverage or discrepant areas, and targeted Sanger reads were used to resolve weak areas and identify genome ends. All genome sequences are publically available at phagesDB.org or in GenBank. Nucleotide comparisons used BLASTN or Gepard (Krumsiek et al., 2007).

### Database construction

To create Phamerator database Mykobacteriophage_627, phamilies were constructed by first clustering the entire database of 69,574 genes using strict kClust parameters (70% clustering threshold and 0.25 alignment coverage of the longer sequence). This was followed by multiple sequence alignment of each preliminary cluster using Kalign (Lassmann and Sonnhammer, 2005). Consensus sequences were then extracted using HHmake and HHconsensus (Remmert et al., 2012). The resulting list of sequences was subjected to a second—and less strict—round of clustering via kClust (30% clustering threshold and 0.5 alignment coverage of the longer sequence) to obtain the final phamily assignments.

Network phylogeny constructions were made using the NeighborNet function with default parameters in SplitsTree (Huson, 1998; Huson and Bryant, 2006).

### Cluster diversity and isolation indices

Four parameters were used to evaluate cluster diversity. The first is the CLASP index that calculates the percentage of phamilies shared between two genomes, then averages across all possible pairs within a cluster or subcluster. Because the pairwise similarities are averaged, CLASP is relatively insensitive to either the overall size of the cluster, or the heterogeneity of its diversity (such as in Cluster C in which of the 45 genomes in total, 44 are in Cluster C1, and only one is in Cluster C2). CLASP robustness with respect to cluster size was demonstrated through a resampling analysis. For each cluster with more than 30 members, a random subset (of 5, 10, 20, or 30 genomes) was selected and CLASP was calculated. For each sample size, 20 iterations were performed with replacement. As expected, there is substantial deviation among the iterations, especially at smaller sizes. However, there is little change in the average CLASP values with different sample sizes (Figure 4—figure supplement 1), showing that cluster size is not a primary driver of diversity. The resampling analyses also suggest that while a greater number of genomes helps refine the CLASP value, there is still predictive power when only 10 genomes are compared. On average, the maximum and minimum iteration values at a sample size of 10 genomes were within 8% of the whole-cluster CLASP value. This implies that, for example, increasing Cluster D from 10 to 50 or 100 genomes may raise or lower its current CLASP value of 88.1, but that value is likely to remain between ∼80 and ∼96.

The second measure used is the CAP, which is calculated as the number of phamilies present in *all* genomes within a cluster divided by the average number of phamilies per genome. These cluster-conserved genes could correspond to core genes that define a particular phage group such as cluster or subcluster. However, for those clusters with sufficient diversity to detect such core genes, these values are low. For example, among the 66 Cluster F genomes, only five phamilies are present in all genomes. None are virion structural genes, one is a glycosyltransferase whose role is unknown, one is a putative regulator, and the others are small proteins of unknown function. For the Cluster A genomes, 11 phamilies are conserved, seven of which are virion structural proteins, three are involved in DNA metabolism (DNA Pol, Helicase, Rec-Like protein), and one is of unknown function.

The third parameter is the Cluster Phamily Variation (CPV) index, which is the proportion of phams that are not present in all members of the cluster. CAP and CPV are inversely related but imperfectly as CPV varies with cluster size even among similarly diverse clusters; a plot of CAP values against CPV values is shown in Figure 6—figure supplement 2.

The CCI is calculated as the average number of genes per genome as a percentage of the total number of phams in that cluster. Thus if all genomes in a cluster are identical (and if phamilies occur only once in a genome), CCI would be 100; the CCI for two sets of five randomly chosen genomes is ∼2. CCI values correlate with cluster size, but similarly sized clusters as such G, J, and L, or E and K have substantially different CCI values (Table 1).

The CII is the percentage of phams present within a cluster that are not present in other mycobacteriophage genomes.

### Rarefaction analysis

Rarefaction analysis was performed by randomly selecting subsets (without replacement) of between 1 and 627 (all), 232 (Cluster A) or 108 (Cluster B) mycobacteriophages and determining the numbers of phamilies represented. This was repeated 10,000 times to generate a mean number of phamilies observed given a number of phage genomes selected. The means of the accumulated numbers of phams and the numbers of new phages identified are plotted as the function of the number of genomes selected at random. The observed numbers were fit to a hyperbolic function for 50% of the sample (i.e., 1 to 314, 116 or 54 genomes for all, Cluster A or Cluster B phages, respectively); Hanes-Woolf regression was used to estimate *Pham*_*Max*_ and *K*_*m*_ of the hyperbola:

(1) $$N_{Phams}=\frac{Pham_{Max}\times N_{Genomes}}{K_{m}+N_{Genomes}},$$

where *N*_*Genomes*_ is the number of genomes sampled, *N*_*Phams*_ is the number of total phams seen within those genomes, *Pham*_*Max*_ is the total number of phams among all mycobacteriophage genomes, and *K*_*m*_ is the number of genomes required to sample one half of *Pham*_*Max*_.

The lack of fit of the observed data to the hyperbola—with the observed data reflecting infinite size—suggests that the overall population is dynamic. The lack of hyperbolic fit of the data does not result from outliers such as phages with highly deviant GC%, because removing these does not improve the fit. The fit is also not substantially improved by analysis of the two largest clusters, Cluster A and Cluster B (Figure 7), suggesting that the dynamic nature of the gene pool is not an artifact of examining independent phage clusters with separate gene pools.

To model this behavior, we modified Equation 1 to include the introduction of novel phams via recombination with outside, non-mycobacteriophage genomes:

(2) $$N_{Phams}=N_{Genomes}\times C_{Phage}+\frac{Pham_{Max}\times N_{Genomes}}{K_{m}+N_{Genomes}},$$

where *C*_*Phage*_ is the number of outside phams seen in each phage. The value of *C*_*Phage*_ was estimated from Figure 7B and new values for *Pham*_*Max*_ and *K*_*Pham*_ were estimated by Hanes-Woolf regression following data normalization.

## Additional files

Supplementary file 1. List of 627 sequenced mycobacteriophages and cluster designations. **DOI:** http://dx.doi.org/10.7554/eLife.06416.025

Supplementary file 2. Full list of group author details. **DOI:** http://dx.doi.org/10.7554/eLife.06416.026

### Major datasets

The following datasets were generated:

Russell D, Hatfull G, 2015, Mycobacteriophage database, http://phagesdb.org, Publicly available at the Mycobacteriophage Database (http://phagesdb.org).

Bowman C, Cresawn S, Hatfull G, 2014, Mykobacteriophage_627, http://phamerator.webfactional.com/databases_Hatfull, Accessed via the program Phamerator, publicly available at http://phagesdb.org/Phamerator/faq/.
