# Supplementary material for: Whole genome comparison of a large collection of mycobacteriophages reveals a continuum of phage genetic diversity
Source: eLife. 2015 Apr 28;4:e06416. doi: 10.7554/eLife.06416 (PMC4408529; doi:10.7554/eLife.06416)
Supplement: Supplementary file 1. — List of 627 sequenced mycobacteriophages and cluster designations. DOI: http://dx.doi.org/10.7554/eLife.06416.025 [file elife-06416-supp1.docx]

| **Phage Name** | **Clus** |
| --- | --- |
| Abrogate | A1 |
| Aeneas | A1 |
| Alsfro | A1 |
| Anglerfish | A1 |
| Arcanine | A1 |
| BPBiebs31 | A1 |
| BeesKnees | A1 |
| Bethlehem | A1 |
| BillKnuckles | A1 |
| Bob3 | A1 |
| Bruns | A1 |
| Bxb1 | A1 |
| ConceptII | A1 |
| Corvo | A1 |
| DD5 | A1 |
| Doom | A1 |
| Dreamboat | A1 |
| Dynamix | A1 |
| Edtherson | A1 |
| Euphoria | A1 |
| Fascinus | A1 |
| Forsytheast | A1 |
| Fushigi | A1 |
| GageAP | A1 |
| Hope4ever | A1 |
| Ichabod | A1 |
| JC27 | A1 |
| Jasper | A1 |
| KBG | A1 |
| KSSJEB | A1 |
| Kugel | A1 |
| Kykar | A1 |
| Lamina13 | A1 |
| Lesedi | A1 |
| Lockley | A1 |
| MPlant7149 | A1 |
| Magnito | A1 |
| Manatee | A1 |
| Marcell | A1 |
| McGuire | A1 |
| MetalQZJ | A1 |
| MrGordo | A1 |
| Museum | A1 |
| Papez | A1 |
| Pari | A1 |
| PattyP | A1 |
| Pepe | A1 |
| Perseus | A1 |
| Petp2012 | A1 |
| PhrostyMug | A1 |
| Pinto | A1 |
| RidgeCB | A1 |
| Ringer | A1 |
| Rufus | A1 |
| Ruotula | A1 |
| Rutherferd | A1 |
| Sarfire | A1 |
| Scowl | A1 |
| SkiPole | A1 |
| Solon | A1 |
| Switzer | A1 |
| Target | A1 |
| Thor | A1 |
| Treddle | A1 |
| Tripl3t | A1 |
| Trouble | A1 |
| Turj99 | A1 |
| U2 | A1 |
| Violet | A1 |
| Wheeler | A1 |
| Zephyr | A1 |
| Zeuska | A1 |
| ADZZY | A2 |
| Bugsy | A2 |
| Changeling | A2 |
| Che12 | A2 |
| ChipMunk | A2 |
| D29 | A2 |
| EagleEye | A2 |
| Echild | A2 |
| Equemioh13 | A2 |
| EvilGenius | A2 |
| Heffalump | A2 |
| IronMan | A2 |
| Jerm | A2 |
| Jsquared | A2 |
| L5 | A2 |
| Larenn | A2 |
| Loser | A2 |
| Odin | A2 |
| Piro94 | A2 |
| Power | A2 |
| Pukovnik | A2 |
| RedRock | A2 |
| SemperFi | A2 |
| Serenity | A2 |
| SweetiePie | A2 |
| Trixie | A2 |
| Turbido | A2 |
| Whabigail7 | A2 |
| Aglet | A3 |
| Bxz2 | A3 |
| DaHudson | A3 |
| EpicPhail | A3 |
| Farber | A3 |
| GingkoMaracino | A3 |
| Grum1 | A3 |
| Hercules11 | A3 |
| JHC117 | A3 |
| Jobu08 | A3 |
| Lilith | A3 |
| Mainiac | A3 |
| MarQuardt | A3 |
| Marie | A3 |
| Methuselah | A3 |
| Microwolf | A3 |
| Misomonster | A3 |
| Ollie | A3 |
| P28Green | A3 |
| Phoxy | A3 |
| PotatoSplit | A3 |
| PurpleHaze | A3 |
| Sabia | A3 |
| Spike509 | A3 |
| Taurus | A3 |
| Tiffany | A3 |
| Vix | A3 |
| Zetzy | A3 |
| BabyRay | A31 |
| HelDan | A31 |
| Norbert | A31 |
| Phantastic | A31 |
| Pocahontas | A31 |
| Popcicle | A31 |
| QuinnKiro | A31 |
| Rockstar | A31 |
| Veracruz | A31 |
| Abdiel | A4 |
| Achebe | A4 |
| Arturo | A4 |
| Backyardigan | A4 |
| BellusTerra | A4 |
| Broseidon | A4 |
| Bruiser | A4 |
| BubbleTrouble | A4 |
| Burger | A4 |
| Caelakin | A4 |
| Camperdownii | A4 |
| Clarenza | A4 |
| Dhanush | A4 |
| Eagle | A4 |
| Eris | A4 |
| Flux | A4 |
| Funston | A4 |
| Gadost | A4 |
| HamSlice | A4 |
| Holli | A4 |
| ICleared | A4 |
| KFPoly | A4 |
| Kampy | A4 |
| Kratark | A4 |
| LHTSCC | A4 |
| Lemur | A4 |
| LittleGuy | A4 |
| Maverick | A4 |
| Medusa | A4 |
| MeeZee | A4 |
| Melvin | A4 |
| Millski | A4 |
| Morpher26 | A4 |
| Mundrea | A4 |
| Nyxis | A4 |
| Obama12 | A4 |
| Peaches | A4 |
| Phighter1804 | A4 |
| Pipcraft | A4 |
| Sabertooth | A4 |
| Shaka | A4 |
| TinaFeyge | A4 |
| TiroTheta9 | A4 |
| TygerBlood | A4 |
| Wander | A4 |
| Wile | A4 |
| Airmid | A5 |
| Aragog | A5 |
| Archetta | A5 |
| Benedict | A5 |
| Chadwick | A5 |
| Cuco | A5 |
| ElTiger69 | A5 |
| ForGetIt | A5 |
| George | A5 |
| LittleCherry | A5 |
| Naca | A5 |
| Phlorence | A5 |
| Swirley | A5 |
| Theia | A5 |
| Tiger | A5 |
| UnionJack | A5 |
| Blue7 | A6 |
| DaVinci | A6 |
| EricB | A6 |
| Gladiator | A6 |
| Hammer | A6 |
| Jeffabunny | A6 |
| JewelBug | A6 |
| Kazan | A6 |
| McFly | A6 |
| SuperAwesome | A6 |
| VohminGhazi | A6 |
| HINdeR | A7 |
| Sheen | A7 |
| Timshel | A7 |
| Astro | A8 |
| Expelliarmus | A8 |
| Saintus | A8 |
| Smeadley | A8 |
| Alma | A9 |
| Catalina | A9 |
| Myxus | A9 |
| PackMan | A9 |
| Goose | A10 |
| KittenMittens | A10 |
| Rebeuca | A10 |
| RhynO | A10 |
| Severus | A10 |
| Trike | A10 |
| Twister | A10 |
| Bachome | A11 |
| Et2Brutus | A11 |
| Fibonacci | A11 |
| Mulciber | A11 |
| Adjutor | D1 |
| BigMama | D1 |
| Butterscotch | D1 |
| Gumball | D1 |
| Nova | D1 |
| PBI1 | D1 |
| PLot | D1 |
| SirHarley | D1 |
| Troll4 | D1 |
| Hawkeye | D2 |
| 244 | E |
| ABCat | E |
| Bask21 | E |
| Cactus | E |
| Cjw1 | E |
| Contagion | E |
| Czyszczon1 | E |
| DrDrey | E |
| Dumbo | E |
| Dusk | E |
| Elph10 | E |
| Eureka | E |
| Goku | E |
| Henry | E |
| Hopey | E |
| Kostya | E |
| Lilac | E |
| MadamMonkfish | E |
| Murphy | E |
| NelitzaMV | E |
| NoSleep | E |
| Pharsalus | E |
| Phaux | E |
| Phrux | E |
| Porky | E |
| Pumpkin | E |
| Rakim | E |
| RiverMonster | E |
| Simpliphy | E |
| SirDuracell | E |
| Stark | E |
| TeardropMSU | E |
| Toto | E |
| Tuco | E |
| Ukulele | E |
| Ardmore | F1 |
| Batiatus | F1 |
| Bipolar | F1 |
| Bobi | F1 |
| Boomer | F1 |
| Brocalys | F1 |
| Bubbles123 | F1 |
| BuzzLyseyear | F1 |
| Cabrinians | F1 |
| CaptainTrips | F1 |
| Cerasum | F1 |
| Che8 | F1 |
| DLane | F1 |
| Daenerys | F1 |
| Dante | F1 |
| DeadP | F1 |
| Dorothy | F1 |
| DotProduct | F1 |
| Drago | F1 |
| Empress | F1 |
| Estave1 | F1 |
| Fruitloop | F1 |
| GUmbie | F1 |
| Girr | F1 |
| Hades | F1 |
| Hamulus | F1 |
| Hegedechwinu | F1 |
| Ibhubesi | F1 |
| Inventum | F1 |
| Job42 | F1 |
| Krakatau | F1 |
| Llama | F1 |
| Llij | F1 |
| Mantra | F1 |
| MilleniumForce | F1 |
| Minnie | F1 |
| MisterCuddles | F1 |
| Mozy | F1 |
| Mutaforma13 | F1 |
| Ogopogo | F1 |
| Ovechkin | F1 |
| PMC | F1 |
| Pacc40 | F1 |
| Pippy | F1 |
| Ramsey | F1 |
| RockyHorror | F1 |
| Ruby | F1 |
| SG4 | F1 |
| Saal | F1 |
| Shauna1 | F1 |
| ShiLan | F1 |
| SiSi | F1 |
| Spartacus | F1 |
| Spoonbill | F1 |
| SuperGrey | F1 |
| Taj | F1 |
| Tweety | F1 |
| Velveteen | F1 |
| Wee | F1 |
| dirtMcgirt | F1 |
| Avani | F2 |
| Che9d | F2 |
| Jabbawokkie | F2 |
| Yoshi | F2 |
| Zapner | F2 |
| Squirty | F3 |
| Angel | G |
| Annihilator | G |
| Avrafan | G |
| BPs | G |
| BQuat | G |
| BruceB | G |
| Cherrybomb426 | G |
| Frosty24 | G |
| Gomashi | G |
| Halo | G |
| Hope | G |
| Liefie | G |
| Phreak | G |
| Zombie | G |
| Damien | H1 |
| Konstantine | H1 |
| Oaker | H1 |
| Predator | H1 |
| Barnyard | H2 |
| Babsiella | I1 |
| Brujita | I1 |
| Island3 | I1 |
| Che9c | I2 |
| Ariel | J |
| BAKA | J |
| Courthouse | J |
| Duke13 | J |
| EricMillard | J |
| Halley | J |
| Klein | J |
| LittleE | J |
| Lucky2013 | J |
| MiaZeal | J |
| Minerva | J |
| Omega | J |
| Optimus | J |
| Redno2 | J |
| Thibault | J |
| Wanda | J |
| Adephagia | K1 |
| Amelie | K1 |
| Anaya | K1 |
| Angelica | K1 |
| BEEST | K1 |
| BarrelRoll | K1 |
| CREW | K1 |
| CrimD | K1 |
| Emerson | K1 |
| Homura | K1 |
| JAWS | K1 |
| Joy99 | K1 |
| Murucutumbu | K1 |
| Sulley | K1 |
| Validus | K1 |
| Milly | K2 |
| Mufasa | K2 |
| TM4 | K2 |
| ZoeJ | K2 |
| Keshu | K3 |
| MacnCheese | K3 |
| Pixie | K3 |
| Cheetobro | K4 |
| Fionnbharth | K4 |
| SamScheppers | K4 |
| Slarp | K4 |
| Taquito | K4 |
| Collard | K5 |
| Gengar | K5 |
| Kratio | K5 |
| Larva | K5 |
| OkiRoe | K5 |
| Omnicron | K5 |
| JoeDirt | L1 |
| LeBron | L1 |
| UPIE | L1 |
| Archie | L2 |
| Breezona | L2 |
| Crossroads | L2 |
| Faith1 | L2 |
| Loadrie | L2 |
| MkaliMitinis3 | L2 |
| Nicholasp3 | L2 |
| Rumpelstiltskin | L2 |
| Winky | L2 |
| Whirlwind | L3 |
| Bongo | M |
| PegLeg | M |
| Rey | M |
| Butters | N |
| Carcharodon | N |
| Charlie | N |
| MichelleMyBell | N |
| Redi | N |
| SkinnyPete | N |
| Xerxes | N |
| DS6A | Sin |
| Dori | Sin |
| Gaia | Sin |
| MooMoo | Sin |
| Muddy | Sin |
| Patience | Sin |
| Sparky | Sin |
| Wildcat | Sin |
| Catdawg | O |
| Corndog | O |
| Dylan | O |
| Firecracker | O |
| YungJamal | O |
| Donovan | P1 |
| Fishburne | P1 |
| HUHilltop | P1 |
| Jebeks | P1 |
| Malithi | P1 |
| Phineas | P1 |
| Shipwreck | P1 |
| BigNuz | P1 |
| Purky | P2 |
| Evanesce | Q |
| Giles | Q |
| HH92 | Q |
| Kinbote | Q |
| OBUPride | Q |
| Nilo | R |
| Papyrus | R |
| Send513 | R |
| Weiss13 | R |
| Marvin | S |
| MosMoris | S |
| Bernal13 | T |
| Mendokysei | T |
| RonRayGun | T |
| ABU | B1 |
| Altwerkus | B1 |
| Apizium | B1 |
| Badfish | B1 |
| Banjo | B1 |
| BlackStallion | B1 |
| Chah | B1 |
| Chorkpop | B1 |
| Chunky | B1 |
| Colbert | B1 |
| Crownjwl | B1 |
| Daffy | B1 |
| DonSanchon | B1 |
| EmpTee | B1 |
| Eremos | B1 |
| Fang | B1 |
| FluffyNinja | B1 |
| FriarPreacher | B1 |
| Harvey | B1 |
| Held | B1 |
| Hertubise | B1 |
| Hetaeria | B1 |
| IsaacEli | B1 |
| JacAttac | B1 |
| KLucky39 | B1 |
| Kikipoo | B1 |
| KingVeveve | B1 |
| Kloppinator | B1 |
| Lasso | B1 |
| LeeLot | B1 |
| Lego3393 | B1 |
| LemonSlice | B1 |
| MRabcd | B1 |
| Mana | B1 |
| Manad | B1 |
| Megatron | B1 |
| MitKao | B1 |
| Morgushi | B1 |
| Morty | B1 |
| Mosaic | B1 |
| Murdoc | B1 |
| Newman | B1 |
| OSmaximus | B1 |
| Oline | B1 |
| OliverWalter | B1 |
| Oosterbaan | B1 |
| Orion | B1 |
| PG1 | B1 |
| Phipps | B1 |
| Pipsqueak | B1 |
| Puhltonio | B1 |
| Roscoe | B1 |
| SDcharge11 | B1 |
| Scoot17C | B1 |
| Serendipity | B1 |
| ShiVal | B1 |
| Sigman | B1 |
| Sophia | B1 |
| Soto | B1 |
| Spartan300 | B1 |
| Squid | B1 |
| Suffolk | B1 |
| Swish | B1 |
| TallGRassMM | B1 |
| Thora | B1 |
| ThreeOh3D2 | B1 |
| Trypo | B1 |
| UncleHowie | B1 |
| Vista | B1 |
| Vivaldi | B1 |
| Vortex | B1 |
| Waterdiva | B1 |
| Xavier | B1 |
| Yoshand | B1 |
| YouGoGlencoco | B1 |
| Zelda | B1 |
| Zonia | B1 |
| Arbiter | B2 |
| Ares | B2 |
| Hedgerow | B2 |
| Kheth | B2 |
| Laurie | B2 |
| LizLemon | B2 |
| Qyrzula | B2 |
| Rosebush | B2 |
| Akoma | B3 |
| Athena | B3 |
| Audrey | B3 |
| Compostia | B3 |
| Daisy | B3 |
| Gadjet | B3 |
| Heathcliff | B3 |
| Kamiyu | B3 |
| Phaedrus | B3 |
| Phlyer | B3 |
| Pipefish | B3 |
| Yahalom | B3 |
| Browncna | B4 |
| ChrisnMich | B4 |
| Cooper | B4 |
| Frederick | B4 |
| Nigel | B4 |
| Stinger | B4 |
| Zemanar | B4 |
| KayaCho | B41 |
| Acadian | B5 |
| Phelemich | B5 |
| Reprobate | B5 |
| Alice | C1 |
| ArcherS7 | C1 |
| Astraea | C1 |
| Ava3 | C1 |
| Bangla1971 | C1 |
| BeanWater | C1 |
| Breeniome | C1 |
| Bxz1 | C1 |
| Cali | C1 |
| Catera | C1 |
| CharlieB | C1 |
| DTDevon | C1 |
| Dandelion | C1 |
| Delilah | C1 |
| Drazdys | C1 |
| ET08 | C1 |
| EmToTheThree | C1 |
| ErnieJ | C1 |
| Ghost | C1 |
| Gizmo | C1 |
| LRRHood | C1 |
| LinStu | C1 |
| Littleton | C1 |
| MoMoMixon | C1 |
| Nappy | C1 |
| NuevoMundo | C1 |
| Pier | C1 |
| Pio | C1 |
| Pleione | C1 |
| QBert | C1 |
| Rizal | C1 |
| ScottMcG | C1 |
| Sebata | C1 |
| Shrimp | C1 |
| SmallFry | C1 |
| Spud | C1 |
| Teardrop | C1 |
| TinyTim | C1 |
| Tortoise16 | C1 |
| Tyke | C1 |
| Wally | C1 |
| Willis | C1 |
| Zeenon | C1 |
| ZygoTaiga | C1 |
| Myrna | C2 |
